# Neighborhood disadvantages and longitudinal progression of multimorbidity among adult survivors of childhood cancer: a single-center St. Jude Lifetime Cohort Study in the U.S.

**DOI:** 10.1016/j.eclinm.2026.104255

**Published:** 2026-10-13

**Authors:** Jaesung Choi, Madeline R. Horan, Sedigheh Mirzaei, Deo Kumar Srivastava, Nickhill Bhakta, Stephanie B. Dixon, Matthew J. Ehrhardt, Carmen L. Wilson, Angela Delaney, Yikyung Park, Daniel A. Mulrooney, Heather M. Brandt, Kirsten K. Ness, Gregory T. Armstrong, Melissa M. Hudson, I-Chan Huang

**Affiliations:** a Department of Epidemiology and Cancer Control, St. Jude Children’s Research Hospital, Memphis, TN, USA; b Department of Pediatrics, Wake Forest University School of Medicine, Winston-Salem, NC, USA; c Department of Biostatistics, St. Jude Children’s Research Hospital, Memphis, TN, USA; d Department of Global Pediatric Medicine, St. Jude Children’s Research Hospital, Memphis, TN, USA; e Department of Oncology, St. Jude Children’s Research Hospital, Memphis, TN, USA; f Australian Research Centre for Cancer Survivorship, University of New South Wales, Sydney, NSW, Australia; g Division of Endocrinology, Department of Pediatric Medicine, St. Jude Children’s Research Hospital, Memphis, TN, USA; h Department of Surgery, School of Medicine, Washington University in St. Louis, MO, USA; i HPV Cancer Prevention Program, St. Jude Children's Research Hospital, Memphis, TN, USA

**Keywords:** Social determinants of health, Neighborhood disadvantages, Chronic health conditions, Multimorbidity, Childhood cancer survivors

## Abstract

**Background:**

The impact of cancer therapy on chronic health condition (CHC) burden in childhood cancer survivors is well-established; however, the role of neighborhood-level social determinants of health (SDOH) remains underexplored.

**Methods:**

In this single-center cohort study in the U.S., we analyzed 2508 survivors enrolled in the St. Jude Lifetime Cohort Study between 9/13/2007 and 4/20/2020, who were ≥5 years from diagnosis, ≥18 years of age, and had ≥2 clinical assessments. SDOH encompassed social vulnerability (Social Vulnerability Index) and environmental/healthcare access vulnerabilities (e.g., primary care physician [PCP] availability, food inaccessibility), categorized into quartiles. A total of 177 clinically assessed CHCs were aggregated into 48 CHC groups and a global burden. Multivariable logistic regressions examined associations between baseline SDOH and CHC progression from baseline to follow-up, adjusting for clinical and sociodemographic factors. Global CHC burden progression was the prespecified primary outcome; analyses of specific CHC groups and effect modification by personal socioeconomic status were exploratory.

**Findings:**

Survivors residing in the most vs. least vulnerable neighborhoods, characterized by low income (odds ratio [OR]: 1.83, 95% confidence interval [CI]: 1.36–2.46), limited PCP availability (OR: 1.30, 95% CI: 1.03–1.64), and food inaccessibility (OR: 1.49, 95% CI: 1.08–2.07), had higher risks of global CHC burden progression. Living in SDOH-vulnerable neighborhoods was associated with higher risks of new-onset/worsening of multiple CHC groups, including low education for peripheral nervous system disorders (OR: 2.69, 95% CI: 1.63–4.43), food inaccessibility for arrhythmias (OR: 4.36, 95% CI: 1.32–14.35), and poor walkability for abnormal glucose metabolism (OR: 4.60, 95% CI: 1.41–15.04). Among publicly insured survivors, limited PCP availability and poor walkability were associated with higher risk of peripheral musculoskeletal disorder (OR: 6.11, 95% CI: 2.33–16.04, OR: 7.37, 95% CI: 2.82–19.27).

**Interpretation:**

SDOH are associated with CHC severity progression among childhood cancer survivors, supporting multilevel social risk assessment in survivorship care and further evaluation of neighborhood-informed risk stratification.

**Funding:**

U.S. National Cancer Institute.

**Research in context**

**Evidence before this study**

Childhood cancer survivors face a high risk of treatment-related late effects, including chronic health conditions (CHCs), poor quality-of-life, and premature mortality. Neighborhood-level social determinants of health (SDOH) are upstream drivers of health disparities, yet their impact on the evolution of late effects remains understudied. A comprehensive PubMed search was conducted from database inception to March 13, 2026, without language restrictions, using the terms: (“childhood cancer” OR “pediatric cancer” OR “adolescent and young adult cancer”) AND (“survivor∗” OR “patient∗” OR “survivorship”) AND ((“social determinants of health” OR “SDOH” OR “determinants of health” OR “social determinant∗” OR “social factor∗” OR “social risk” OR “socioeconomic” OR “adversity” OR “deprivation”) OR (“residence characteristics” OR “neighborhood characteristics” OR “neighborhood” OR “communit∗” OR “environment∗”)). We considered studies evaluating neighborhood or area-level social or environmental determinants in childhood, adolescent, or young adult cancer survivors. Previous studies linked neighborhood vulnerability to poorer quality-of-life, frailty, obesity, and premature mortality among childhood cancer survivors. However, the search yielded no studies comprehensively examining associations between neighborhood challenges, including socioeconomic deprivation or healthcare access, and the longitudinal progression of overall and organ-specific CHC burden in this population. The available evidence was largely cross-sectional and reliant on self-reported outcomes, limiting temporal inference, and heterogeneity in exposures and outcomes precluded a meaningful pooled estimate.

**Added value of this study**

This study is the first to comprehensively evaluate neighborhood-level SDOH domains, including poverty, housing conditions, healthcare accessibility, and the built environment, and their corresponding indicators in relation to longitudinally and clinically assessed CHC outcomes in a well-defined cohort of childhood cancer survivors. Leveraging clinically ascertained evaluations, this study advances a field that has traditionally relied on self-reported, limited measures of CHCs. Results suggest that residence in the most vulnerable neighborhoods was associated with approximately a 2-fold higher global multimorbidity progression and up to a 5-fold higher risk of specific cardiometabolic conditions. Modifiable factors such as limited primary care availability, food inaccessibility, and poor walkability were associated with new-onset and worsening CHCs across organ systems. Neighborhood disadvantages and personal socioeconomic vulnerability were jointly associated with up to a 7-fold higher risk of CHC progression.

**Implications of all the available evidence**

Childhood cancer survivors residing in socially and environmentally vulnerable neighborhoods face markedly greater risks of CHC progression across multiple organ systems. These findings suggest that integrating neighborhood-level context into survivorship care may enhance risk stratification and inform supportive care planning. Embedding social and environmental risk information into healthcare delivery could facilitate early identification of high-risk survivors and guide more tailored approaches. By linking neighborhood vulnerabilities to longitudinal health outcomes, this study lays the foundation for precision survivorship care and underscores the need for coordinated multi-level efforts to address long-term health disparities. Future prospective studies should evaluate whether integrating neighborhood SDOH into survivorship care improves clinical outcomes and reduces disparities.

## Introduction

The five-year overall survival for childhood cancers now approaches 85% in high-income countries but remains below 60% in many low- and middle-income countries.^1^ The growing population of long-term survivors faces a substantial burden of chronic health conditions (CHCs), including cardiovascular, respiratory, endocrine, musculoskeletal, and neurocognitive sequelae.^2^ CHCs can adversely affect survivors' quality-of-life^3^ and social and functional status.^4^

Extensive research has identified multiple risk factors for CHC burden among childhood cancer survivors, including cancer type, treatment modality, genetic predisposition, unhealthy lifestyle behaviors, and disadvantaged personal socioeconomic status (SES).^5^ Beyond individual-level factors, emerging evidence reveals that neighborhood-level social determinants of health (SDOH) influence general and disease-specific health outcomes,^6^, ^7^, ^8^ underscoring their relevance to survivorship. Healthy People 2030 defines SDOH as “the conditions in the environments where people are born, live, learn, work, play, worship, and age that affect a wide range of health, functioning, and quality-of-life outcomes and risks.”^9^ SDOH encompasses five domains: economic stability, educational access and quality, healthcare access, neighborhood and built environment, and social and community context. In the general population, adverse SDOH are associated with elevated risks of CHCs, particularly cardiovascular disease, diabetes, and cancer,^6^ increased all-cause mortality^7^ and persistent health inequities.^8^

Because treatment-related late effects span multiple organ systems in childhood cancer survivors,^2^ early or prolonged exposure to adverse SDOH may confer greater CHC burden and progression than in adult-onset cancer survivors. Although limited studies linked neighborhood vulnerabilities to poorer quality-of-life, frailty, obesity, and early mortality in this population,^10^, ^11^, ^12^, ^13^ evidence is largely cross-sectional and self-reported, limiting temporal inference. Moreover, prior research has primarily emphasized social aspects of neighborhood vulnerabilities (e.g., poverty, housing instability), with relatively little attention to physical- and built-environment conditions (e.g., walkability, healthcare accessibility) that shape daily exposures, health behaviors, and access to essential resources relevant to long-term health outcomes.

Disadvantaged personal SES (e.g., low income, limited education) may compound neighborhood-level vulnerabilities by limiting access to essential resources, such as nutritious food, reliable transportation, and quality healthcare.^14^ However, the joint effects of personal SES and neighborhood SDOH on long-term health trajectories in childhood cancer survivors remain understudied. Strengthening evidence on these interrelated determinants is crucial to inform tailored, place-based interventions to reduce CHC burden and promote health equity.

This study leverages longitudinal data from the St. Jude Lifetime Cohort (SJLIFE) study to evaluate associations between neighborhood-level social and environmental/healthcare access vulnerabilities and CHC severity progression in adult survivors of childhood cancer, while examining how these associations were modified by personal SES. Conceptually, neighborhood-level factors may influence CHC severity progression through multiple pathways, including healthcare access (e.g., primary care availability), built environment (e.g., food access, walkability), and psychosocial and biological processes (e.g., chronic stress, inflammation). These pathways are likely modified by personal SES (e.g., insurance, education), which may alter access to care and resilience towards neighborhood risks. Within this framework, we aim to generate hypothesis-driven evidence on the interplay between neighborhood and personal factors to inform precision strategies and more equitable resource allocation for high-risk survivors.

## Methods

### Study population

This study analyzed data from adult survivors of childhood cancer enrolled in SJLIFE, an ongoing retrospectively constructed cohort with prospective longitudinal follow-up of individuals treated at St. Jude Children’s Research Hospital (SJCRH) between 1962 and 2012 to track long-term health outcomes.^15^ Inclusion criteria required survivors to be at least five years post-diagnosis and 18 years or older at study enrollment (2007–2020). Survivors were clinically assessed and self-reported sociodemographic, lifestyle, and residential address information. Among the 4686 survivors enrolled in SJLIFE, 473 were excluded due to unavailable baseline survey or clinical assessment, or ungeocodable address data (Supplementary Fig. S1). Of the remaining 4213 survivors, 106 died before a second clinical assessment, and 1599 lacked a follow-up clinical assessment, leaving 2508 survivors for analysis (Supplementary Fig. S1).

### Ethics

The SJCRH Institutional Review Board approved the SJLIFE research (IRB no. SJLIFE), which broadly includes approval for analyses using data collected from SJLIFE participants. The study concept and analytic protocol is provided in the supplementary appendix (Supplementary Protocol S1). All participants provided written informed consent. Patients and the public were not involved in the design, conduct, reporting, or dissemination plans of this research. The study was conducted in accordance with the Declaration of Helsinki.

### Assessment of CHCs

A total of 177 clinically assessed CHCs were severity-graded using a modified version of the CTCAE v4.03.^16^ At baseline and each follow-up visit, CHCs were graded as 1 (mild), 2 (moderate), 3 (severe or disabling), or 4 (life-threatening), with the highest grade observed across visits retained. Following an established method,^17^ individual CHC grades at baseline and follow-ups were aggregated into a global CHC burden metric that summarizes overall morbidity by integrating the severity of all conditions into a single measure. Global burden was categorized as none/low (grades 0–1), medium (≥1 grade 2 and/or 1 grade 3), high (≥2 grade 3 or 1 grade 4 plus 1 grade 3), or very high burden (≥2 grade 4 or ≥2 grade 3 plus 1 grade 4). All 177 CHCs were categorized into 48 groups spanning 13 organ systems, with the highest grade across CHCs within each group used for analysis (Supplementary Table S1).^2^

To evaluate CHC severity progression, we assessed changes in global CHC burden and 48 CHC groups from baseline to follow-ups. By comparing global CHC burden categories at baseline and follow-up using an established method,^18^ survivors were classified as having progressive or non-progressive global CHC burden. To examine changes in individual CHC groups, survivors were classified into new-onset (CHC newly reaching a clinically significant grade ≥2), worsened (increasing from grade 2 or 3 to a higher grade), persistent (remaining at grade ≥2 without worsening), or stable low-grade (remaining at grades 0–1) for each CHC group (Supplementary Table S2). Due to fewer than 10 worsened cases in most groups, new-onset or worsened cases were combined to improve statistical stability. Four CHC groups with no or sparse outcome events (<5 cases) were excluded, leaving 44 CHC groups for analysis.

### Assessment of neighborhood SDOH

Participants' baseline residential addresses were geocoded to 11-digit Federal Information Processing Standards codes and linked to 2010 U.S. Census tract boundaries, geographic units within counties typically comprising 1200–8000 residents. Addresses recorded before 2010 were assigned to the corresponding 2010 Census tract boundaries to ensure a consistent geographic reference. Neighborhood SDOH measures were assigned using the baseline residential address.

We examined five Healthy People 2030 SDOH domains described in the Introduction to assess social and environmental/healthcare access vulnerabilities.^9^ Social vulnerability was measured using the U.S. Centers for Disease Control and Prevention’s Social Vulnerability Index (SVI), using 15 census-tract level indicators across four domains: socioeconomic status, household composition/disability, minority status/language, and housing/transportation (Supplementary Table S3). Environmental/healthcare access vulnerability was measured using five census-tract level indicators capturing healthcare accessibility and the built environment, including primary care and ambulatory care physician density (per 1000 residents); the proportion of uninsured residents; food inaccessibility (limited access to a supermarket or large grocery store); and poor walkability (based on land-use diversity, street connectivity, and commuting patterns; Supplementary Table S3). Because most baseline assessments occurred between 2009 and 2012 (median: 2011), social vulnerability was assessed using the 2014 SVI, derived from the 2010–2014 American Community Survey 5-year estimates. The remaining measures were selected from comparable reference periods. Domain- and indicator-specific scores were grouped using national quartiles (Q1: least vulnerable; Q4: most vulnerable) to facilitate comparisons with the U.S. population and across studies.

### Assessment of personal SES

Personal SES was assessed with self-reported health insurance (public, private, none) and educational attainment (high school or less, some college, university or above) items from the SJLIFE survey.

### Statistical analysis

Descriptive statistics summarized demographic variables (e.g., attained age, sex, race/ethnicity), clinical characteristics (e.g., cancer diagnosis, age at diagnosis, time since diagnosis, receipt of specific chemotherapy and radiotherapy),^15^ personal SES, and smoking status (never, past, current). Categorical variables were reported as frequencies and continuous variables as means and standard deviations (SD). Missing values in categorical variables were retained as separate categories to preserve the full analytic cohort and a consistent sample across models. Continuous variables had no missing data. Baseline characteristics of included and excluded survivors were compared using chi-square tests for categorical variables and independent two-sample t tests for continuous variables.

The prespecified primary analysis used multivariable logistic regression to estimate odds ratios (ORs) and 95% confidence intervals (CIs) for associations between SDOH quartiles (reference: Q1) and progression of global CHC burden (progression vs. non-progression [reference]). Because new-onset/worsened, persistent, and stable low-grade status represent distinct clinical trajectories without a natural ordering, multinomial logistic regression was used to evaluate associations between SDOH quartiles (reference: Q1) and changes in individual CHC groups (new-onset/worsened, persistent, and stable low-grade status [reference]). Models were adjusted for age at baseline, sex, cancer therapy (see specification below), time since diagnosis, time between baseline and follow-up when the most severe CHC was identified, personal SES (insurance coverage, educational attainment), and smoking status. For analyzing global CHC burden progression, models were adjusted for any chemotherapy and radiotherapy to account for treatment differences across all cancer types, given the heterogeneity in regimens and treatment sites. For analyzing changes in individual CHC groups, models incorporated individual chemotherapy agents and radiotherapy sites per the Children’s Oncology Group’s Long-Term Follow-Up Guidelines (Supplementary Table S4).^5^^,^^19^ Covariates were selected a priori based on survivorship literature and their relevance to CHC risks, including demographic, treatment-related, socioeconomic, and behavioral factors. Race and ethnicity were intentionally excluded as covariates from the primary analytic models. We conceptualized race and ethnicity as socially constructed markers of structural processes that contribute to social and residential conditions.^20^ Accordingly, we did not treat race and ethnicity as conventional confounders for associations between neighborhood SDOH and CHC outcomes.

To assess potential selection bias, we conducted a post-hoc sensitivity analysis using inverse probability weighting to account for selection into the analytic cohort. Inclusion probabilities were estimated using age at baseline, sex, any chemotherapy, any radiotherapy, time since diagnosis, insurance coverage, educational attainment, smoking status, baseline overall SVI, and baseline CHC burden, all of which were available for both included and excluded participants. Weighted models included the same covariate adjustment as the primary analysis. We also conducted a separate post-hoc sensitivity analysis in which the primary model was further adjusted for baseline global CHC burden category.

Joint effects of neighborhood SDOH and personal SES on global CHC burden progression and changes in individual CHC groups were prespecified and evaluated by estimating ORs for combinations of SDOH vulnerability (most [Q4] vs. less vulnerable [Q1–Q3]) and personal SES (e.g., public insurance vs. private/no insurance; high school diploma or less vs. higher education). Multiplicative interaction terms were used to assess effect modification, specifically whether associations between neighborhood SDOH and CHC outcomes differed by personal SES on the multiplicative scale. Our preliminary analyses showed no significant difference in global CHC burden progression between privately insured and uninsured survivors. Because uninsured individuals in the U.S. are heterogeneous with respect to income, employment status, and reasons for lacking insurance,^21^ and because preliminary analyses indicated similar risks, privately insured and uninsured survivors were combined as the reference group, with publicly insured survivors designated as the at-risk category. This approach reduced sparse cells and improved model stability. Models were adjusted for the same covariates stated above, except for the personal SES variable under evaluation.

Progression of global CHC burden was the prespecified primary outcome, whereas analyses of individual CHC groups and interactions with personal SES were considered exploratory and hypothesis-generating. Given the exploratory nature of these analyses and heterogeneity of SDOH measures and CHC outcomes evaluated independently, no formal adjustment for multiple comparisons was applied. A two-sided P < 0.05 was considered statistically significant for the primary analysis and served as a nominal significance threshold for the exploratory analyses. All analyses were conducted using SAS version 9.4 (SAS Inc., Cary, NC, USA).

### Role of the funding source

The funders had no role in the design, data collection, analysis, interpretation, manuscript preparation, or the decision to publish the manuscript.

## Results

Among participants, the mean age at cohort baseline was 31.6 years (SD: 8.4), and the mean time from initial diagnosis to baseline was 23.2 years (SD: 8.2; Table 1). Approximately 50% were male; 57% had a history of leukemia or lymphoma and 31% had solid tumors; 85% received chemotherapy and 58.9% underwent radiotherapy. Over 35% experienced progression of global CHC burden. Compared with excluded survivors, included survivors were older at baseline, had a longer interval from diagnosis to baseline, and were more likely to be female, non-Hispanic White, and to have received chemotherapy and radiotherapy (Supplementary Table S5).

**Table 1:** Characteristics of study participants.

	Survivors (n = 2508)
	n (%)
Sex	
Male	1258 (50.2)
Female	1250 (49.8)
Race and ethnicity	
Non-Hispanic White	2065 (82.3)
Non-Hispanic Black	346 (13.8)
Hispanic	64 (2.6)
Other	33 (1.3)
Age at baseline, years (mean ± SD)	31.6 ± 8.4
Time from diagnosis to SJLIFE baseline, years (mean ± SD)	23.2 ± 8.2
Time between baseline and follow-up (mean ± SD)	5.4 ± 1.9
Cancer diagnosis	
Leukemia	933 (37.2)
Lymphoma	490 (19.5)
Central nervous system tumors	282 (11.2)
Solid tumors	785 (31.3)
Other	18 (0.7)
Educational attainment	
≤ High school	697 (29.8)
Some college	821 (35.1)
University or above	824 (35.2)
Unknown	166
Health insurance coverage	
Private insurance	1612 (65.2)
Public insurance	328 (13.3)
No insurance	531 (21.5)
Unknown	37
Cigarette smoking status	
Never smoked	1702 (68.4)
Past smoker	335 (13.5)
Current smoker	453 (18.2)
Unknown	18
Chemotherapy	
Any chemotherapy	2131 (85.0)
Anthracyclines	1450 (57.8)
Alkylating agents	1482 (59.1)
Glucocorticoids	1186 (47.3)
Platinum agents	299 (11.9)
Epipodophyllotoxin	921 (36.7)
Methotrexate	684 (27.3)
Bleomycin	123 (4.9)
Vinca alkaloids	1807 (72.0)
Radiotherapy	
Any radiotherapy	1476 (58.9)
Brain	817 (32.6)
Chest	541 (21.6)
Abdomen	493 (19.7)
Pelvis	447 (17.8)
HSCT	140 (5.6)
Change in global CHC burden	
Non-progression	1625 (64.8)
Progression^a^	883 (35.2)
SVI overall	
Q1 (0.00–0.24)	513 (22.5)
Q2 (0.25–0.49)	584 (25.6)
Q3 (0.50–0.74)	710 (31.2)
Q4 (0.75–1.00)	472 (20.7)
Unknown	229
Fewer primary care physicians	
Q1 (≥0.66)	534 (23.4)
Q2 (0.17–0.65)	658 (28.9)
Q3 (0.01–0.16)	46 (2.0)
Q4 (0.00)	1041 (45.7)
Unknown	229
Limited ambulatory care physicians	
Q1 (≥4.39)	489 (21.5)
Q2 (1.96–4.38)	526 (23.1)
Q3 (0.87–1.95)	595 (26.1)
Q4 (<0.87)	669 (29.4)
Unknown	229
Uninsured population	
Q1 (<9.70%)	387 (17.0)
Q2 (9.70–13.99%)	622 (27.3)
Q3 (14.00–18.79%)	796 (34.9)
Q4 (≥18.80%)	474 (20.8)
Unknown	229
Food inaccessibility	
Q1 (<0.04)	240 (10.5)
Q2 (0.04–0.15)	373 (16.4)
Q3 (0.16–0.29)	705 (30.9)
Q4 (≥0.30)	961 (42.2)
Unknown	229
Poorer walkability	
Q1 (≥8.50)	135 (5.9)
Q2 (6.50–8.49)	417 (18.3)
Q3 (4.50–6.49)	814 (35.7)
Q4 (<4.50)	913 (40.1)
Unknown	229

CHC, chronic health condition; SD, standard deviation; SVI, Social Vulnerability Index; HSCT, hematopoietic stem cell transplantation.

a See Supplementary Table S2 for the definition of global CHC burden progression.

For social vulnerability assessed by the SVI (Fig. 1), survivors in neighborhoods with the most vs. least *overall and domain-specific* vulnerability (Q4 vs. Q1) had significantly higher risks of global CHC burden progression (OR: 1.44, 95% CI: 1.08–1.92 for overall; OR: 1.62, 95% CI: 1.22–2.15 for socioeconomic domain; OR: 1.65, 95% CI: 1.22–2.24 for household composition/disability domain). Specific *SVI indicators* capturing the most vs. least vulnerability (Q4 vs. Q1), including low income per capita (OR: 1.83, 95% CI: 1.36–2.46), low educational attainment (OR: 1.53, 95% CI: 1.15–2.04), higher proportions of younger population (OR: 1.50, 95% CI: 1.11–2.03), residents with disabilities (OR: 1.64, 95% CI: 1.23–2.19), and single-parent households (OR: 1.38, 95% CI: 1.03–1.84), were also associated with higher progression risks. For environmental/healthcare access vulnerability (Fig. 1), residing in the most vs. the least vulnerable neighborhoods (Q4 vs. Q1) was significantly associated with greater risks of CHC progression. Within the healthcare accessibility domain, indicators such as low access to primary care physicians (PCP; OR: 1.30, 95% CI: 1.03–1.64) and higher proportions of uninsured residents (OR: 1.36, 95% CI: 1.00–1.84) were associated with increased risks of CHC progression. Similarly, within the built environment domain, living in food-inaccessible neighborhoods (OR: 1.49, 95% CI: 1.08–2.07) was associated with higher progression risks.

**Fig. 1:**
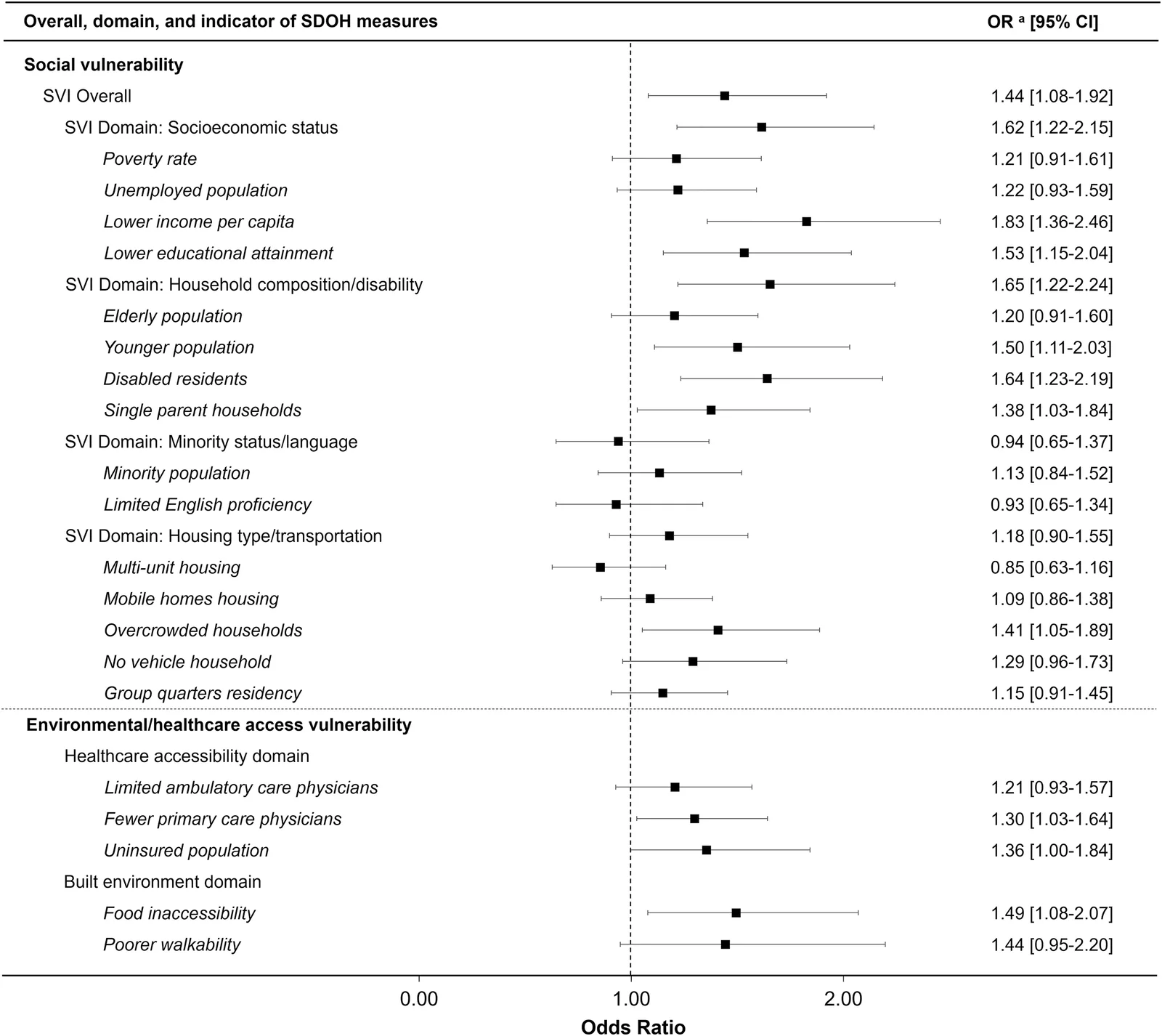
**Associations between neighborhood SDOH (Q4: most vulnerable vs. Q1: least vulnerable) and progression of global chronic health condition burden.** Squares indicate ORs and horizontal lines indicate 95% CIs. Italicized terms denote individual SDOH indicators. Abbreviations: CI, confidence interval; OR, odds ratio; SDOH, social determinants of health; SVI, Social Vulnerability Index. ^a^Adjusted for age at baseline, sex, time since diagnosis, time between baseline and follow-up, any chemotherapy, any radiotherapy, health insurance coverage, educational attainment, and smoking status.

In the sensitivity analysis using inverse probability weighting to account for potential selection bias, the overall pattern of results remained largely unchanged, with most findings consistent with the primary analysis. However, associations with overall SVI and uninsured population were no longer statistically significant, whereas the association with poor walkability became statistically significant (Supplementary Fig. S2). In a separate sensitivity analysis additionally adjusting for baseline global CHC burden, the results were largely unchanged, supporting the robustness of the primary findings (Supplementary Fig. S3).

Residing in the neighborhoods with the most vs. least *overall and domain-specific* social vulnerabilities (Q4 vs. Q1; Supplementary Fig. S4) was significantly associated with new-onset/worsening of seven CHC groups, including peripheral nervous system disorders (OR: 2.07, 95% CI: 1.30–3.28 for overall), abnormal glucose metabolism (OR: 1.93, 95% CI: 1.02–3.68 for household composition/disability domain), and asthma (OR: 2.70, 95% CI: 1.33–5.49 for minority status/language domain). At the *indicator level*, exploratory analyses identified 18 social and environmental/healthcare access vulnerability indicators associated with new-onset or worsening of 17 CHC groups, with nine CHC groups associated with at least two SDOH indicators (Fig. 2). These findings represented 43 significant associations among 880 comparisons conducted to evaluate new-onset or worsening CHC groups. For example, abnormal glucose metabolism showed associations with five indicators across neighborhood vulnerability (Q4 vs. Q1), including poor walkability (OR: 4.60, 95% CI: 1.41–15.04) and food inaccessibility (OR: 2.07, 95% CI: 1.01–4.27; Fig. 3). Peripheral nervous system disorders showed associations with six indicators, most notably higher proportions of residents with disabilities (OR: 3.52, 95% CI: 1.99–6.24) and low educational attainment (OR: 2.69, 95% CI: 1.63–4.43). Inflammatory disorders showed associations with four indicators, including food inaccessibility (OR: 2.53, 95% CI: 1.43–4.49) and poor walkability (OR: 2.50, 95% CI: 1.22–5.15). Arrhythmias showed associations with food inaccessibility (OR: 4.36, 95% CI: 1.32–14.35), low educational attainment (OR: 2.32, 95% CI: 1.07–5.04), and low access to ambulatory care physicians (OR: 2.14, 95% CI: 1.06–4.32).

**Fig. 2:**
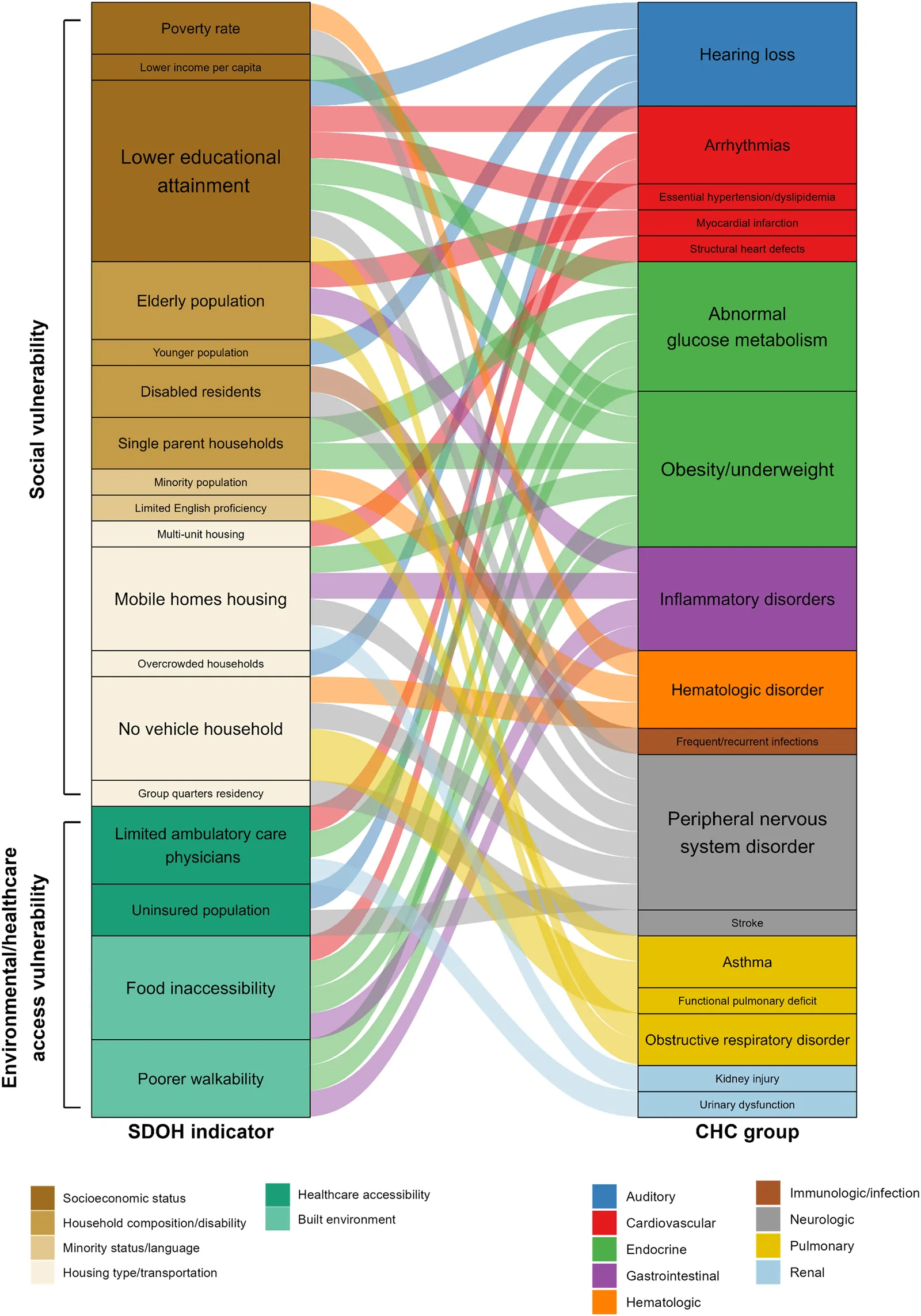
**Patterns of significant associations between multiple SDOH indicators and new-onset/worsening of CHC groups.** Abbreviations: CHC, chronic health condition; SDOH, social determinants of health.

**Fig. 3:**
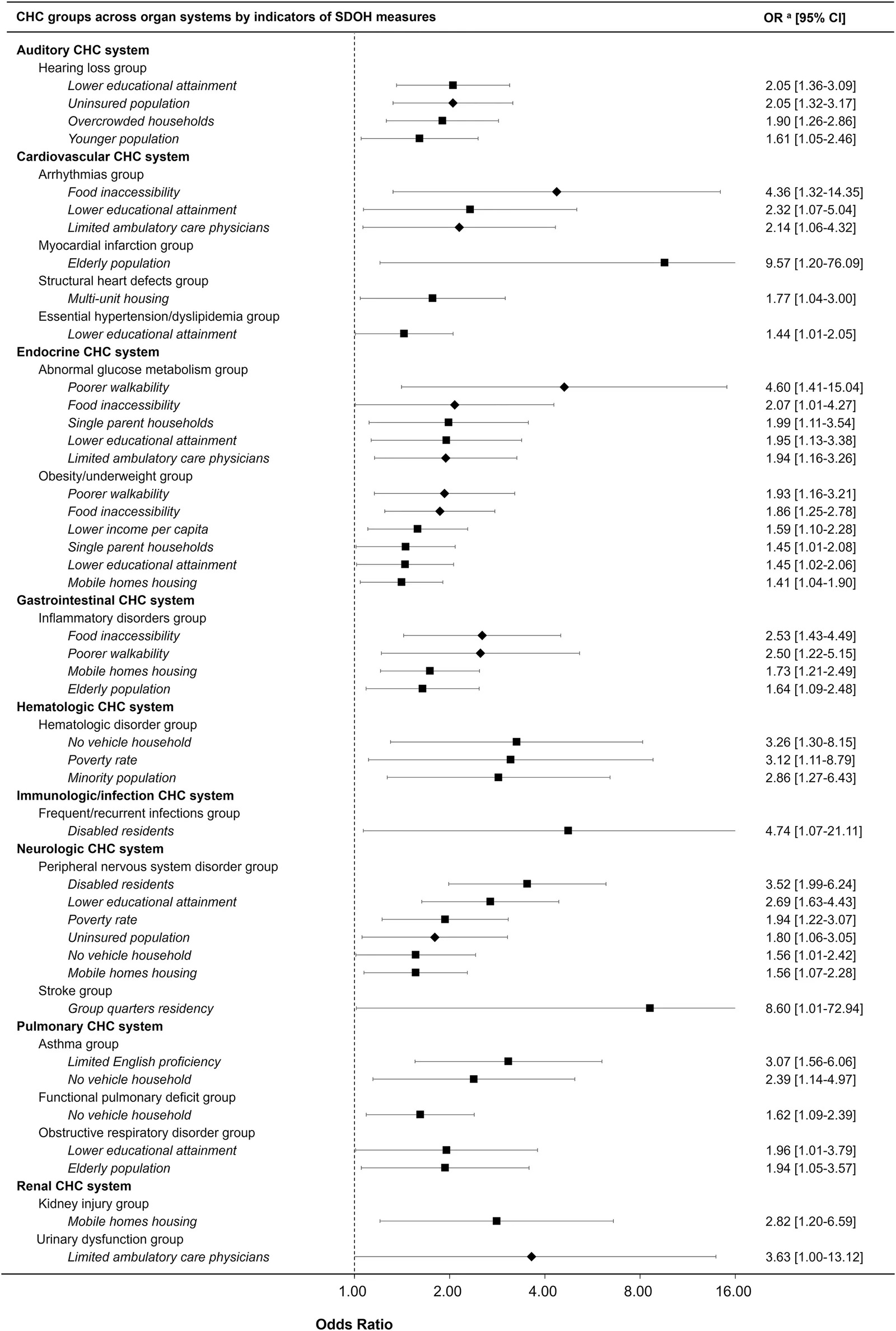
**Associations between neighborhood SDOH indicators (Q4: most vulnerable vs. Q1: least vulnerable) and new-onset/worsening of chronic health condition groups across organ systems.** This figure presents 43 associations that met the nominal significance threshold (two-sided P < 0.05) among 880 comparisons conducted for new-onset or worsening CHC groups. Odds ratios are shown as squares for social vulnerability and diamonds for environmental/healthcare access vulnerability, with horizontal lines indicating 95% CIs. Italicized terms denote individual SDOH indicators. Abbreviations: CHC, chronic health condition; CI, confidence interval; OR, odds ratio; SDOH, social determinants of health. ^a^Adjusted for age at baseline, sex, time since diagnosis, time between baseline and follow-up, organ-specific treatment for chemotherapy and radiotherapy (see Supplementary Table S4), health insurance coverage, educational attainment, and smoking status.

Several single SDOH indicators, rather than multi-indicator clustering, showed associations with new-onset/worsening CHC groups. For example, living in neighborhoods with higher proportions of elderly residents was associated with myocardial infarction (OR: 9.57, 95% CI: 1.20–76.09), and higher proportions of residents living in group quarters were associated with stroke (OR: 8.60, 95% CI: 1.01–72.94). Living in neighborhoods with higher proportions of residents with disabilities was associated with frequent or recurrent infections (OR: 4.74, 95% CI: 1.07–21.11), and low access to ambulatory care physicians was associated with urinary dysfunction (OR: 3.63, 95% CI: 1.00–13.12).

Persistent CHC status was significantly associated with residence in the most vs. least vulnerable neighborhoods (Q4 vs. Q1), as defined by *overall and domain-specific* SVI, across three CHC groups (Supplementary Fig. S4), including obesity or underweight (OR: 2.09, 95% CI: 1.49–2.94 for socioeconomic status domain) and central nervous system disorders (OR: 3.06, 95% CI: 1.04–9.01 for minority status/language domain). At the *indicator level*, exploratory analyses identified 15 social and environmental/healthcare access vulnerability indicators associated with persistent status across seven CHC groups, with four CHC groups associated with at least two SDOH indicators (Supplementary Fig. S5). These findings represented 23 significant associations among 880 comparisons conducted for persistent CHC groups. For example, persistent obesity or underweight was associated with ten indicators, including low income per capita (OR: 2.30, 95% CI: 1.62–3.27), higher proportions of uninsured population (OR: 1.81, 95% CI: 1.25–2.61), poor walkability (OR: 1.69, 95% CI: 1.08–2.65), and food inaccessibility (OR: 1.56, 95% CI: 1.09–2.25; Supplementary Fig. S6). Persistent essential hypertension or dyslipidemia was associated with four indicators, most notably low educational attainment (OR: 2.13, 95% CI: 1.41–3.22). Persistent cardiovascular dysfunction was associated with higher proportions of households without a vehicle (OR: 3.80, 95% CI: 1.42–10.15).

Survivors with public insurance coverage or a high school diploma or less who also resided in the most vulnerable neighborhoods (Q4) faced elevated risks of CHC progression than those with private or no insurance, higher educational attainment, and residence in less vulnerable neighborhoods (Q1–Q3). For global CHC burden progression, several significant joint associations were observed between neighborhood SDOH and personal SES (Supplementary Table S6). Strong joint effects were identified among survivors with public insurance (vs. private/no insurance) who resided in neighborhoods with limited access to PCPs (OR: 2.52, 95% CI: 1.75–3.64) and food inaccessibility (OR: 2.29, 95% CI: 1.59–3.31).

Across multiple organ systems, including cardiovascular, endocrine, gastrointestinal, musculoskeletal, neurological, ocular, and pulmonary systems, exploratory analyses identified statistically significant joint effects, supported by significant multiplicative interaction terms (Fig. 4). These findings represented 24 of 4400 interaction tests conducted. Outcome events and total participants in the at-risk and reference joint-exposure groups for each interaction analysis are presented in Supplementary Table S7. Among *new-onset/worsened* CHC groups, 16 significant joint effects were identified (Fig. 4A). Survivors with public insurance residing in neighborhoods with limited access to PCPs had higher risks of peripheral musculoskeletal disorders (OR: 6.11, 95% CI: 2.33–16.04) and ocular disorders (OR: 1.87, 95% CI: 1.21–2.87). Additionally, survivors with public insurance residing in neighborhoods with poor walkability had markedly elevated risks of peripheral musculoskeletal disorders (OR: 7.37, 95% CI: 2.82–19.27).

**Fig. 4:**
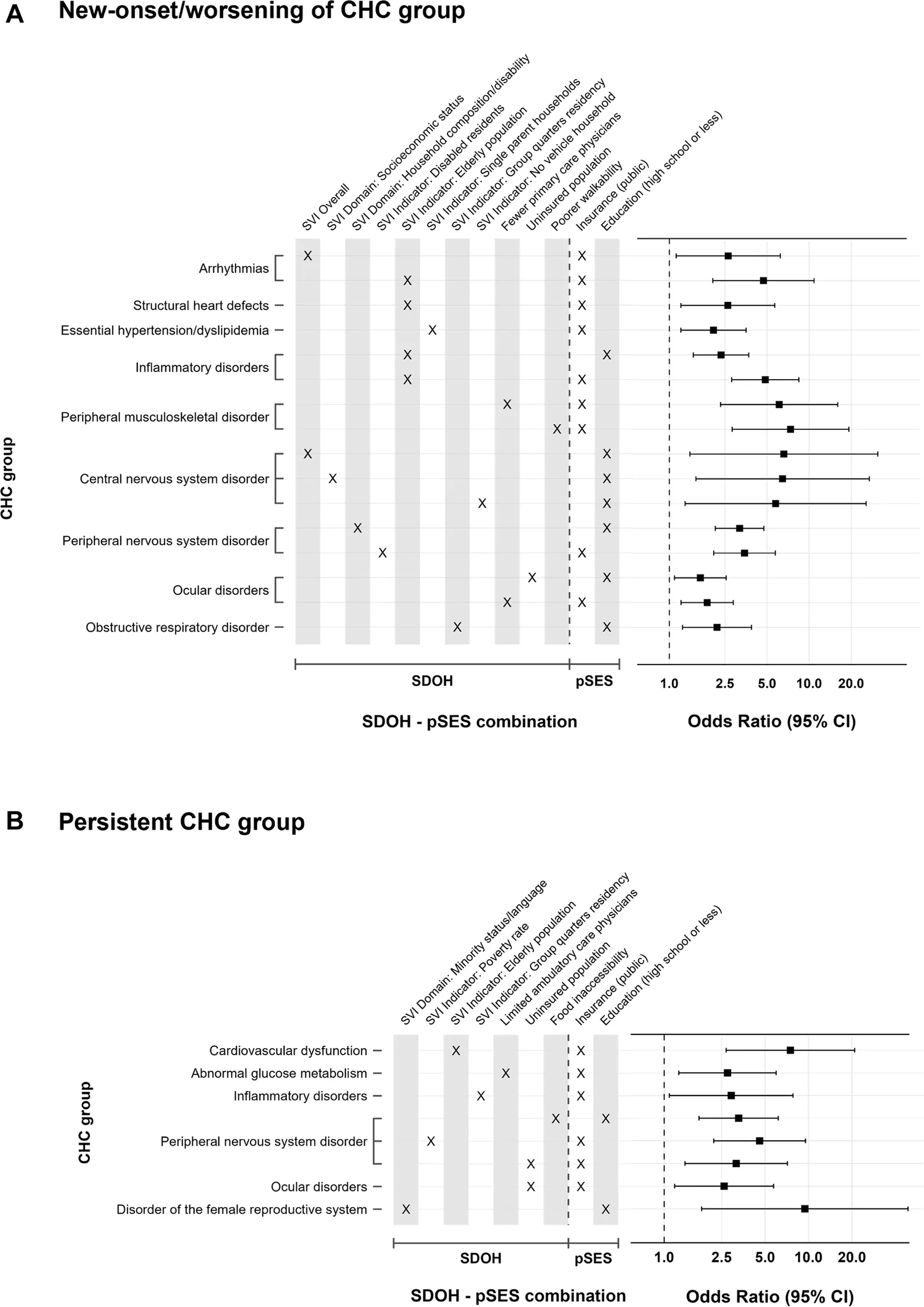
**Joint effects between neighborhood SDOH (Q4: most vulnerable vs. Q1–Q3: others) and personal socioeconomic status on the (A) new-onset/worsening and (B) persistent chronic health condition groups.** This figure presents 24 joint effects for which both the joint effect estimate and the corresponding multiplicative interaction term met the nominal significance threshold (two-sided P < 0.05) among 4400 interaction tests conducted. Odds ratios and 95% CI were calculated based on the combination of neighborhood SDOH vulnerability (Q4 vs. Q1–Q3) and personal socioeconomic status (e.g., high school diploma or less vs. higher education, public insurance vs. private or no insurance). Squares indicate ORs and horizontal lines indicate 95% CIs. Abbreviations: CHC, chronic health condition; CI, confidence interval; pSES, personal socioeconomic status; SDOH, social determinants of health; SVI, Social Vulnerability Index. ^a^Adjusted for age at baseline, sex, time since diagnosis, time between baseline and follow-up, organ-specific treatment for chemotherapy and radiotherapy (see Supplementary Table S4), and smoking status.

Among *persistent* CHC groups, eight significant joint effects with multiplicative interaction were observed (Fig. 4B). Survivors with public insurance residing in neighborhoods with low access to ambulatory care physicians had higher risks of abnormal glucose metabolism (OR: 2.75, 95% CI: 1.26–5.97). Similarly, survivors with public insurance residing in neighborhoods with higher proportions of uninsured residents had increased risks of peripheral nervous system disorders (OR: 3.16, 95% CI: 1.39–7.14) and ocular disorders (OR: 2.60, 95% CI: 1.18–5.73). Additionally, survivors with public insurance residing in neighborhoods with higher proportions of elderly residents had higher risks of persistence of cardiovascular dysfunction (OR: 7.49, 95% CI: 2.69–20.87).

## Discussion

These findings suggest that neighborhood disadvantages were associated with multimorbidity progression among childhood cancer survivors. Survivors in the most vs. the least vulnerable neighborhoods had approximately 2-fold higher risks of global CHC burden progression. Modifiable neighborhood conditions, particularly food inaccessibility and poor walkability, were associated with up to 5-fold higher risks for metabolic and cardiac outcomes and 10-fold higher risks for myocardial infarction and stroke. Notably, neighborhood vulnerability and personal SES acted synergistically, with survivors on public insurance in the most disadvantaged neighborhoods facing 7-fold higher risks of peripheral musculoskeletal and cardiovascular dysfunction, thereby identifying a clinically high-risk subgroup that warrants further investigation.

These findings further suggest that neighborhood conditions (e.g., poverty, healthcare accessibility, food environments) are associated with long-term health outcomes at a magnitude comparable to treatment exposures.^14^ Neighborhood context may therefore represent important, yet underrecognized, determinants of survivorship outcomes and warrants consideration alongside treatment history and genetic susceptibility in risk prediction, risk stratification, and care personalization.^2^^,^^22^ Incorporating neighborhood context into risk assessment could improve identification of high-risk survivors, a hypothesis that warrants evaluation in prospective trials. If confirmed, this approach could inform targeted, context-specific interventions. For example, survivors residing in food-inaccessible neighborhoods could be prioritized for preventive nutritional screening and linkage to hospital-based food support, SNAP enrollment, or community nutrition services to mitigate cardiac and metabolic risks.^23^

Importantly, this study demonstrates that neighborhood-level SDOH alone are insufficient to fully characterize survivors’ socioeconomic risk. Although area-level measures capture contextual disadvantage, they do not fully reflect individual-level barriers, such as limited education, lack of insurance coverage, housing instability, or transportation constraints, that independently influence health trajectories.^24^ Addressing these barriers requires an integrated risk assessment framework that combines geospatial SDOH measures with individual-level social risk screening.^25^ Given the interdisciplinary nature of survivorship care, psychologists and social workers can play critical roles in identifying social needs and connecting survivors with financial, housing, and community resources.^8^^,^^26^ However, these services are not routinely funded or available within long-term survivorship programs. In such settings, brief social-need screening (e.g., PRAPARE), paired with navigation or community health worker support, may offer a pragmatic approach,^25^ though its effectiveness in childhood cancer survivorship warrants prospective evaluation. Implementing these approaches requires health system infrastructure, including financial navigation, transportation support, and coordinated referral pathways across oncology, primary care, and community organizations (see Supplementary Table S8 for SDOH domain-specific strategies to address SDOH-related risk).

These findings also carry potential policy implications and warrant prospective evaluation of coordinated, multi-level strategies (i.e., community, state, federal) to address structural context underlying survivorship morbidity. The significant joint effects of personal public insurance enrollment and neighborhood disadvantage, particularly limited primary care access and food inaccessibility, on CHC burden highlight barriers that extend beyond individual-level clinical management and require system-level solutions. At the community level, expanding telehealth capacity and strengthening safety-net primary care could reduce geographic or transportation barriers to survivorship care.^27^ The associations between food inaccessibility and cardiometabolic and inflammatory outcomes suggest that integrating childhood cancer survivors into “Food is Medicine” initiatives and partnerships with local food resources warrants prospective evaluation.^28^ At the state and federal levels, embedding survivorship-specific provisions within Medicaid policy, the STAR Act, and state cancer control programs could expand coverage for nutrition support, transportation benefits, and telehealth reimbursement.^29^ Aligning policy with these risk profiles is essential to addressing structural inequities affecting this population.

Our findings identify priorities for equity-focused innovation in survivorship research and care for childhood cancer survivors. Although derived from a high-income country, these findings have broader global relevance, as SDOH-related disparities are experienced by cancer survivors across diverse settings and warrant attention within the global cancer survivorship agenda. Future work could refine extant risk prediction by integrating treatment exposures, clinical history, and granular neighborhood-level SDOH to enhance risk stratification and resource allocation.^30^ The observed associations also generate hypotheses on underlying mechanisms, including chronic stress, systemic inflammation, and social epigenomic processes.^22^ Prospective and pragmatic clinical trials are needed to evaluate scalable strategies, such as structured social risk screening, community health worker engagement, and technology-enabled navigation, to reduce SDOH-related health disparities.^26^ Together, these efforts suggest a more comprehensive approach to survivorship care across the life course.

Several limitations should be considered. The SJLIFE cohort comprises predominantly non-Hispanic White survivors treated at SJCRH, many of whom reside in the mid-South, which may limit generalizability to other racial/ethnic groups, geographic regions, and healthcare systems. In addition, the standardized long-term follow-up care at SJCRH may mitigate barriers associated with neighborhood disadvantage, potentially attenuating the observed associations. Nevertheless, the availability of detailed clinical and geospatial data, together with a broad SVI distribution, enabled evaluation across diverse neighborhood contexts. Second, neighborhood-level SDOH were measured only at baseline. Therefore, residential selection and reverse causation cannot be excluded, as survivors with greater disease burden or financial hardship may have moved to more disadvantaged neighborhoods before study entry. We were also unable to account for residential mobility or changes in neighborhood conditions during follow-up because repeat address data were available for approximately 10% of participants. However, our objective was to assess whether baseline neighborhood SDOH were associated with subsequent CHC progression to inform early risk stratification and targeted prevention. Future studies are needed to incorporate longitudinal SDOH trajectories and apply causal inference approaches to establish directionality. Third, community controls from SJLIFE were not included because the CHC data were limited to baseline. Our prior studies show that survivors in more socially vulnerable neighborhoods have worse quality-of-life (a proxy of greater CHC burden) than covariate-adjusted controls.^10^ Exploratory analyses also suggest significant joint effects of survivorship and neighborhood vulnerability on cross-sectional CHC burden (Supplementary Table S9). Fourth, although missingness was low for key categorical covariates, treating missing values as separate categories may introduce bias if missingness is associated with neighborhood SDOH, CHC outcomes, or other covariates. Several CHC-specific joint-effect and interaction analyses were based on few outcome events, resulting in wide confidence intervals and potentially unstable estimates. Therefore, these exploratory findings should be considered hypothesis-generating and require replication in independent survivor cohorts. Finally, SDOH measures used are U.S.-derived, which may limit direct generalizability; while similar geospatial data exist in the UK, Canada, Australia, and the EU, some local adaptation of measures and thresholds may be needed. Nonetheless, the overall framework may be transferable to settings with comparable SDOH data infrastructure. Future studies, particularly in low- and middle-income countries, should use locally relevant SDOH measures to improve external validity.

In conclusion, this large-scale, multi-level study demonstrates associations between neighborhood-level SDOH and long-term morbidity among childhood cancer survivors, independent of established treatment-related risks, and provides a testable foundation for evaluating whether modifying neighborhood conditions can improve outcomes. Survivors residing in highly vulnerable neighborhoods exhibited substantially elevated risks of CHC severity progression across multiple organ systems, with these associations generally stronger among survivors with personal socioeconomic disadvantages. These findings underscore the importance of integrating neighborhood context into survivorship risk stratification, care delivery, and policy planning. Advancing precision survivorship care will require coordinated efforts across clinical practice, community resources, and health policy to address structural vulnerabilities and improve long-term outcomes for this population.

## Contributors

ICH conceptualized the study. KKN and MMH acquired funding. JC and ICH developed the methodology. JC, SM, and DKS conducted the data analysis. All co-authors contributed to the interpretation of the data. ICH was responsible for project administration and supervision. KKN, GTA, MMH, and ICH provided resources. JC and ICH drafted the manuscript, and all co-authors reviewed and edited it. JC, SM, DKS, and ICH directly accessed and verified the underlying data reported in the manuscript. All authors read and approved the submitted version of the manuscript.

## Data sharing statement

The St. Jude Lifetime Cohort Study (SJLIFE) is funded by the U.S. National Cancer Institute (U01CA195547 and U01CA301480). SJLIFE data are publicly available on the St. Jude Survivorship Portal within the St. Jude Cloud at https://survivorship.stjude.cloud/.

## Declaration of interests

DKS reports consulting fees related to Department of Defense grant review. MJE reports support from Pfizer, NIH, and the Children’s Oncology Group Foundation. HMB reports NIH support and consulting fees from 1000 Feathers. KKN reports NIH and ALSAC support; GTA and ICH report NIH support; and MMH reports NCI support. All other authors declare no competing interests.

## Appendix A Supplementary data

Supplementary Figs. S1–S6 and Tables S1–S9

Supplementary Protocol 1
