# Supplementary material for: Neighborhood disadvantages and longitudinal progression of multimorbidity among adult survivors of childhood cancer: a single-center St. Jude Lifetime Cohort Study in the U.S
Source: eClinicalMedicine. 2026 Oct 13;101:104255. doi: 10.1016/j.eclinm.2026.104255 (PMC13685892; doi:10.1016/j.eclinm.2026.104255)
Supplement: Supplementary file 1 [file mmc1.pdf]

Neighborhood Disadvantages and Longitudinal Progression of Multimorbidity among Adult Survivors of Childhood Cancer: A Single-Center St. Jude Lifetime Cohort Study in the U.S.

Supplementary Table 1. 48 CHC groups organized by 13 organ systems (Page 2)

Supplementary Table 2. Classification of global CHC burden progression and changes in individual CHC groups over time (Page 4)

Supplementary Table 3. Content of the SDOH measures (Page 5)

Supplementary Table 4. Associations of chemotherapy and radiotherapy with risks of individual CHC organ systems (Page 7)

Supplementary Table 5. Comparison of baseline characteristics between included and excluded survivors (Page 8)

Supplementary Table 6. Joint effects between neighborhood SDOH and personal socioeconomic status, namely insurance and educational attainment, on progression of global chronic health condition burden (Page 9)

Supplementary Table 7. Event counts for the at-risk and reference groups in joint-effect analyses of neighborhood SDOH and personal socioeconomic status with CHC outcomes (Page 10)

Supplementary Table 8. Summary of significant associations between SDOHs and CHC outcomes in childhood cancer survivors and related policies or community programs for potential integration into survivorship care (Page 11)

Supplementary Table 9. Cross-sectional joint effects between neighborhood SDOH and survivorship status on prevalence of global CHC burden at baseline (Page 15)

Supplementary Figure 1. Flow diagram illustrating the selection of childhood cancer survivors for the study (Page 16)

Supplementary Figure 2. Inverse probability-of-selection weighted associations between neighborhood SDOH measures (Q4: most vulnerable vs. Q1: least vulnerable) and progression of global chronic health condition burden (Page 17)

Supplementary Figure 3. Sensitivity analysis for associations between neighborhood SDOH (Q4: most vulnerable vs. Q1: least vulnerable) and progression of global chronic health condition burden, additionally adjusting for baseline CHC burden (Page 19)

Supplementary Figure 4. Association between overall and domain-specific social vulnerability measures (Q4: most vulnerable vs. Q1: least vulnerable) and changes in chronic health condition groups (Page 21)

Supplementary Figure 5. Patterns of significant associations between multiple SDOH indicators and persistent CHC groups (Page 23)

Supplementary Figure 6. Associations between neighborhood SDOH indicators (Q4: most vulnerable vs. Q1: least vulnerable) and persistent chronic health condition groups across organ systems (Page 24)

Supplementary Table 1. 48 CHC groups organized by 13 organ systems

Organ system	CHC group	New-onset/ worsened (n)	Persistent (n)
Auditory	Hearing Loss	386	124
Cardiovascular	Myocardial infarction	31	8
	Arrhythmias	84	25
	Cardiovascular dysfunction	221	48
	Structural heart defects	156	16
	Vascular disease	29	2
	Essential hypertension/dyslipidemia	453	379
Endocrine	Growth hormone deficiency	35	17
	Obesity/underweight	732	1003
	Thyroid disorders	290	156
	Parathyroid disorders	7	0
	Abnormal glucose metabolism	169	93
	Conditions affecting the pituitary	30	18
Gastrointestinal	Esophageal disorders	50	19
	Disorders of the GI tract	40	17
	Inflammatory disorders	299	62
	Hepatic disorders	19	5
	Disorders of the gallbladder	10	0
Hematology	Hematologic disorder	45	7
Immunology/infections	Immunologic disorder	8	9
	Frequent/recurrent infections	39	4
	Chronic infection	21	42
	Severe infection [a]	1	0
Musculoskeletal	Amputation [a]	0	0
	Osteoporosis	222	151
	Joint disease	19	12
	Peripheral musculoskeletal disorder	33	2
	Spine disorder	86	13
Neurology	Stroke	16	0
	Central nervous system disorder	25	59

	Mixed nervous system disorder	32	25
	Peripheral nervous system disorder	278	132
	Seizures	47	58
	Severe headaches	184	45
Ocular	Ocular disorders	389	106
Pulmonary	Asthma	92	27
	Obstructive respiratory disorder	128	13
	Functional pulmonary deficit	356	179
	Respiratory parenchymal disease	10	0
Renal	Kidney injury	65	18
	Obstructive urinary disorder	16	4
	Urinary dysfunction	38	6
	Hematuria [a]	0	0
Sexual/reproductive	Disorder of the female reproductive system	56	18
	Disorder of the male reproductive system	33	9
	Male or female hypogonadism	160	172
	Conditions affecting the pituitary [a]	0	2
Neoplastic	Secondary and recurrent malignancies	40	1

Abbreviations: CHC, chronic health condition

Reference: Bhakta N, Liu Q, Ness KK*, et al.* The cumulative burden of surviving childhood cancer: an initial report from the St. Jude Lifetime Cohort Study (SJLIFE). Lancet 2017;390(10112):2569-2582.

[a] Among the 48 CHC groups, 4 were excluded from the analysis due to small sample size (<5) or absence of progression events

Supplementary Table 2. Classification of global CHC burden progression and changes in individual CHC groups over time

Types of CHC burden	Change in CHC burden from SJLIFE baseline to follow-up
Global CHC burden	Progression<br>• From none/low to high global burden<br>• From none/low to very high global burden<br>• From medium to high global burden<br>• From medium to very high global burden<br>• From high to high global burden<br>• From high to very high global burden<br>• From very high to very high global burden<br><br>Non-progression (otherwise)
Individual CHC groups	New-onset status [a]<br>• From CTCAE Grade 0 to 2<br>• From CTCAE Grade 0 to 3<br>• From CTCAE Grade 0 to 4<br>• From CTCAE Grade 1 to 2<br>• From CTCAE Grade 1 to 3<br>• From CTCAE Grade 1 to 4<br><br>Worsened status [a]<br>• From CTCAE Grade 2 to 3<br>• From CTCAE Grade 2 to 4<br>• From CTCAE Grade 3 to 4<br><br>Persistent status<br>• From CTCAE Grade 2 to 2<br>• From CTCAE Grade 3 to 3<br>• From CTCAE Grade 3 to 2<br>• From CTCAE Grade 4 to 4<br>• From CTCAE Grade 4 to 3<br>• From CTCAE Grade 4 to 2<br><br>Stable low-grade status (otherwise)

Abbreviations: CHC, chronic health condition

[a] New-onset and worsening status were merged in subsequent analysis.

Supplementary Table 3. Content of the SDOH measures

Domain	Indicator	Definition	Data source	Geo unit
Social vulnerability [a]				
Socioeconomic status	Lower educational attainment	Percentile of the % of persons with no high school diploma (age 25+) estimate	2014 SVI	Census tract
	Lower income per capita	Percentile of per capita income	2014 SVI	Census tract
	Poverty rate	Percentile of the % of persons below the poverty line	2014 SVI	Census tract
	Unemployed population	Percentile of the % of civilian (age 16+) unemployed estimate	2014 SVI	Census tract
Household composition/disability	Disabled residents	Percentile of the % of the civilian noninstitutionalized population with a disability	2014 SVI	Census tract
	Elderly population	Percentile of the % of persons aged 65 and older	2014 SVI	Census tract
	Single-parent households	Percentile of the % of single-parent households with children under 18	2014 SVI	Census tract
	Younger population	Percentile of the % of persons aged 17 and younger	2014 SVI	Census tract
Minority status/language	Limited English proficiency	Percentile of the % of persons (age 5+) who speak English "less than well"	2014 SVI	Census tract
	Minority population	Percentile of the % minority (all persons except white, non-Hispanic)	2014 SVI	Census tract
Housing/transportation	Overcrowded households	Percentile of the % households with more people than rooms	2014 SVI	Census tract
	Group quarters residency	Percentile of the % of persons in institutionalized and noninstitutionalized group quarters	2014 SVI	Census tract
	Mobile homes housing	Percentile of the % mobile homes	2014 SVI	Census tract
	Multi-unit housing	Percentile of the % housing in structures with 10 or more units	2014 SVI	Census tract
	No vehicle household	Percentile of the % households with no vehicle available	2014 SVI	Census tract
Environmental/healthcare access vulnerability				
Healthcare accessibility	Limited ambulatory care physicians	Ambulatory healthcare specialists per 1000 people	2010 NaNDA [b]	Census tract
	Fewer primary care physicians	Primary care physicians per 1,000 residents	2010 HRSA [c]	Census tract
	Uninsured population	% of uninsured civilian noninstitutionalized population	2008-2012 ACS	Census tract
Built environment	Food inaccessibility	% of the population with low income and low access within 1 mile	2010 USDA [d]	Census tract
	Poorer walkability	The national walkability index assesses occupied housing/mix of employment types/street intersection density/predicted commute mode	2013 EPA [e]	Census tract

Abbreviations: ACS, American Community Survey; EPA, United States Environmental Protection Agency; HRSA, Health Resources and Services Administration; NaNDA, National Neighborhood Data Archive; SDOH, social determinants of health; SVI, Social Vulnerability Index; USDA, United States Department of Agriculture

[a] Flanagan BE, Gregory EW, Hallisey EJ, Heitgerd JL, Lewis B. A social vulnerability index for disaster management. *Journal of homeland security and emergency management* 2011; **8**(1).

[b] Khan A, Li M, Finlay J. National Neighborhood Data Archive (NaNDA): health care services by census tract, United States 2003-2017. Open ICPSR.

[c] White KM, Zangaro G, Kepley HO, Camacho A. The Health Resources and Services Administration diversity data collection. *Public Health Rep* 2014; 129 Suppl 2(Suppl 2): 51-6.

[d] Rhone A, Ver Ploeg M, Dicken C, Williams R, Breneman V. Low-income and low-supermarket-access census tracts, 2010-2015. 2017.

[e] Thomas J, Zeller L. National walkability index user guide and methodology. *Environ Prot Agency: Washington, DC, USA* 2017.

Supplementary Table 4. Associations of chemotherapy and radiotherapy with risks of individual CHC organ systems

	Auditory	Cardiovascular	Endocrine	Gastrointestinal	Hematologic	Immunologic/ infection	Musculoskeletal	Neurologic	Ocular	Pulmonary	Renal	Sexual/ reproductive	Neoplastic
Chemotherapy													
Anthracyclines		✓			✓								
Alkylating agents			✓		✓							✓	✓
Glucocorticoids	✓	✓	✓				✓		✓				
Platinum agents	✓	✓						✓	✓		✓		
Epipodophyllotoxin					✓								✓
Methotrexate							✓				✓		
Bleomycin										✓			
Vinca alkaloids	✓							✓	✓				
Any chemotherapy	✓	✓	✓	✓	✓	✓	✓	✓	✓	✓	✓	✓	✓
Radiotherapy													
Brain	✓	✓	✓			✓	✓	✓	✓			✓	
Chest		✓	✓				✓			✓			✓
Abdomen		✓		✓			✓				✓	✓	✓
Pelvis			✓	✓								✓	
Any radiotherapy	✓	✓	✓	✓	✓	✓	✓	✓	✓	✓	✓	✓	✓

Abbreviations: CHC, chronic health condition

Reference:

Hudson MM, Ness KK, Gurney JG*, et al.* Clinical ascertainment of health outcomes among adults treated for childhood cancer. JAMA 2013;**309**(22):2371-2381.; Hudson MM, Bhatia S, Casillas J*, et al.* Long-term Follow-up Care for Childhood, Adolescent, and Young Adult Cancer Survivors. *Pediatrics* 2021;**148**(3):e2021053127.

Supplementary Table 5. Comparison of baseline characteristics between included and excluded survivors

**Characteristics**	**SJLIFE Survivors (n=4,686)**		**Excluded survivors (n=2,178)**		**Included survivors (n=2,508)**		
	**n**	**(%)**	**n**	**(%)**	**n**	**(%)**	**P-value[a]**
Sex							
Male	2,455	(52.4)	1197	(55.0)	1258	(50.2)	0.001
Female	2,231	(47.6)	981	(45.0)	1250	(49.8)	
Race and ethnicity							
Non-Hispanic White	3,726	(79.5)	1661	(76.3)	2065	(82.3)	<0.001
Non-Hispanic Black	730	(15.6)	384	(17.6)	346	(13.8)	
Hispanic	142	(3.0)	78	(3.6)	64	(2.6)	
Other	88	(1.9)	55	(2.5)	33	(1.3)	
Age at baseline, years (mean ± SD)	30.3 ± 8.6		28.6 ± 8.6		31.6 ± 8.4		<0.001
Time from diagnosis to SJLIFE baseline, years (mean ± SD)	21.5 ± 8.8		19.4 ± 8.9		23.2 ± 8.2		<0.001
Cancer diagnosis							
Leukemia	1,610	(34.4)	677	(31.1)	933	(37.2)	<0.001
Lymphoma	876	(18.7)	386	(17.7)	490	(19.5)	
Central nervous system tumors	661	(14.1)	379	(17.4)	282	(11.2)	
Solid tumors	1,497	(31.9)	712	(32.7)	785	(31.3)	
Other	42	(0.9)	24	(1.1)	18	(0.7)	
Educational attainment							
≤ High school	1,354	(32.3)	657	(35.4)	697	(29.8)	<0.001
Some college	1,487	(35.4)	666	(35.9)	821	(35.1)	
University or above	1,357	(32.3)	533	(28.7)	824	(35.2)	
Health insurance coverage							
Public insurance	697	(15.7)	369	(18.7)	328	(13.3)	<0.001
No insurance	893	(20.1)	362	(18.3)	531	(21.5)	
Private insurance	2,858	(64.3)	1246	(63.0)	1612	(65.2)	
Cigarette smoking status							
Never smoked	3,181	(70.6)	1479	(73.3)	1702	(68.4)	0.001
Past smoker	552	(12.2)	217	(10.8)	335	(13.5)	
Current smoker	775	(17.2)	322	(16.0)	453	(18.2)	
Chemotherapy							
Any chemotherapy	3,897	(83.2)	1766	(81.1)	2131	(85.0)	0.001
Radiotherapy							
Any radiotherapy	2,568	(54.8)	1092	(50.1)	1476	(58.9)	<0.001

[a] P-values were calculated by comparing included and excluded survivors using chi-square tests for categorical variables and independent two-sample t tests for continuous variables.

Supplementary Table 6. Joint effects between neighborhood SDOH and personal socioeconomic status, namely insurance and educational attainment, on progression of global chronic health condition burden

	**SDOH x Personal insurance**			**SDOH x Personal education**		
	**OR**	**(95% CI)**	$P_{interaction}$	**OR**	**(95% CI)**	$P_{interaction}$
Social vulnerability						
SVI Overall	2.14	(1.34-3.44)	0.764	1.57	(1.10-2.24)	0.475
SVI Domain: Socioeconomic status	2.14	(1.43-3.20)	0.681	1.59	(1.16-2.18)	0.804
Poverty rate	1.88	(1.23-2.88)	0.570	1.52	(1.08-2.14)	0.696
Unemployed population	1.71	(1.10-2.64)	0.303	1.39	(0.99-1.97)	0.961
Lower income per capita	2.41	(1.60-3.64)	0.953	1.69	(1.22-2.34)	0.990
Lower educational attainment	2.23	(1.46-3.40)	0.714	1.33	(0.97-1.83)	0.084
SVI Domain: Household composition/disability	2.08	(1.41-3.08)	0.411	1.43	(1.06-1.92)	0.415
Elderly population	2.28	(1.38-3.77)	0.887	1.76	(1.21-2.55)	0.145
Younger population	2.49	(1.53-4.06)	0.514	1.53	(1.06-2.20)	0.677
Disabled residents	2.23	(1.52-3.29)	0.574	1.59	(1.18-2.13)	0.850
Single parent households	1.58	(1.00-2.48)	0.064	1.35	(0.96-1.89)	0.284
SVI Domain: Minority status/language	1.38	(0.59-3.24)	0.364	1.49	(0.75-2.95)	0.543
Minority population	1.39	(0.83-2.34)	0.074	1.52	(1.00-2.32)	0.882
Limited English proficiency	1.26	(0.56-2.81)	0.316	1.07	(0.52-2.21)	0.698
SVI Domain: Housing type/transportation	1.86	(1.10-3.14)	0.883	1.26	(0.87-1.81)	0.879
Multi-unit housing	1.40	(0.67-2.95)	0.870	1.05	(0.58-1.90)	0.375
Mobile homes housing	2.54	(1.72-3.76)	0.161	1.29	(0.96-1.73)	0.191
Overcrowded households	2.29	(1.33-3.93)	0.834	1.53	(1.01-2.32)	0.470
No vehicle household	1.73	(1.01-2.95)	0.445	1.79	(1.18-2.71)	0.076
Group quarters residency	2.02	(1.23-3.30)	0.660	1.44	(1.03-2.03)	0.511
Environmental/healthcare access vulnerability						
Healthcare accessibility domain						
Limited ambulatory care physicians	2.26	(1.47-3.47)	0.304	1.15	(0.82-1.62)	0.053
Fewer primary care physicians	2.52	(1.75-3.64)	0.231	1.50	(1.13-2.00)	0.546
Uninsured population	2.37	(1.51-3.73)	0.547	1.57	(1.10-2.25)	0.373
Built environment domain						
Food inaccessibility	2.29	(1.59-3.31)	0.305	1.30	(0.97-1.74)	0.170
Poorer walkability	2.01	(1.36-2.97)	0.681	1.07	(0.79-1.46)	0.076

Abbreviations: CI, confidence interval; OR, odds ratio; SDOH, social determinants of health; SVI, Social Vulnerability Index

[a] ORs and 95%CI were calculated based on the combination of SDOH vulnerability (Q4 vs. Q1-Q3) and socioeconomic status (e.g., high school diploma or less vs. higher education, public insurance vs. private or no insurance), adjusted for age at baseline, sex, time since diagnosis, time between baseline and follow-up, any chemotherapy, any radiotherapy, and smoking status.

Supplementary Table 7. Event counts for the at-risk and reference groups in joint-effect analyses of neighborhood SDOH and personal socioeconomic status with CHC outcomes

CHC group	SDOH	Personal SES	OR [a]	(95%CI)	$P_{interaction}$	At-risk group [b] (events/total)	Reference group [c] (events/total)
**Onset/worsening status**							
Arrhythmias	SVI Overall	Insurance (public)	2.64	(1.12-6.23)	0.027	7/89	56/1581
Arrhythmias	SVI Indicator: Elderly population	Insurance (public)	4.71	(2.05-10.85)	<0.001	8/74	46/1502
Structural heart defects	SVI Indicator: Elderly population	Insurance (public)	2.62	(1.21-5.68)	0.015	9/74	94/1502
Essential hypertension/dyslipidemia	SVI Indicator: Single-parent households	Insurance (public)	2.07	(1.21-3.54)	0.008	23/97	287/1522
Inflammatory disorders	SVI Indicator: Elderly population	Education (high school or less)	2.34	(1.49-3.70)	<0.001	32/151	133/1146
Inflammatory disorders	SVI Indicator: Elderly population	Insurance (public)	4.86	(2.79-8.45)	<0.001	23/74	158/1502
Peripheral musculoskeletal disorder	Fewer primary care physicians	Insurance (public)	6.11	(2.33-16.04)	<0.001	9/152	10/1080
Peripheral musculoskeletal disorder	Poorer walkability	Insurance (public)	7.37	(2.82-19.27)	<0.001	9/128	10/1181
Central nervous system disorder	SVI Overall	Education (high school or less)	6.60	(1.40-31.02)	0.017	3/174	4/1235
Central nervous system disorder	SVI Domain: Socioeconomic status	Education (high school or less)	6.46	(1.55-26.96)	0.011	4/230	4/1188
Central nervous system disorder	SVI Indicator: No vehicle household	Education (high school or less)	5.76	(1.30-25.58)	0.021	3/118	5/1301
Peripheral nervous system disorder	SVI Domain: Household composition/disability	Education (high school or less)	3.18	(2.13-4.75)	<0.001	54/284	92/1049
Peripheral nervous system disorder	SVI Indicator: Disabled residents	Insurance (public)	3.45	(2.08-5.73)	<0.001	27/135	99/1288
Ocular disorders	Uninsured population	Education (high school or less)	1.67	(1.09-2.55)	0.019	36/164	202/1230
Ocular disorders	Fewer primary care physicians	Insurance (public)	1.87	(1.21-2.87)	0.004	38/152	166/1080
Obstructive respiratory disorder	SVI Indicator: Group quarters residency	Education (high school or less)	2.19	(1.24-3.87)	0.007	19/201	59/1106
**Persistent status**							
Cardiovascular dysfunction	SVI Indicator: Elderly population	Insurance (public)	7.49	(2.69-20.87)	<0.001	6/74	25/1502
Abnormal glucose metabolism	Limited ambulatory care physicians	Insurance (public)	2.75	(1.26-5.97)	0.011	9/109	53/1408
Inflammatory disorders	SVI Indicator: Group quarters residency	Insurance (public)	2.91	(1.09-7.81)	0.034	5/80	41/1421
Peripheral nervous system disorder	Food inaccessibility	Education (high school or less)	3.28	(1.74-6.17)	<0.001	20/311	24/912
Peripheral nervous system disorder	SVI Indicator: Poverty rate	Insurance (public)	4.59	(2.21-9.53)	<0.001	11/108	45/1512
Peripheral nervous system disorder	Uninsured population	Insurance (public)	3.16	(1.39-7.14)	0.006	8/94	49/1586
Ocular disorders	Uninsured population	Insurance (public)	2.60	(1.18-5.73)	0.018	9/94	66/1586
Disorder of the female reproductive system	SVI Domain: Minority status/language	Education (high school or less)	9.43	(1.82-48.90)	0.008	2/44	12/1388

[a] Adjusted for age at baseline, sex, time since diagnosis, time between baseline and follow-up, organ-specific treatment for chemotherapy and radiotherapy (see Supplementary Table 4), and smoking status.

[b] The at-risk group was defined as Q4 neighborhood SDOH combined with the disadvantaged pSES category, defined as public insurance or a high school education or less. Values are shown as outcome events/total survivors in the joint-exposure group.

[c] The Reference group was defined as Q1-Q3 neighborhood SDOH combined with the reference pSES category, defined as private or no insurance or education beyond high school. Values are shown as outcome events/total survivors in the joint-exposure group.

Supplementary Table 8. Summary of significant associations between SDOHs and CHC outcomes in childhood cancer survivors and related policies or community programs for potential integration into survivorship care

SDOH indicator	Associated CHC (global burden or CHC group)	Potential issues in adult survivors of childhood cancer	Coping strategy	Example of policy, community program, and intervention	Reference (see footnote for full information)
Social vulnerability					
Socioeconomic status domain					
Lower educational attainment	• Global CHC burden<br>• CHC groups<br>○ New-onset/worsening<br>- Hearing loss<br>- Arrhythmias<br>- Essential hypertension/dyslipidemia<br>- Abnormal glucose metabolism<br>- Obesity/underweight<br>- Peripheral nervous system disorder<br>- Obstructive respiratory disorder<br>○ Persistent<br>- Essential hypertension/ dyslipidemia<br>- Abnormal glucose metabolism<br>- Obesity/underweight	Economic hardship in the surrounding community can create unstable work schedules, limited job opportunities, and financial strain. These structural pressures often interfere with a survivor's ability to attend health care appointments, sustain health-promoting behaviors, obtain medication, and maintain long-term management of chronic health conditions. Living in neighborhoods with lower educational attainment may limit access to clear, accurate, and culturally appropriate health information, which can hinder survivors' understanding of follow-up care needs and long-term condition management.	Identify financial and employment-related barriers early and provide support to help survivors manage medical expenses, transportation costs, and insurance challenges. When survivors face unstable employment, connect them to job training or workforce development programs that can strengthen income stability and reduce interruptions in care. Provide survivors with clear, tailored health communication and navigation support to help them understand and act on care plans when living in neighborhoods with lower educational attainment.	- Financial navigation services integrated into oncology and survivorship care<br>- Vocational rehabilitation and job training programs offered through community organizations or state employment services<br>- Health-literacy–focused education initiatives that reinforce understanding of survivorship care plans.	Offodile et al. 2022<br>Algeo et al. 2021<br>Maheu et al. 2025<br>Duggan et al. 2023
Lower income per capita	• Global CHC burden<br>• CHC groups<br>○ New-onset/worsening<br>- Obesity/underweight<br>○ Persistent<br>- Obesity/underweight				
Poverty rate	• CHC groups<br>○ New-onset/worsening<br>- Hematologic disorder<br>- Peripheral nervous system disorder<br>○ Persistent<br>- Essential hypertension/dyslipidemia<br>- Obesity/underweight<br>- Disorder of the female reproductive system				
Unemployed population	• CHC groups<br>○ Persistent<br>- Obesity/underweight<br>- Disorder of the female reproductive system				
Household composition/disability domain					
Disabled residents	• Global CHC burden<br>• CHC groups<br>○ New-onset/worsening<br>- Frequent/recurrent infections<br>- Peripheral nervous system disorder<br>○ Persistent<br>- Essential hypertension/dyslipidemia<br>- Obesity/underweight	High caregiving responsibilities and resource-constrained local systems can make it difficult for survivors to schedule and maintain their own follow-up, as their personal care needs often compete	Assess whether caregiving or household responsibilities interfere with appointments and offer coordination support to help organize schedules and reduce competing demands so survivor care stays on track.	- Caregiver support services that ease care burden<br>- Care coordination<br>- Community health worker navigation	Choi et al. 2024<br>Peikes et al. 2009<br>Kangovi et al. 2014

Elderly population	• CHC groups<br>o New-onset/worsening<br>- Myocardial infarction<br>- Inflammatory disorders<br>- Obstructive respiratory disorder<br>o Persistent<br>- Cardiovascular dysfunction	with other household or community responsibilities.			
Single-parent households	• Global CHC burden<br>• CHC groups<br>o New-onset/worsening<br>- Abnormal glucose metabolism<br>- Obesity/underweight<br>o Persistent<br>- Obesity/underweight				
Younger population	• Global CHC burden<br>• CHC groups<br>o New-onset/worsening<br>- Hearing loss				
Minority status/language domain					
Limited English proficiency	• CHC groups<br>o New-onset/worsening<br>- Asthma<br>o Persistent<br>- Central nervous system disorder<br>- Disorder of the female reproductive system	When medical information does not align with a survivor's language or cultural context, communication gaps can occur. This can reduce understanding of survivorship plans and weaken engagement in long-term follow-up. This is particularly salient for women.	Provide communication support that helps survivors fully understand care plans and feel comfortable asking questions. Interpreter services or preferred language navigation can strengthen comprehension and improve follow-up	- Medicaid reimbursement for interpreter services in primary care settings<br>- Community health worker programs	Heath et al. 2023<br>Kangovi et al. 2014
Minority population	• CHC groups<br>o New-onset/worsening<br>- Hematologic disorder<br>o Persistent<br>- Esophageal disorders<br>- Disorder of the female reproductive system				
Housing type/transportation domain					
Overcrowded households	• Global CHC burden<br>• CHC groups<br>o New-onset/worsening<br>- Hearing loss	Living in areas with housing and transportation barriers includes issues of unstable housing, lack of private space for telehealth visits, and difficulty securing reliable transportation to clinics. All of these can interfere with scheduling appointments and continuity of survivorship follow-up.	Identify stable housing and reliable transportation and offer support that improves a survivor's ability to reach follow-up care, including coordinated scheduling or travel assistance when needed.	- Medicare-supported non-emergency medical transportation and pilot rideshare programs	Chaiyachati et al. 2018
Group quarters residency	• CHC groups<br>o New-onset/worsening<br>- Stroke<br>o Persistent<br>- Essential hypertension/dyslipidemia				
Mobile home housing	• CHC groups<br>o New-onset/worsening<br>- Obesity/underweight<br>- Inflammatory disorders<br>- Peripheral nervous system disorder<br>- Kidney injury<br>o Persistent<br>- Obesity/underweight				

Multi-unit housing	• CHC groups<br>○ New-onset/worsening<br>- Structural heart defects				
No vehicle household	• CHC groups<br>○ New-onset/worsening<br>- Hematologic disorder<br>- Peripheral nervous system disorder<br>- Asthma<br>- Functional pulmonary deficit<br>○ Persistent<br>- Cardiovascular dysfunction				
Environmental/healthcare access vulnerability					
Healthcare accessibility domain					
Limited ambulatory care physicians	• CHC groups<br>○ New-onset/worsening<br>- Arrhythmias<br>- Abnormal glucose metabolism<br>- Urinary dysfunction	In areas with limited primary care and unstable insurance coverage, survivors may lack a reliable medical home to manage ongoing chronic health conditions after completing oncology care. This may be particularly important for survivors with conditions that require consistent monitoring (e.g., arrhythmias, abnormal glucose metabolism).	Support survivors in establishing stable primary care, securing consistent insurance coverage, and accessing care through flexible and reliable delivery routes when in-person options are difficult to reach.	- Federally qualified health centers<br>- Telehealth supported by parity laws<br>- Care coordination<br>- Community health worker program	Erikson et al. 2022<br>Peikes et al. 2009<br>Kangovi et al. 2014
Fewer primary care physicians	• Global CHC burden				
Uninsured population	• Global CHC burden<br>• CHC groups<br>○ New-onset/worsening<br>- Hearing loss<br>- Peripheral nervous system disorder<br>○ Persistent<br>- Obesity/underweight				
Built environment domain					
Food inaccessibility	• Global CHC burden<br>• CHC groups<br>○ New-onset/worsening<br>- Arrhythmias<br>- Abnormal glucose metabolism<br>- Obesity/underweight<br>- Inflammatory disorders<br>○ Persistent<br>- Obesity/underweight	Limited access to healthy food and safe activity spaces makes it difficult for survivors to follow recommended diet and physical activity routines, increasing risk for metabolic conditions such as obesity and abnormal glucose metabolism.	Identify nutrition or activity barriers and connect survivors to programs that improve access to healthy foods and practical ways to maintain metabolic health.	- Hospital-based food support<br>- Medically Tailored Meals (MTM) by Medicaid<br>- 'Food as Medicine' initiative by the U.S. Department of Health and Human Services<br>- 'Exercise is Medicine' Initiative by the American College of Sports Medicine	Berkowitz et al. 2019<br>Clark et al. 2025<br>Volpp et al. 2023<br>Thompson et al. 2020
Poorer walkability	• CHC groups<br>○ New-onset/worsening<br>- Abnormal glucose metabolism<br>- Obesity/underweight<br>- Inflammatory disorders<br>○ Persistent<br>- Obesity/underweight				

Abbreviations: CHC, Chronic health condition; SDOH, social determinants of health

References:

- Algeo N, Bennett K, Connolly D. Rehabilitation interventions to support return to work for women with breast cancer: A systematic review and meta-analysis. *BMC Cancer* 2021; **21**(1): 895.
- Berkowitz SA, Terranova J, Randall L, Cranston K, Waters DB, Hsu J. Association between receipt of a medically tailored meal program and health care use. *JAMA Intern Med* 2019; **179**(6): 786-93.
- Chaiyachati KH, Hubbard RA, Yeager A, et al. Association of rideshare-based transportation services and missed primary care appointments: A clinical trial. *JAMA Intern Med* 2018; **178**(3): 383-9.
- Choi H, Reblin M, Litzelman K. Conceptualizing family caregivers' use of community support services: A scoping review. *Gerontologist* 2024; **64**(5).
- Clark JM, Maw MTT, Pettway K, et al. Impact of medically tailored meals on clinical outcomes among low-income adults with type 2 diabetes: A pilot randomized trial. *J Gen Intern Med* 2025; **40**(8): 1711-9.
- Duggan C, Cushing-Haugen KL, Cole AM, et al. Feasibility of delivering survivorship care via lay health educators a pilot randomized controlled trial among rural cancer survivors. *J Rural Health* 2023; **39**(3):666-675.
- Erikson C, Herring J, Park YH, Luo Q, Burke G. Association between state payment parity policies and telehealth usage at community health centers during COVID-19. *J Am Med Inform Assoc* 2022; **29**(10): 1715-21.
- Heath M, Hvass AMF, Wejse CM. Interpreter services and effect on healthcare - a systematic review of the impact of different types of interpreters on patient outcome. *J Migr Health* 2023; **7**: 100162.
- Kangovi S, Mitra N, Grande D, et al. Patient-centered community health worker intervention to improve posthospital outcomes: A randomized clinical trial. *JAMA Intern Med* 2014; **174**(4): 535-43.
- Maheu C, Parkinson M, Johnson K, et al. Pilot randomized controlled trial of iCanWork: Theory-guided return-to-work intervention for individuals touched by cancer. *Curr Oncol* 2025; **32**(5).
- Offodile AC, Gallagher K, Angove R, Tucker-Seeley RD, Balch A, Shankaran V. Financial navigation in cancer care delivery: State of the evidence, opportunities for research, and future directions. *J Clin Oncol* 2022; **40**(21): 2291-4.
- Peikes D, Chen A, Schore J, Brown R. Effects of care coordination on hospitalization, quality of care, and health care expenditures among Medicare beneficiaries: 15 randomized trials. *JAMA* 2009; **301**(6): 603-18.
- Thompson WR, Sallis R, Joy E, Jaworski CA, Stuhr RM, Trilk JL. Exercise is medicine. *Am J Lifestyle Med* 2020; **14**(5): 511-23.
- Volpp KG, Berkowitz SA, Sharma SV, et al. Food is medicine: A presidential advisory from the American Heart Association. *Circulation* 2023; **148**(18): 1417-39.

Supplementary Table 9. Cross-sectional joint effects between neighborhood SDOH and survivorship status on prevalence of global CHC burden at baseline

	Joint effect between SDOH (Q4 vs. Q1-Q3) and survivorship status (survivor vs. control [a]) on global CHC burden	
	**OR [b]**	**(95% CI)**
Social vulnerability		
SVI Overall	3.21	(2.32-4.44)
SVI Domain: Socioeconomic status	2.96	(2.15-4.06)
Poverty rate	2.95	(2.15-4.06)
Unemployed population	2.89	(2.11-3.98)
Lower income per capita	3.21	(2.34-4.40)
Lower educational attainment	2.79	(2.02-3.86)
SVI Domain: Household composition/disability	3.20	(2.32-4.42)
Elderly population	2.82	(2.05-3.89)
Younger population	2.36	(1.72-3.25)
Disabled residents	3.37	(2.42-4.70)
Single parent households	3.09	(2.23-4.27)
SVI Domain: Minority status/language	2.68	(1.77-4.05)
Minority population	3.32	(2.36-4.67)
Limited English proficiency	3.04	(2.03-4.54)
SVI Domain: Housing type/transportation	2.80	(2.02-3.88)
Multi-unit housing	2.09	(1.46-3.00)
Mobile homes housing	4.46	(3.10-6.41)
Overcrowded households	3.18	(2.28-4.44)
No vehicle household	3.26	(2.32-4.57)
Group quarters residency	3.16	(2.29-4.38)
Environmental/healthcare access vulnerability		
Healthcare accessibility domain		
Limited ambulatory care physicians	2.24	(1.63-3.07)
Fewer primary care physicians	2.57	(1.87-3.52)
Uninsured population	3.26	(2.36-4.51)
Built environment domain		
Food inaccessibility	3.99	(2.83-5.63)
Poorer walkability	3.06	(2.18-4.28)

Abbreviations: CHC, chronic health condition; CI, confidence interval; OR, odds ratio; SDOH, social determinants of health; SVI, Social Vulnerability Index

[a] Characteristics of the control group at baseline: mean age 33.2 years (SD: 10.3), sex (44.2% male; 55.8% female), educational attainment (14.5% ≤high school; 29.5% some college; 55.9% university or above), health insurance coverage (83.2% private insurance; 5.2% public insurance; 11.6% no insurance), smoking status (72.8% never smoked; 15.8% past smoker; 11.4% current smoker)

[b] ORs and 95%CI were calculated based on the combination of SDOH vulnerability (Q4 vs. Q1-Q3) and survivorship status (survivor vs. control), adjusted for age at baseline, sex, educational attainment, health insurance coverage, and smoking status.

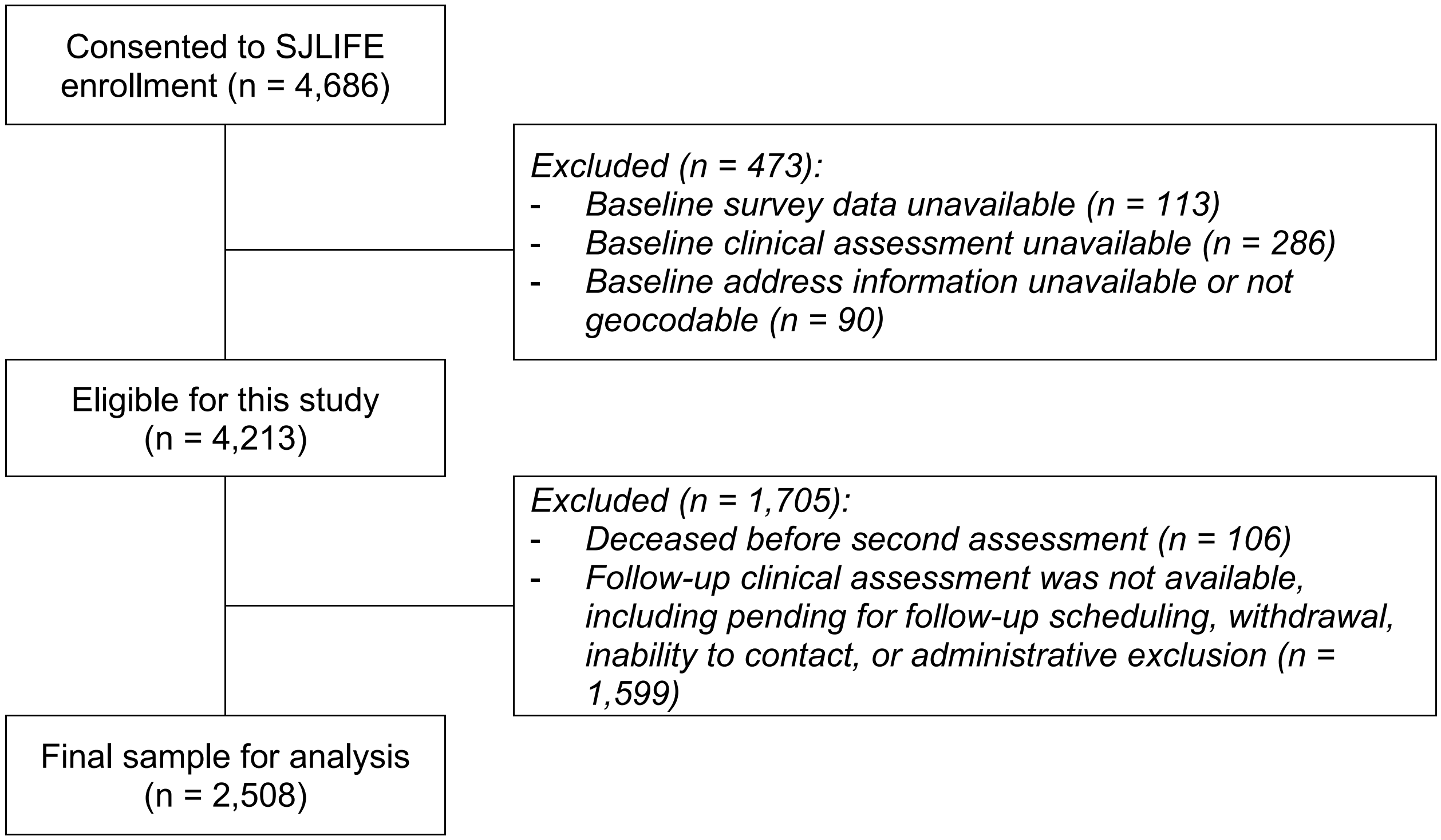

Supplementary Figure 1. Flow diagram illustrating the selection of childhood cancer survivors for the study

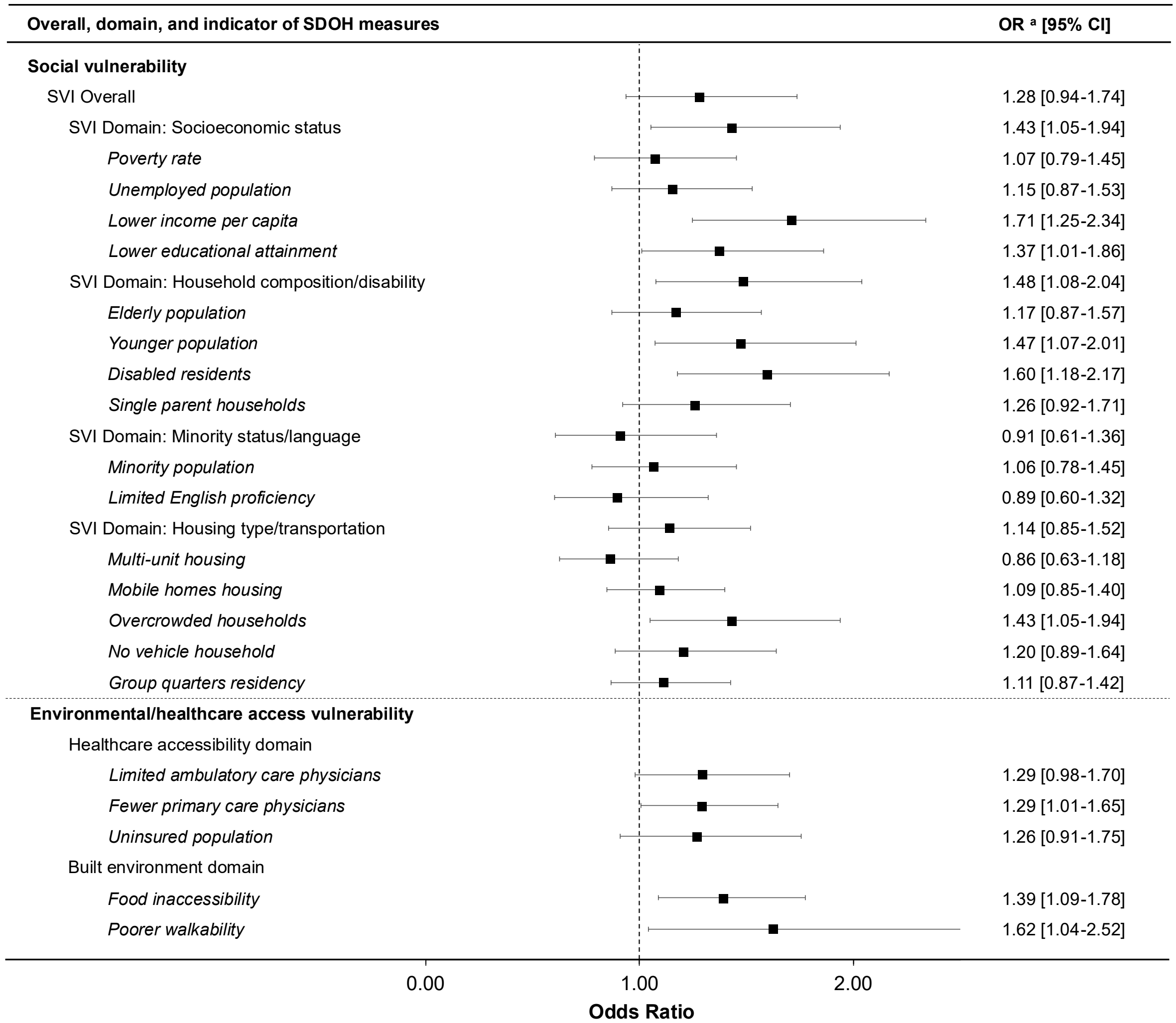

Supplementary Figure 2. Inverse probability-of-selection weighted associations between neighborhood SDOH measures (Q4: most vulnerable vs. Q1: least vulnerable) and progression of global chronic health condition burden

Squares indicate ORs and horizontal lines indicate 95%CIs. Italicized terms denote individual SDOH indicators.

Abbreviations: CI, confidence interval; OR, odds ratio; SDOH, social determinants of health; SVI, Social Vulnerability Index

[a] Adjusted for age at baseline, sex, time since diagnosis, time between baseline and follow-up, any chemotherapy, any radiotherapy, health insurance coverage, educational attainment, and smoking status.

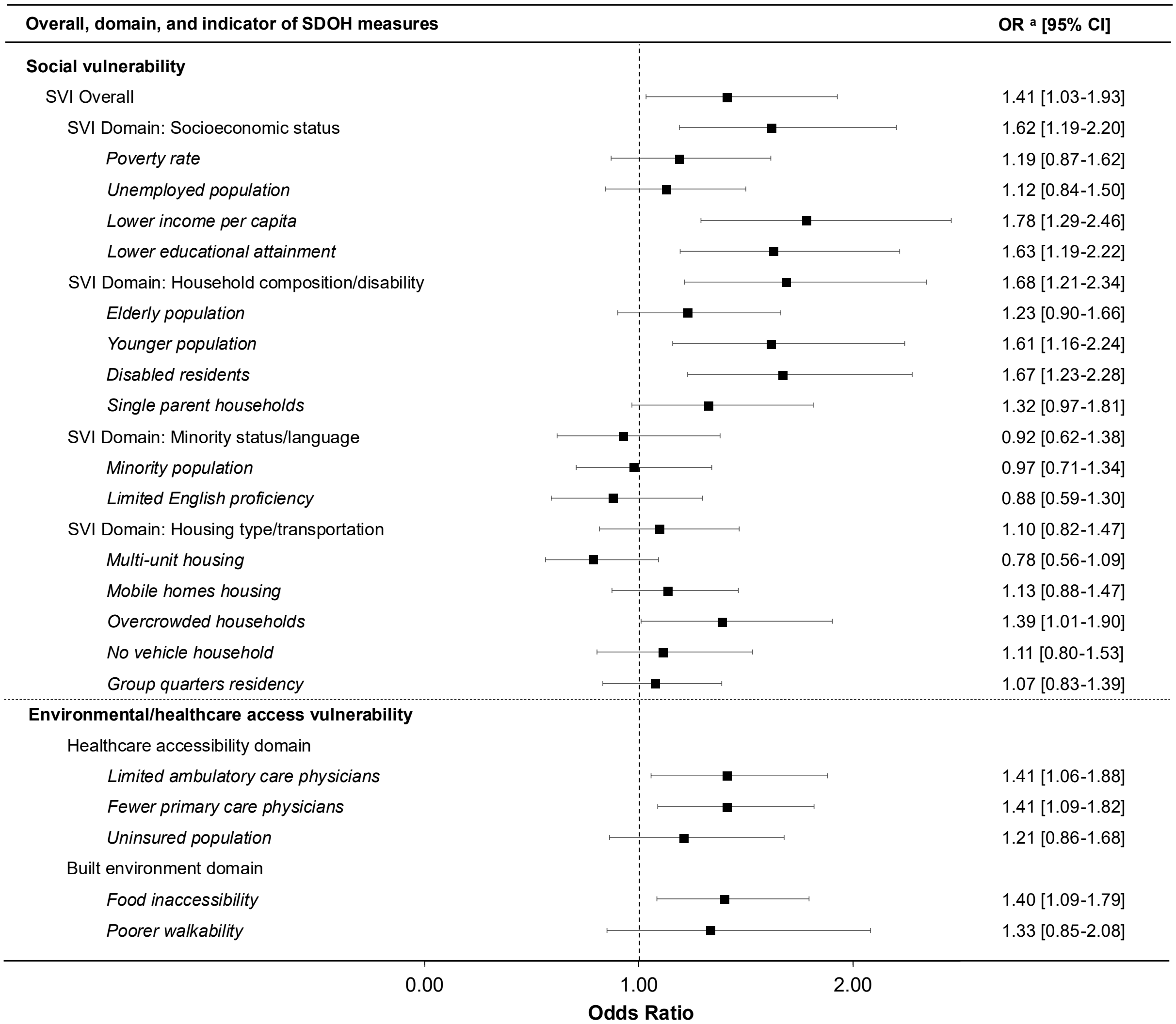

Supplementary Figure 3. Sensitivity analysis for associations between neighborhood SDOH (Q4: most vulnerable vs. Q1: least vulnerable) and progression of global chronic health condition burden, additionally adjusting for baseline CHC burden

Squares indicate ORs and horizontal lines indicate 95%CIs. Italicized terms denote individual SDOH indicators.

Abbreviations: CI, confidence interval; OR, odds ratio; SDOH, social determinants of health; SVI, Social Vulnerability Index

[a] Adjusted for age at baseline, sex, time since diagnosis, baseline global CHC burden, time between baseline and follow-up, any chemotherapy, any radiotherapy, health insurance coverage, educational attainment, and smoking status.

CHC groups across organ systems by overall and domain of SDOH measures	OR [a] [95% CI]
Onset/worsened CHC group	
**Auditory CHC system**	
Hearing loss group	
SVI Domain: Household composition/disability	1.59 [1.03-2.45]
**Endocrine CHC system**	
Abnormal glucose metabolism group	
SVI Domain: Household composition/disability	1.93 [1.02-3.68]
Obesity/underweight group	
SVI Overall	1.70 [1.18-2.44]
SVI Domain: Socioeconomic status	1.83 [1.28-2.60]
SVI Domain: Housing type/transportation	1.48 [1.06-2.08]
**Hematologic CHC system**	
Hematologic disorder group	
SVI Domain: Minority status/language	3.49 [1.24-9.84]
**Neurologic CHC system**	
Peripheral nervous system disorder group	
SVI Overall	2.07 [1.30-3.28]
SVI Domain: Socioeconomic status	1.88 [1.19-2.97]
SVI Domain: Household composition/disability	1.99 [1.18-3.35]
SVI Domain: Housing type/transportation	1.68 [1.09-2.59]
**Pulmonary CHC system**	
Asthma group	
SVI Domain: Minority status/language	2.70 [1.33-5.49]
Functional pulmonary deficit group	
SVI Domain: Household composition/disability	1.58 [1.03-2.43]
Persistent CHC group	
**Endocrine CHC system**	
Obesity/underweight group	
SVI Overall	1.87 [1.32-2.64]
SVI Domain: Socioeconomic status	2.09 [1.49-2.94]
SVI Domain: Household composition/disability	1.54 [1.09-2.18]
**Neurologic CHC system**	
Central nervous system disorder group	
SVI Domain: Minority status/language	3.06 [1.04-9.01]
**Sexual/reproductive CHC system**	
Disorder of the female reproductive system group	
SVI Overall	6.87 [1.36-34.69]
SVI Domain: Socioeconomic status	9.94 [1.18-83.67]
SVI Domain: Minority status/language	6.37 [1.22-33.44]

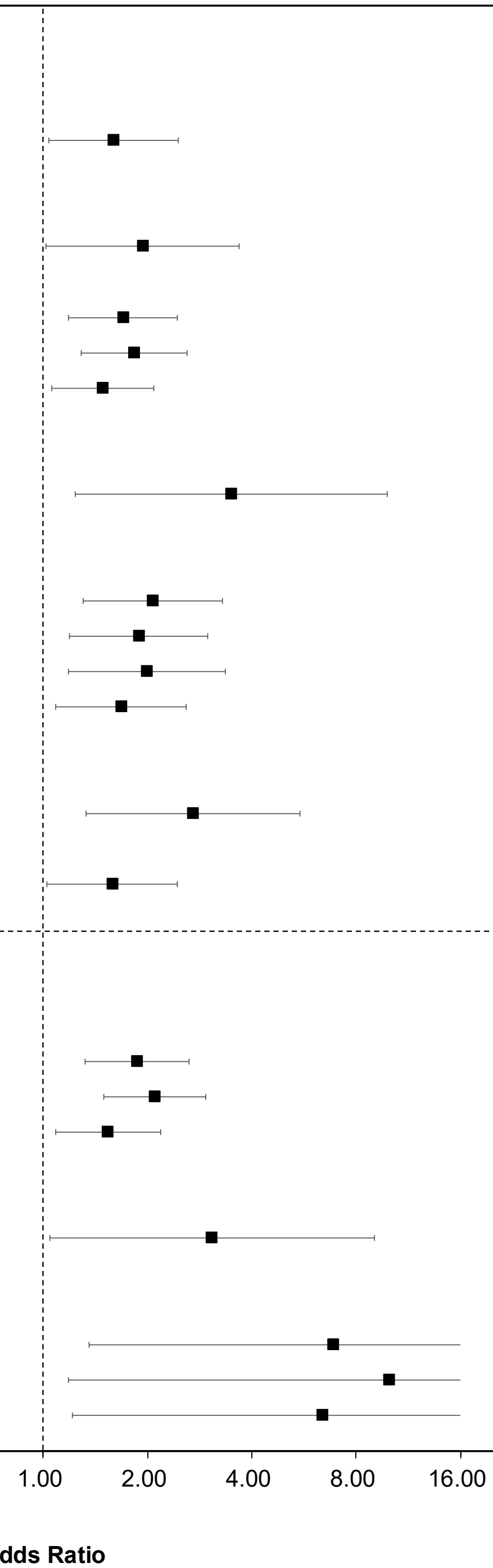

Supplementary Figure 4. Association between overall and domain-specific social vulnerability measures (Q4: most vulnerable vs. Q1: least vulnerable) and changes in chronic health condition groups

This figure presents 19 associations that met the nominal significance threshold (two-sided $P<0.05$) among 440 comparisons. Squares indicate ORs and horizontal lines indicate 95%CIs. Abbreviations: CHC, chronic health condition; CI, confidence interval; OR, odds ratio; SDOH, social determinants of health, SVI, Social Vulnerability Index

[a] Adjusted for age at baseline, sex, time since diagnosis, time between baseline and follow-up, organ-specific treatment for chemotherapy and radiotherapy (see Supplementary Table 4), educational attainment, health insurance coverage, and smoking status.

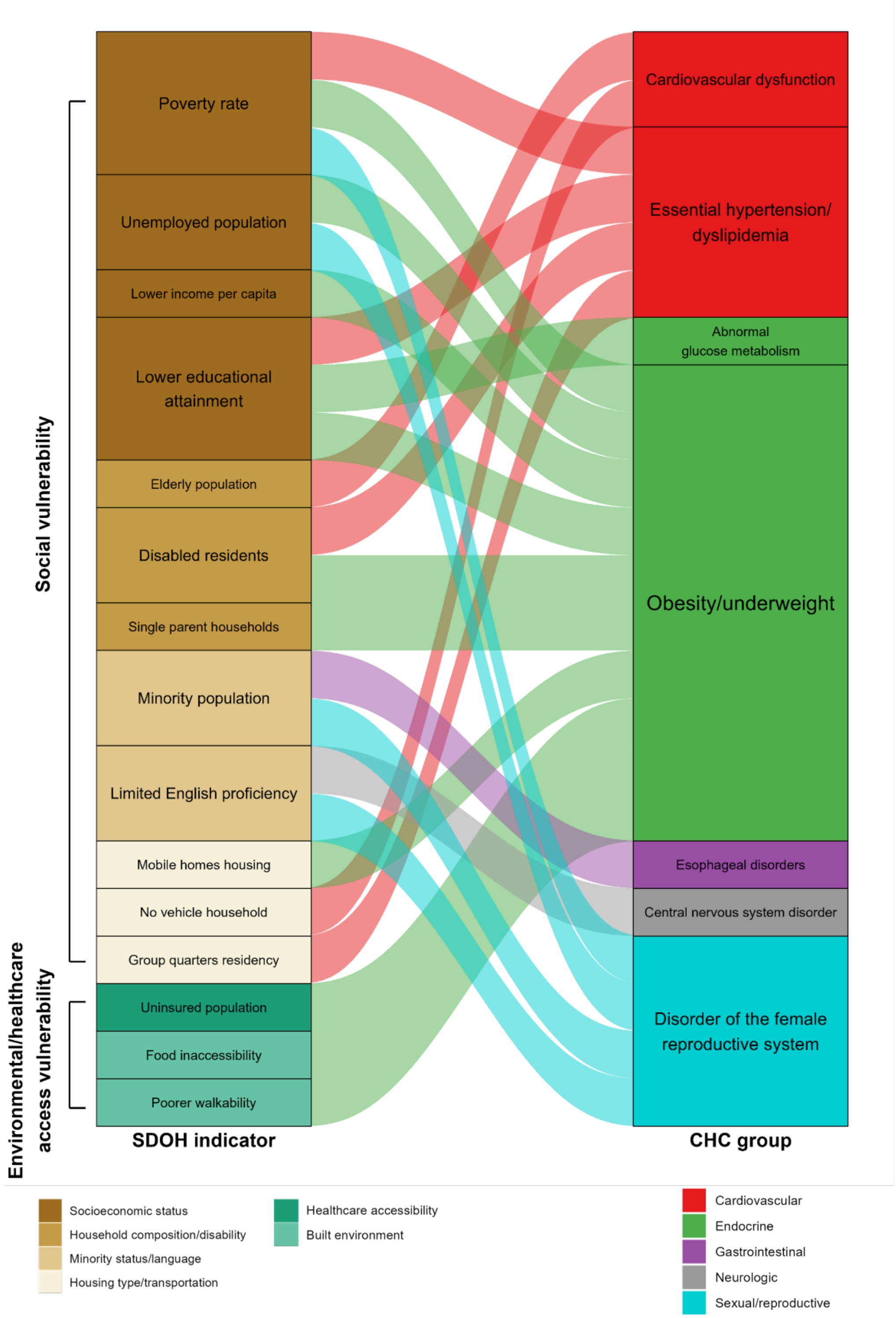

Supplementary Figure 5. Patterns of significant associations between multiple SDOH indicators and persistent CHC groups

Abbreviations: CHC, chronic health condition; SDOH, social determinants of health

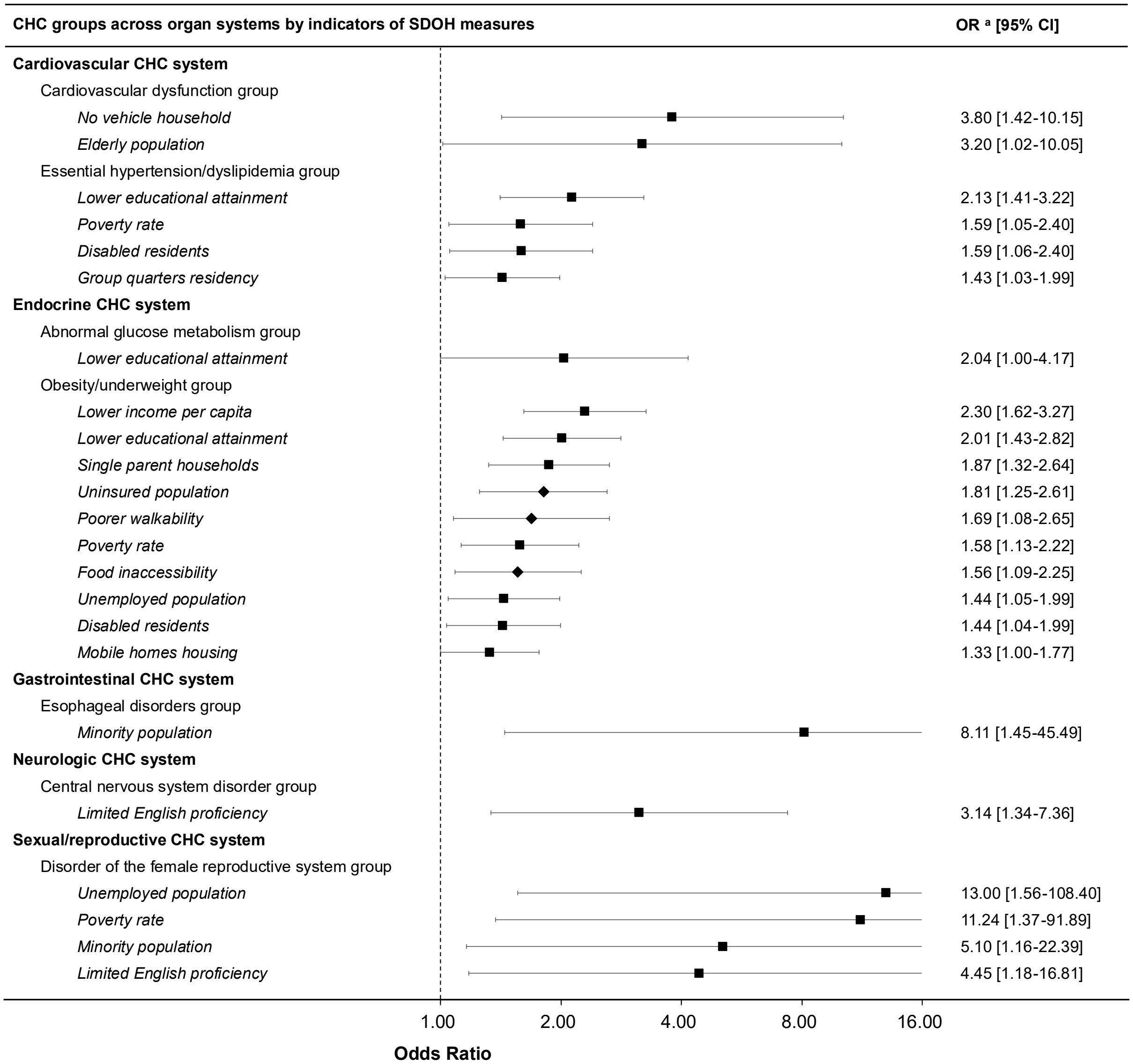

Supplementary Figure 6. Associations between neighborhood SDOH indicators (Q4: most vulnerable vs. Q1: least vulnerable) and persistent chronic health condition groups across organ systems

This figure presents 23 associations that met the nominal significance threshold (two-sided $P<0.05$) among 880 comparisons conducted for persistent CHC groups. Odds ratios are shown as squares for social vulnerability and diamonds for environmental/healthcare access vulnerability, with horizontal lines indicating 95%CIs. Italicized terms denote individual SDOH indicators.

Abbreviations: CHC, chronic health condition; CI, confidence interval; OR, odds ratio; SDOH, social determinants of health

[a] Adjusted for age at baseline, sex, time since diagnosis, time between baseline and follow-up, organ-specific treatment for chemotherapy and radiotherapy (see Supplementary Table 4), health insurance coverage, educational attainment, and smoking status
