# Supplementary material for: Neighborhood disadvantages and longitudinal progression of multimorbidity among adult survivors of childhood cancer: a single-center St. Jude Lifetime Cohort Study in the U.S
Source: eClinicalMedicine. 2026 Oct 13;101:104255. doi: 10.1016/j.eclinm.2026.104255 (PMC13685892; doi:10.1016/j.eclinm.2026.104255)
Supplement: Supplementary file 2 [file mmc2.pdf]

Social Determinants of Health and Progression of Chronic Health Condition in Childhood Cancer Survivors: A Report from the St. Jude Lifetime Cohort Study

Jaesung Choi, PhD
Department of Epidemiology and Cancer Control
Cancer Control and Survivorship Program

Mar 27, 2024

Background

- The overall 5-year survival rate of childhood cancer has reached 85% today.
- Survivors are at risk of a higher burden of chronic health conditions (CHCs) compared to their siblings and the general population.

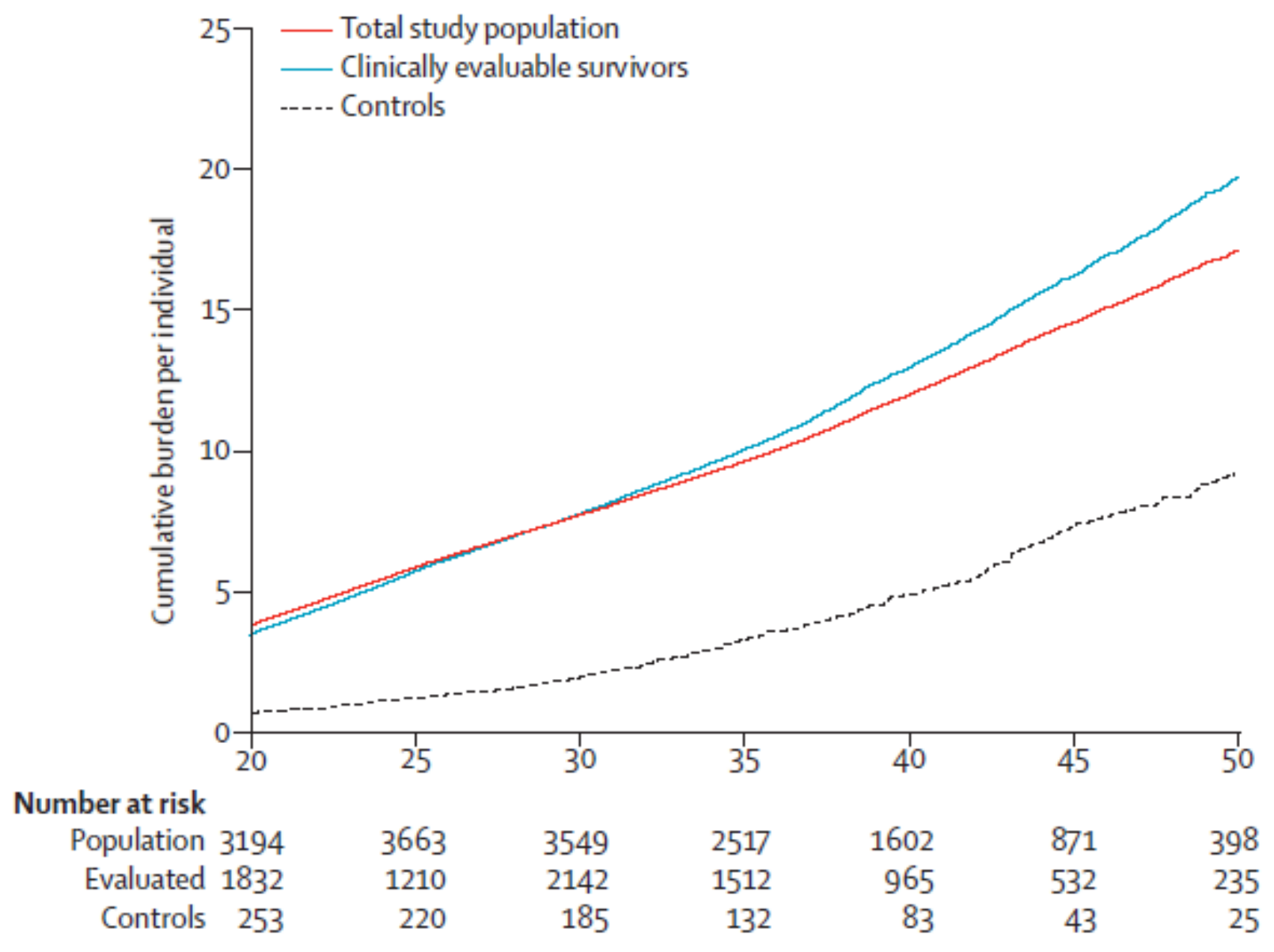

Cumulative burden of grade 1-5 CHC (≥mild)

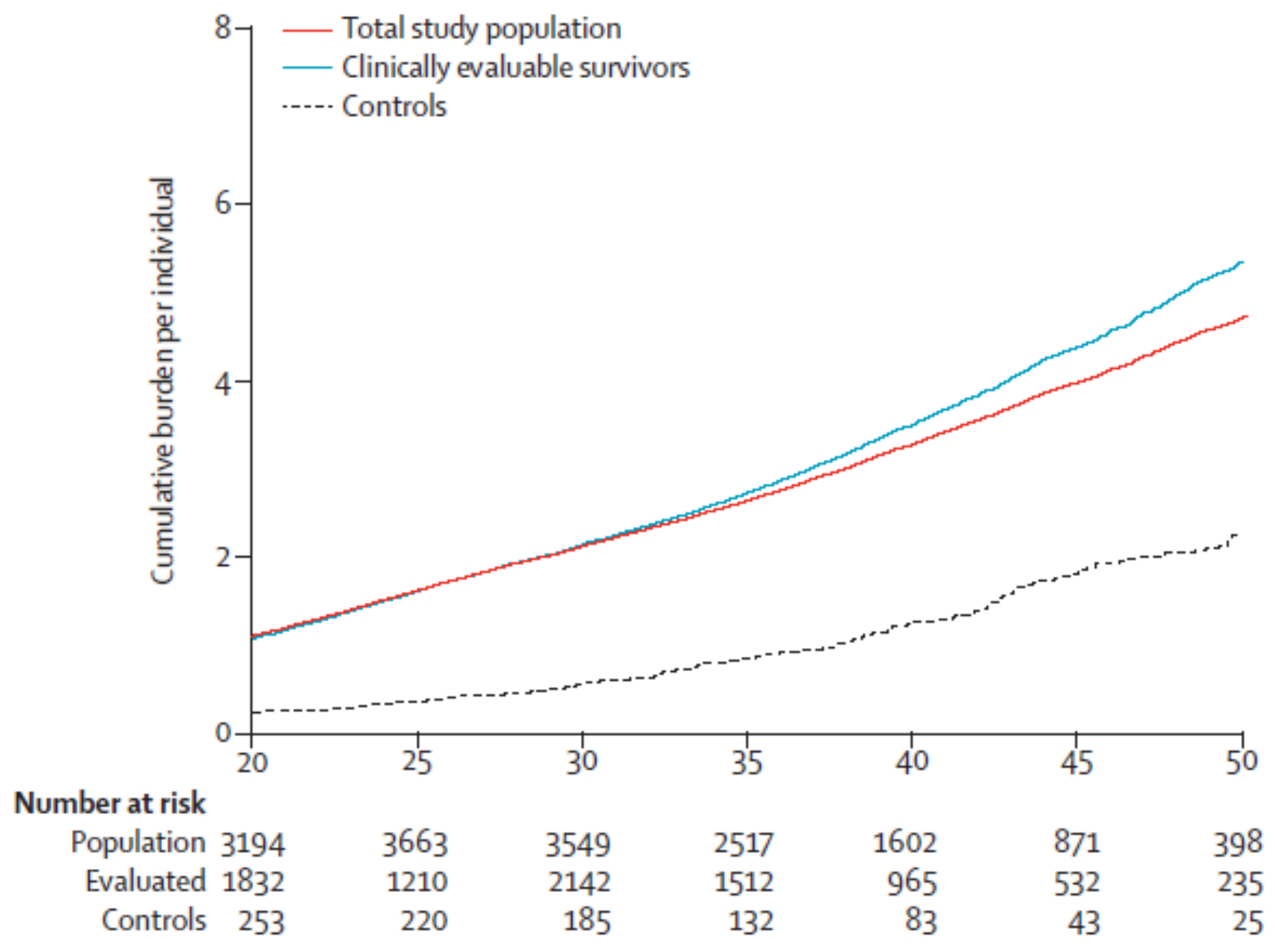

Cumulative burden of grade 3-5 CHC (≥ severe)

Siegel et al. 2023 *CA A Cancer J Clinicians;* Bhakta et al. 2017 *Lancet*

Risk factors for CHCs in CCSS/SJLIFE

St. Jude LIFE St. Jude for Life

- Age, type of cancer diagnosis (Nickhill, et al. 2017 *Lancet*)

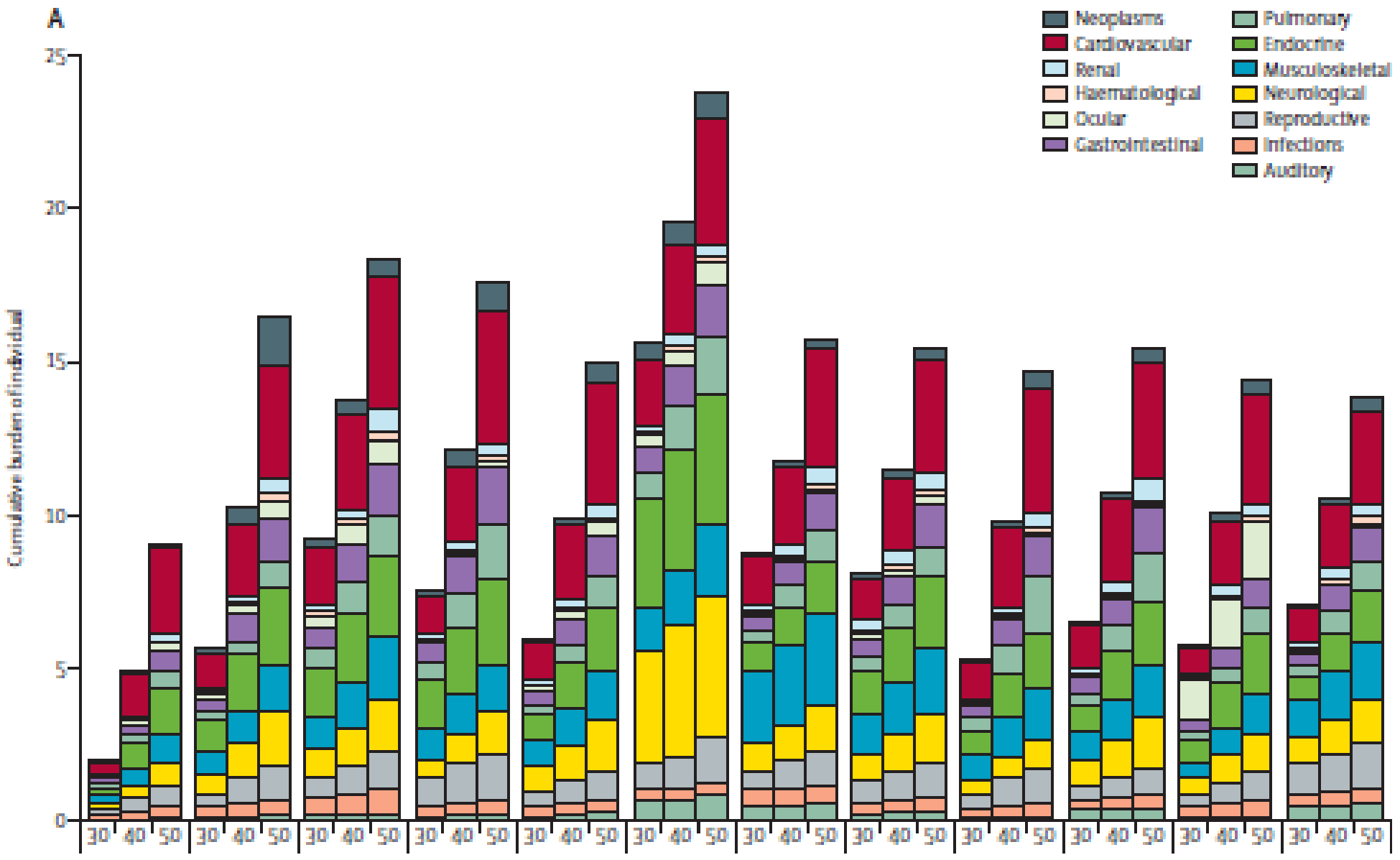

Cumulative burden of Grade 1-5 by age and diagnosis group

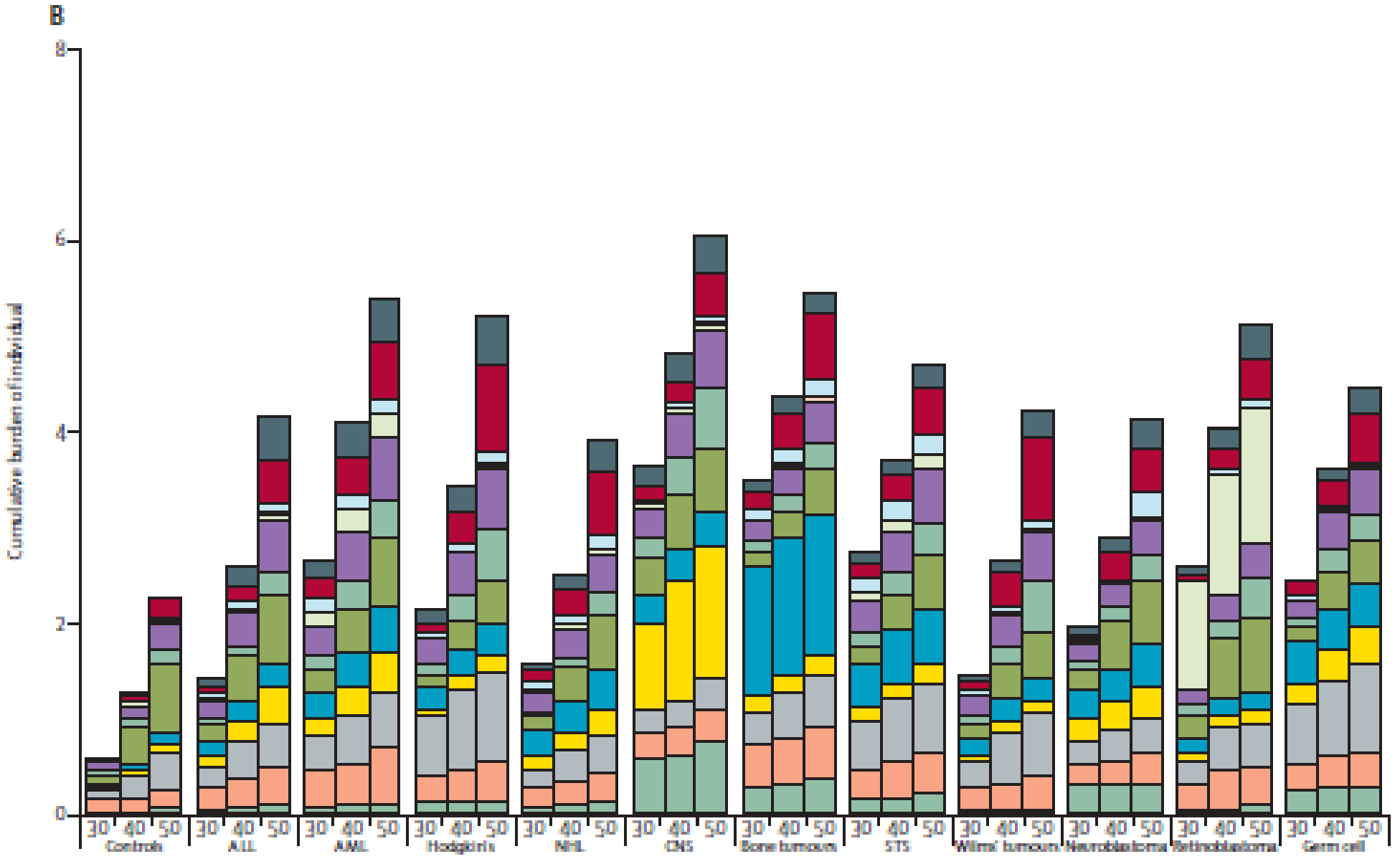

Cumulative burden of Grade 3-5 by age and diagnosis group

Social Determinants of Health at neighborhood-level

St. Jude LIFE
St. Jude for Life

- Neighborhood-level SDOH
  - Neighborhood-level SDOH are emerging as another important source of disparities in which "people are born, live, work, and age born, live, learn, work, play, worship, and age that affect a wide range of health, functioning, and quality-of-life outcomes and risks."

[Healthy People 2030]

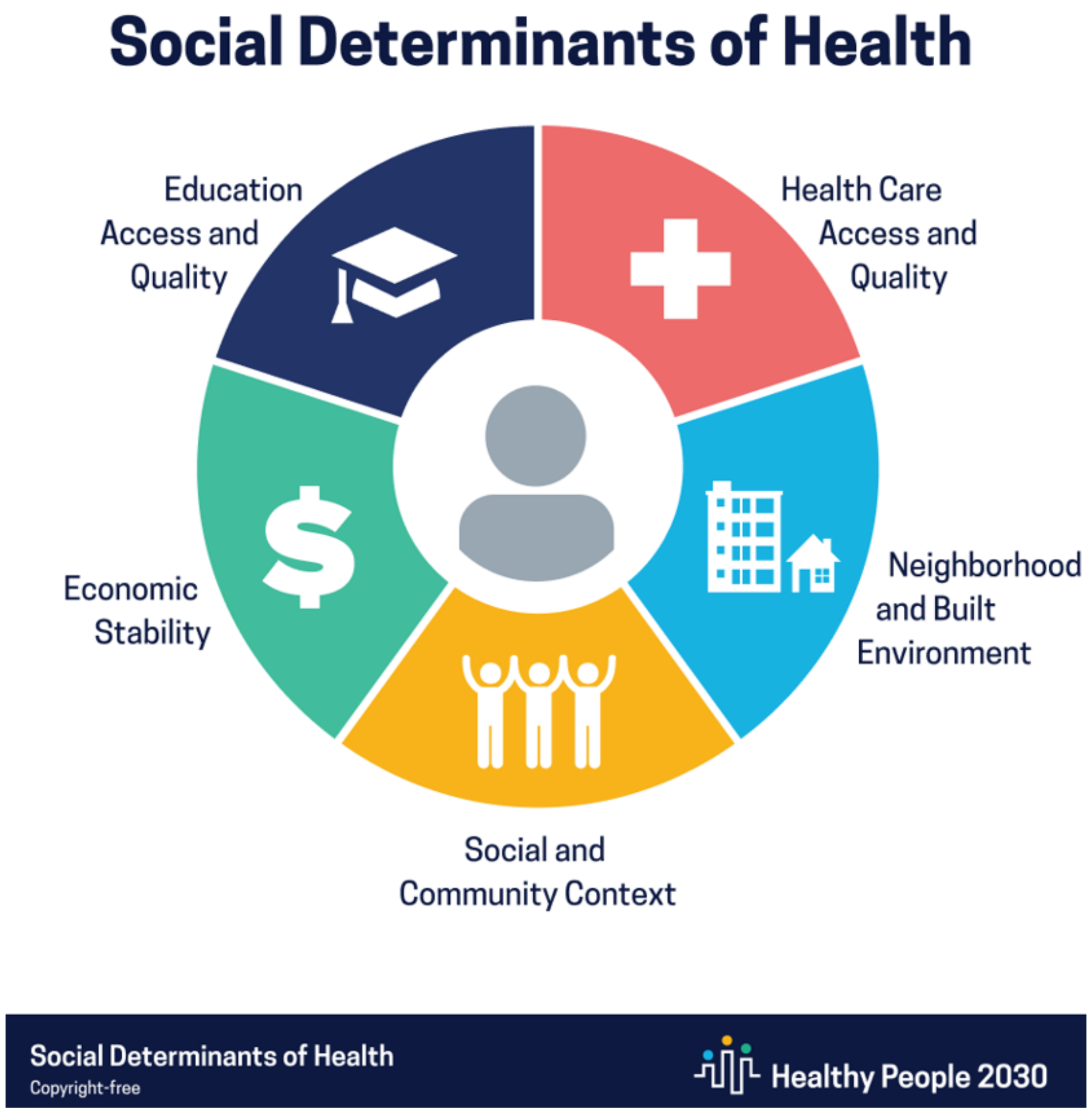

"Social Determinants of Health. Healthy People 2030", U.S. Department of Health and Human Services

Neighborhood-level deprivation in cancer survivors

St. Jude LIFE
St. Jude for Life

- In adult-onset cancer survivors, neighborhood-level SDOH is associated with
  - Unhealthy lifestyle (e.g., alcohol consumption, weight management)
  - Late effects of cancer treatment (physical functioning, chronic medical conditions)
  - Poor psychological well-being (distress, quality of life, medical financial hardship)
  - Surveillance (survival disparities, primary care)

Namin et al. 2021 *Int J Environ Res Public Health*

Neighborhood-level deprivation in cancer survivors

St. Jude LIFE St. Jude for Life

- In childhood cancer survivors, neighborhood-level SDOH is associated with
  - Health-related Quality of Life (Kent et al. 2013 *Qual Life Res;* Horan et al. *JAMA Netw Open* [in press]; Choi et al. *JNCI* under review)
  - Frailty (Schwartz et al. 2023 *CEBP)*
  - Obesity (Howell et al. 2020 *Int J Cancer*)
  - Mortality (Ehrhardt et al. 2023 *JAMA Netw Open)*
- Limitations of the previous studies
  - Not comprehensively analyzing associations between various aspects of SDOH and all types of CHCs
  - Not testing interactive effects between neighborhood-level SDOH and personal SES on CHC progression
  - Collecting cross-sectional data on CHCs, typically using self-reporting methods

Objectives

- To evaluate the associations of neighborhood-level SDOH with the progression of CHCs among childhood cancer survivors who were enrolled in SJLIFE Study
  - Longitudinally assessed clinically-ascertained CHCs by the persistent and worsening/onset status
  - Interaction of neighborhood SDOH with personal SES on the progression of CHCs, by considering cancer diagnosis or treatment exposures

Flow Diagram for Study Sample Inclusion

- Inclusion/exclusion criteria

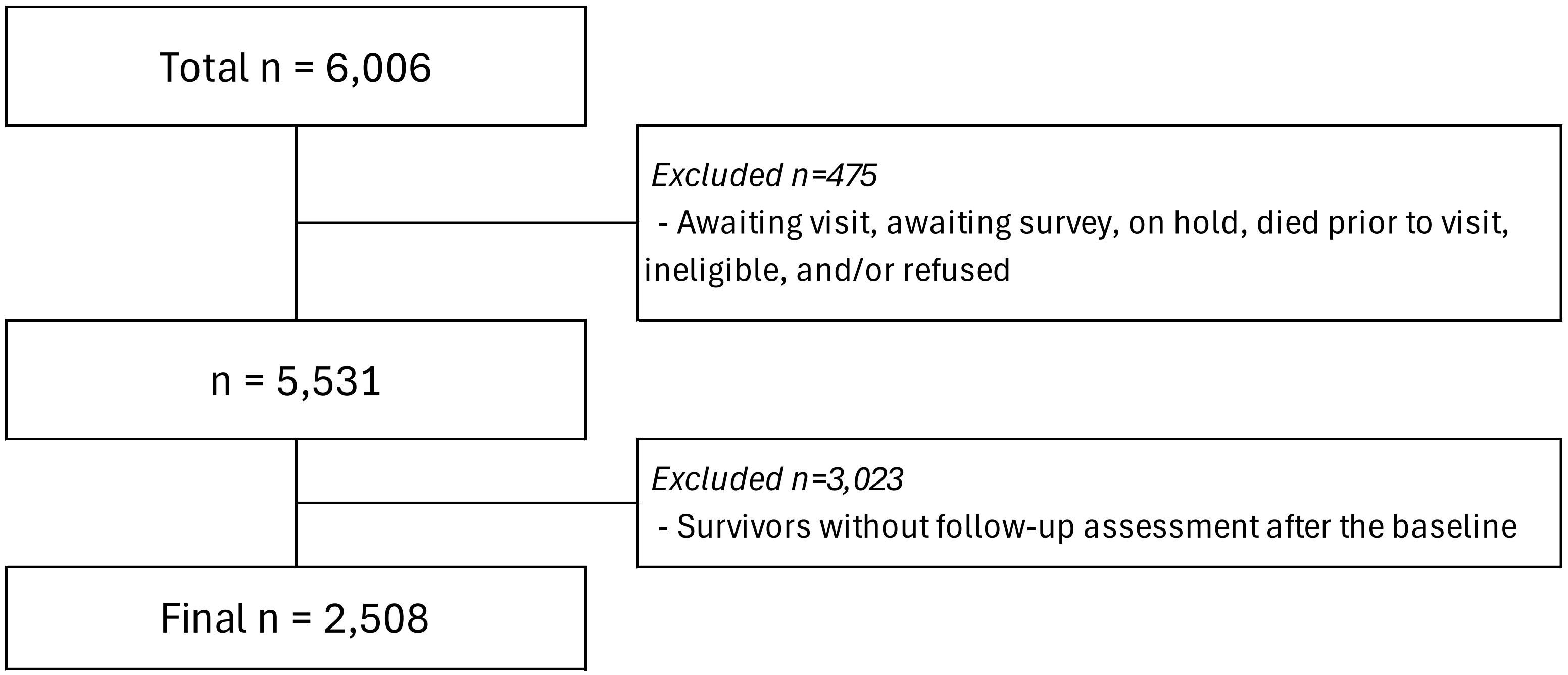

Conceptual Model

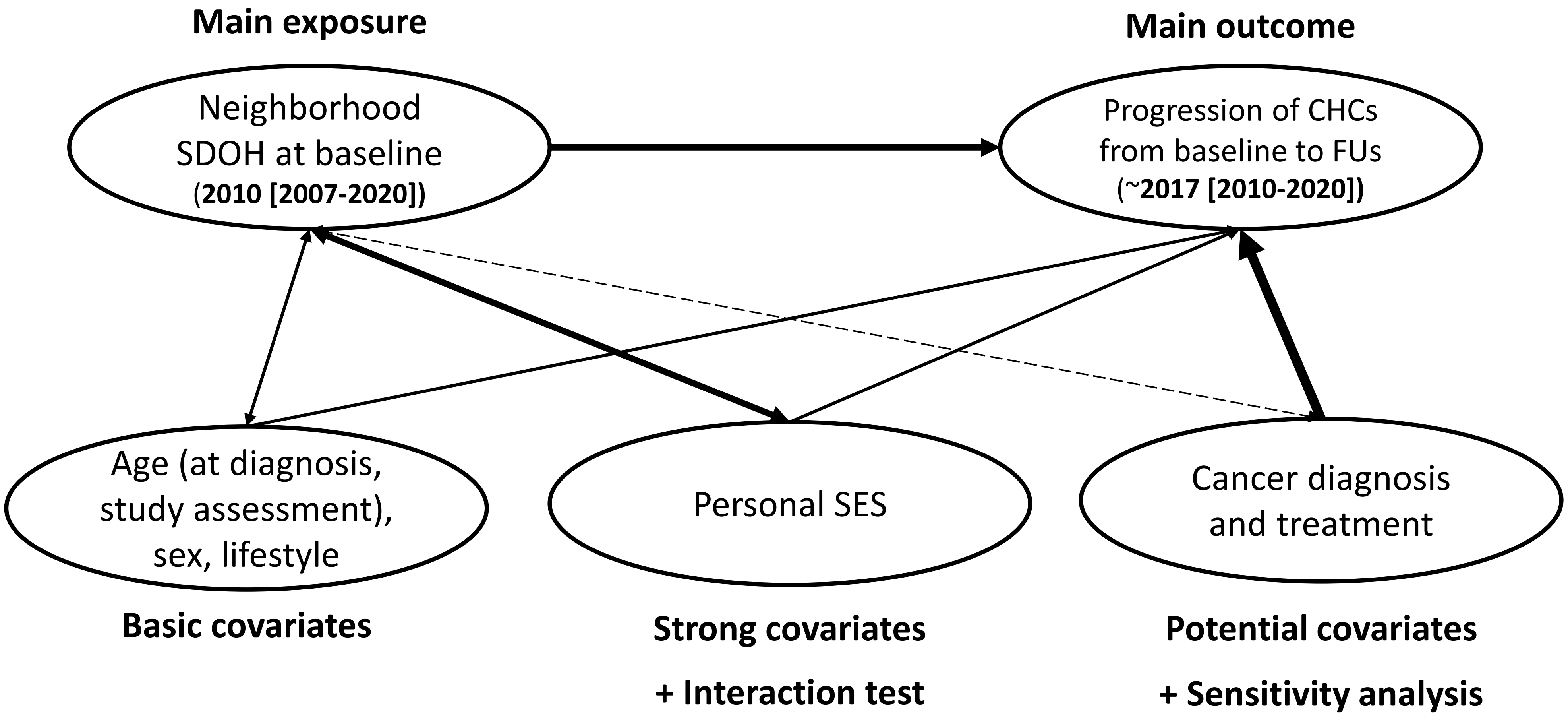

Neighborhood-Level SDOH

St. Jude LIFE
St. Jude for Life

- Content of neighborhood-level SDOH used in this study
  - Social Vulnerability: overall, 4 domains, and 15 indicators of Social Vulnerability Index (SVI)
  - Environmental Vulnerability: 12 indicators by 3 domains

  → These SDOH cover the 5 domains of Health People 2030

- The residential addresses of study samples were geocoded as the levels of U.S. Census Tract (11-digits of FIPS code) and county (5-digits of FIPS code)
- Scores of all SDOH domains and indicators were categorized by quartiles (Q1, Q2, Q3, Q4) according to the national distribution by 25, 50, and 75 percentiles.

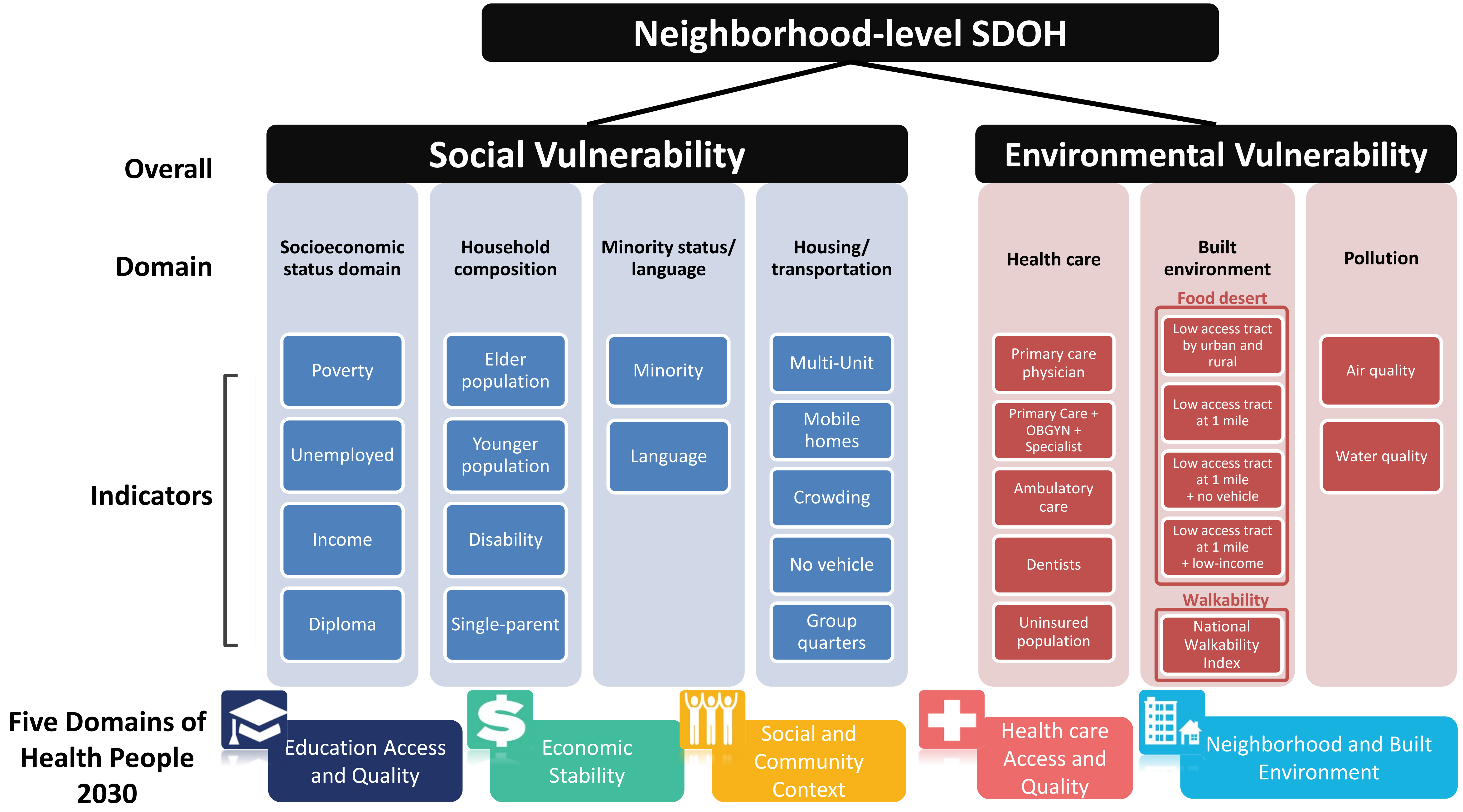
Neighborhood-level SDOH
Overall
Social Vulnerability
Environmental Vulnerability
Domain
Socioeconomic status domain
Household composition
Minority status/ language
Housing/ transportation
Health care
Built environment
Pollution
Indicators
Poverty
Unemployed
Income
Diploma
Elder population
Younger population
Disability
Single-parent
Minority
Language
Multi-Unit
Mobile homes
Crowding
No vehicle
Group quarters
Primary care physician
Primary Care + OBGYN + Specialist
Ambulatory care
Dentists
Uninsured population
Food desert
Low access tract by urban and rural
Low access tract at 1 mile
Low access tract at 1 mile + no vehicle
Low access tract at 1 mile + low-income
Walkability
National Walkability Index
Air quality
Water quality
Five Domains of Health People 2030
Education Access and Quality
Economic Stability
Social and Community Context
Health care Access and Quality
Neighborhood and Built Environment

Content of the Social Vulnerability

Overall

Social Vulnerability Index (SVI)

Domains

Indicators

Socioeconomic status

- Below **Poverty**
- **Unemployed**
- Per capita **Income**
- No high school **Diploma**

Household composition

- **Elder population** (Aged 65 or older)
- **Younger population** (Aged 17 or younger)
- Older than age 5 with a **Disability**
- **Single-parent** households

Minority status/language

- **Minority**
- **Language** (Speak English less than "Very Well")

Housing/ transportation

- **Multi-Unit** structures
- **Mobile homes**
- Crowding (more people than rooms)
- **No vehicle**
- **Group quarters**

- **All indicators were assigned to the census tract**
- **Higher scores in <u>overall</u>, <u>domain</u>, and <u>indicators</u> of SVI represent higher neighborhood-level vulnerability**

Flanagan et al. 2011 *J homeland security and emergency management*

Content of the Environmental Vulnerability

St. Jude LIFE
St. Jude for Life

Domain	Indicator (n=12)	Definition	Data source	Geo unit
Health care	**Primary Care Physician**	Primary Care Physician per 1,000 Residents	2010 HRSA data	Census tract
	**Primary Care + OBGYN + Specialist**	Primary Care + OBGYN + Specialist Physician per 1,000 Residents	2010 HRSA data	Census tract
	**Ambulatory health care**	Ambulatory health care specialists per 1000 people	2010 NANDA	Census tract
	**Dentists**	Dentists per 1000 people	2010 NANDA	Census tract
	**Uninsured population**	Uninsured civilian noninstitutionalized population (%)	2008-2012 ACS	Census tract
Built environment	**Low access tract by urban and rural**	A tract with at least 500 people, or 33 % of the population, living more than one mile (urban areas) or 10 miles (rural areas) from the nearest supermarket, supercenter, or large grocery store. (yes or no)	2010 USDA	Census tract
	**Low access tract at 1 mile**	A tract with at least 500 people, or 33% of the population, living more than one mile from the nearest supermarket, supercenter, or large grocery store. (yes or no)	2010 USDA	Census tract
	**Low access tract at 1 mile + no vehicle**	Housing without vehicle access and low access at 1 mile (%)	2010 USDA	Census tract
	**Low access tract at 1 mile + low-income**	Low-income and low access people at 1 mile (%)	2010 USDA	Census tract
	**National Walkability Index**	Walkability assessed by occupied housing/mix of employment types/street intersection density/predicted commute mode	2013 EPA	Census tract
Pollution	**Air pollution**	Environment Quality Index (EQI)'s Air Quality domain score	2010 EPA	County
	**Water pollution**	Environment Quality Index (EQI)'s Water Quality domain score	2010 EPA	County

HRSA, Health Resources and Services Administration; NANDA, National Neighborhood Data Archive; ACS, American Community Survey; USDA, U.S. Department of Agriculture; EPA, U.S. Environmental Protection Agency

Chronic Health Conditions (CHCs)

St. Jude LIFE
St. Jude for Life

- ~200 individual conditions (severity grade 1-5 by the modified CTCAE)

⬇

- CHC groups by 13 organ systems (severity grade 1-5)
    - Highest grade of individual CHCs in each organ system

⬇

- Global burden of CHCs (classified by none/low/modest/high/very high)
    - Defined by the method in Geenen et al. *JAMA* 2007

Ascertainment of CHCs

- For baseline and each follow-up (FUs), the highest CTCAE grades for individual conditions in an organ system occurred within ± 92 days (3 months) were ascertained.

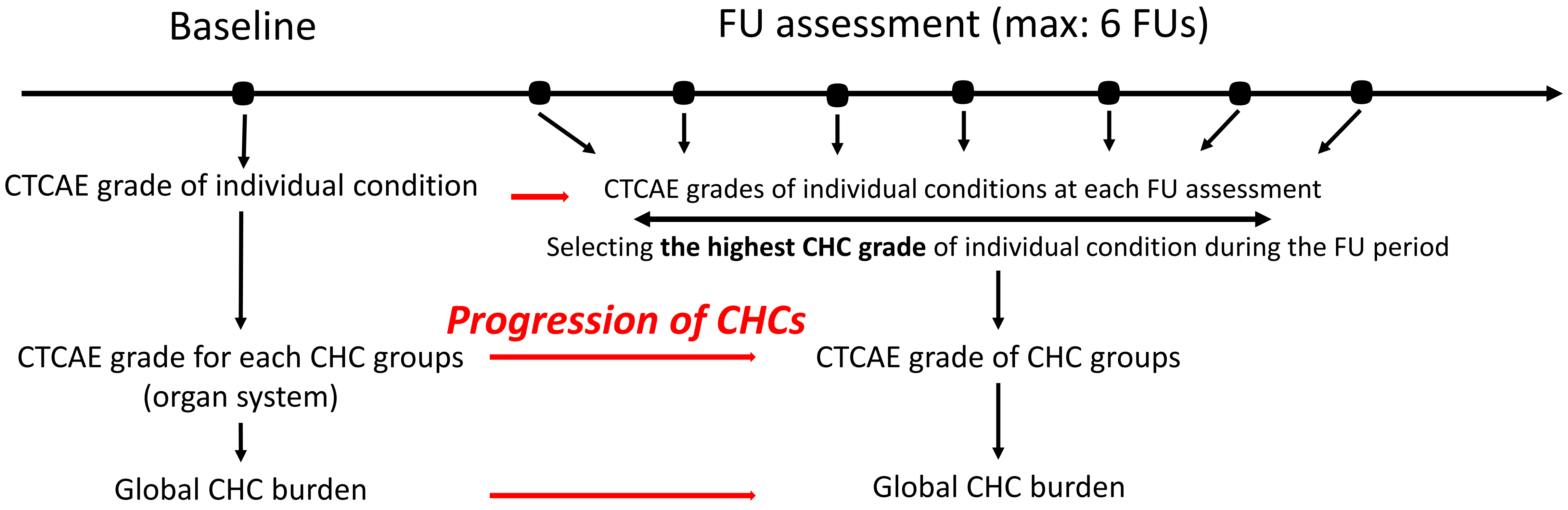

Progression of CHCs Status

St. Jude LIFE
St. Jude for Life

- **Global** CHC burden at baseline → **Global** CHC burden during FUs

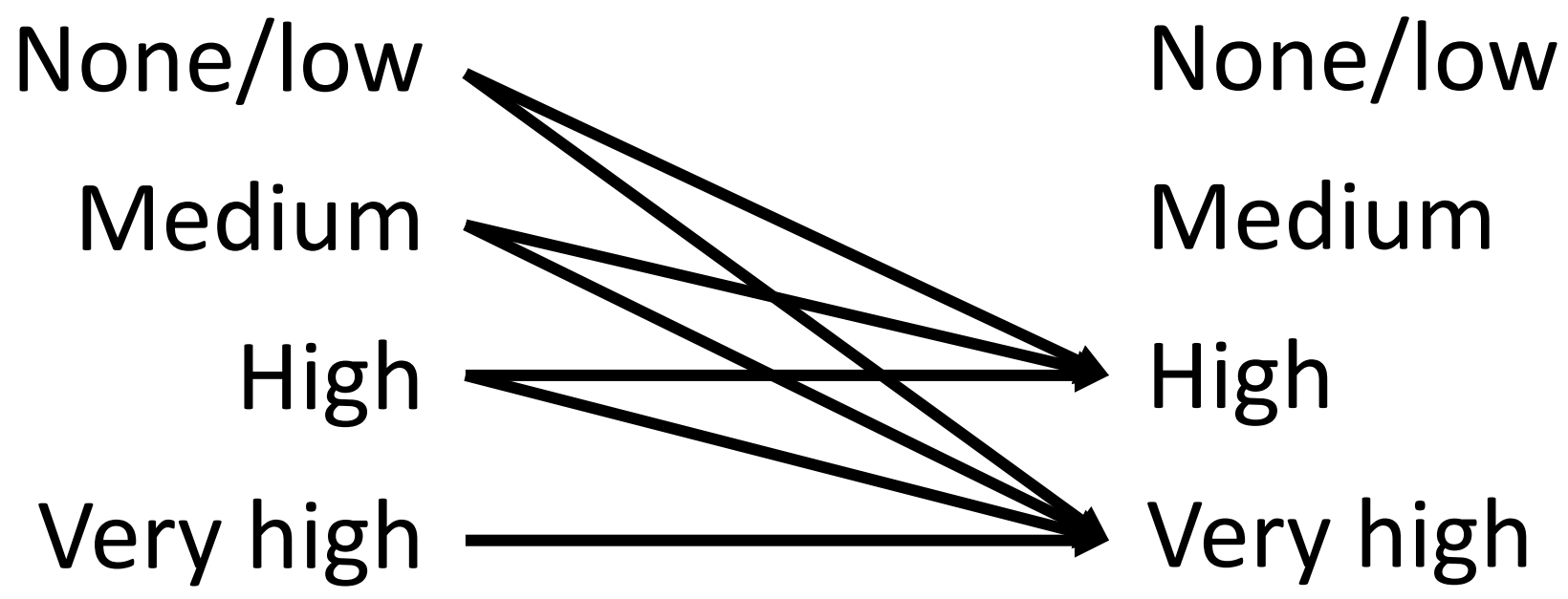

Non-progression (otherwise)

Progression of global CHC burden

- **Group / Individual** CHCs at baseline →
- **Group / Individual** CHCs during FUs

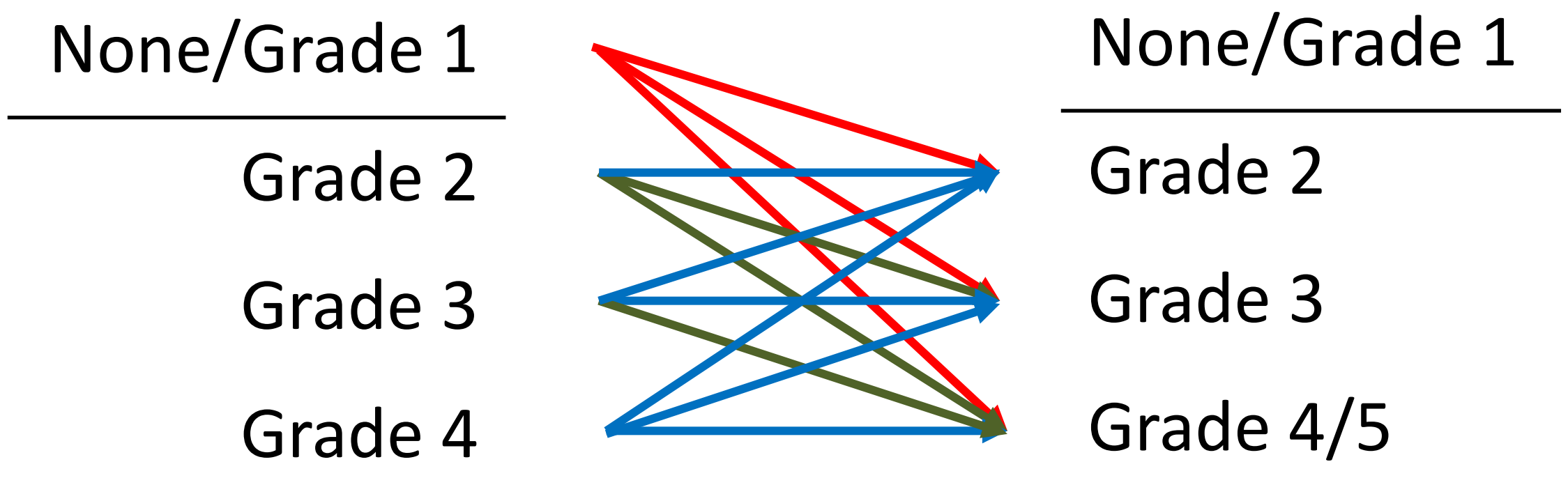

Non-progression (otherwise)

Persistent

Worsening/onset ***(Merged)***

Personal-Level SES

St. Jude
LIFE
St. Jude for Life

- SJLIFE survey
  - Educational attainment (≤ high school, some colleges, and college graduate or above)
  - Health insurance (public insurance, private insurance, and no insurance)

Statistical Analysis

St. Jude LIFE
St. Jude for Life

- Multivariate logistic regression tested the associations between quartiles (Q4 vs. Q1) of neighborhood-level SDOH and the progression in global CHC burden
- Multinomial logistic regression tested the associations between quartiles (Q4 vs. Q1) of neighborhood-level SDOH and persistent and worsening/onset of CHC groups and individual CHCs
  - 13 CHC groups by 13 organ systems
  - All individual CHCs with more than 50 survivors with grade 2^{+} (≥moderate) at FU
- Models
  - Model 1: age at baseline, sex, cancer diagnosis, and time from diagnosis to baseline
  - Model 2: Model 1 + personal SES (educational attainment and health insurance), and smoking status
- Sensitivity analysis (global CHC burden only)
  - **Treatment** model: age at baseline, sex, **treatment (any-chemo and any-radiotherapy)**, time from diagnosis to baseline, personal SES (educational attainment and health insurance), and smoking status

Statistical Analysis

- Interaction between personal SES and neighborhood-level SDOH (Q4 vs. Q1-Q3) on progression of CHCs

Neighborhood-level SDOH
- 1 Social vulnerability overall scores (**Q4 *vs.* Q1-Q3**)
- 4 Social vulnerability domain scores (**Q4 *vs.* Q1-Q3**)
- 27 indicators: social (n=15) and environmental (n=12) vulnerability scores (**Q4 *vs.* Q1-Q3**)

X

Personal SES
- Personal education (**≤high school *vs.* >high school**)
- Insurance (**public insurance *vs.* private/none**)

→ **Outcomes**
  - Global CHC burden
  - 13 CHC groups
  - All individual CHCs with more than 50 survivors with grade 2^+ (≥moderate) at FU
- Adjusted for age at baseline, sex, cancer diagnosis (or treatment), and time from diagnosis to baseline, and smoking status

CONTENTS OF RESULTS

1. Characteristics of study participants
2. SDOH and Progress of Global CHC burden
   - a. Diagnosis model (model 2) and b. treatment model
3. SDOH and group of CHC (organ system)
   - a. Persistent
   - b. Worsening/onset
4. SDOH and individual CHC
   - a. Persistent
   - b. Worsening/onset
5. Interaction of SDOH with personal SES on the CHC
   - a. Persistent
   - b. Worsening/onset

1. Characteristics of Study Participants

St. Jude LIFE
St. Jude for Life

Characteristics	Total (n=2,508) n	(%)
Age at diagnosis, year (mean ± SD)	8.4 ± 5.6	
Age at baseline, year (mean ± SD)	31.6 ± 8.4	
Time from diagnosis to baseline, year (mean ± SD)	23.2 ± 8.2	
Sex		
Male	1,258	(50.2)
Female	1,250	(49.8)
Race and ethnicity		
Non-Hispanic White	2,065	(83.4)
Non-Hispanic Black	346	(14.0)
Hispanic	64	(2.6)
Other	33	(1.3)
Cancer diagnosis		
Leukemia	933	(37.5)
Lymphoma	490	(19.7)
Central nervous system tumor	282	(11.3)
Solid tumors	785	(31.5)
Educational attainment		
≤ High school	697	(29.8)
Some colleges	821	(35.1)
College graduate or above	824	(35.1)
Unknown	166	

	n	(%)
Health insurance		
Public insurance	328	(13.3)
Private insurance	1,612	(65.2)
No insurance	531	(21.5)
Unknown	37	
Current smokers		
Never	1,702	(68.4)
Past	335	(13.5)
Current	453	(18.2)
Unknown	18	
Any-chemotherapy		
No	377	(15.0)
Yes	2,131	(85.0)
Any-radiotherapy		
No	1,032	(41.1)
Yes	1,476	(58.9)

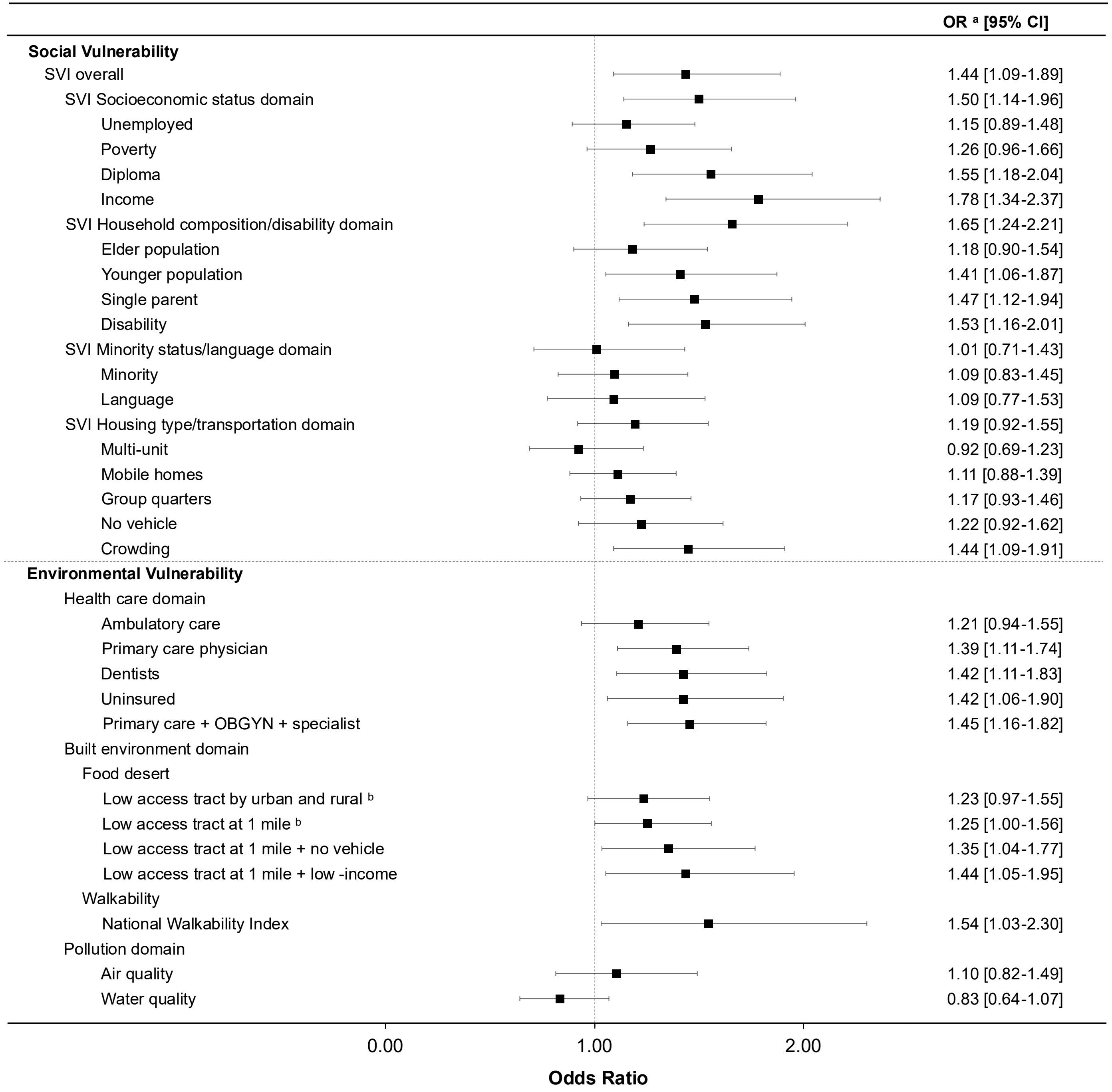

2a. SDOH and Progress of Global CHC Burden (Model 2: w/ diagnosis)

Associations between Q4 of SDOH (vs. Q1 [ref]) and global CHC burden are shown

Abbreviations:

OBGYN, obstetricians and gynecologists

[a] Model 2 adjusted with age at baseline, sex, cancer diagnosis, time from diagnosis to baseline, educational attainment, health insurance, and smoking status

[b] Used as binary variable (yes *vs.* no)

2b. SDOH and Progress of Global CHC Burden (Sensitivity analysis: w/ treatment)

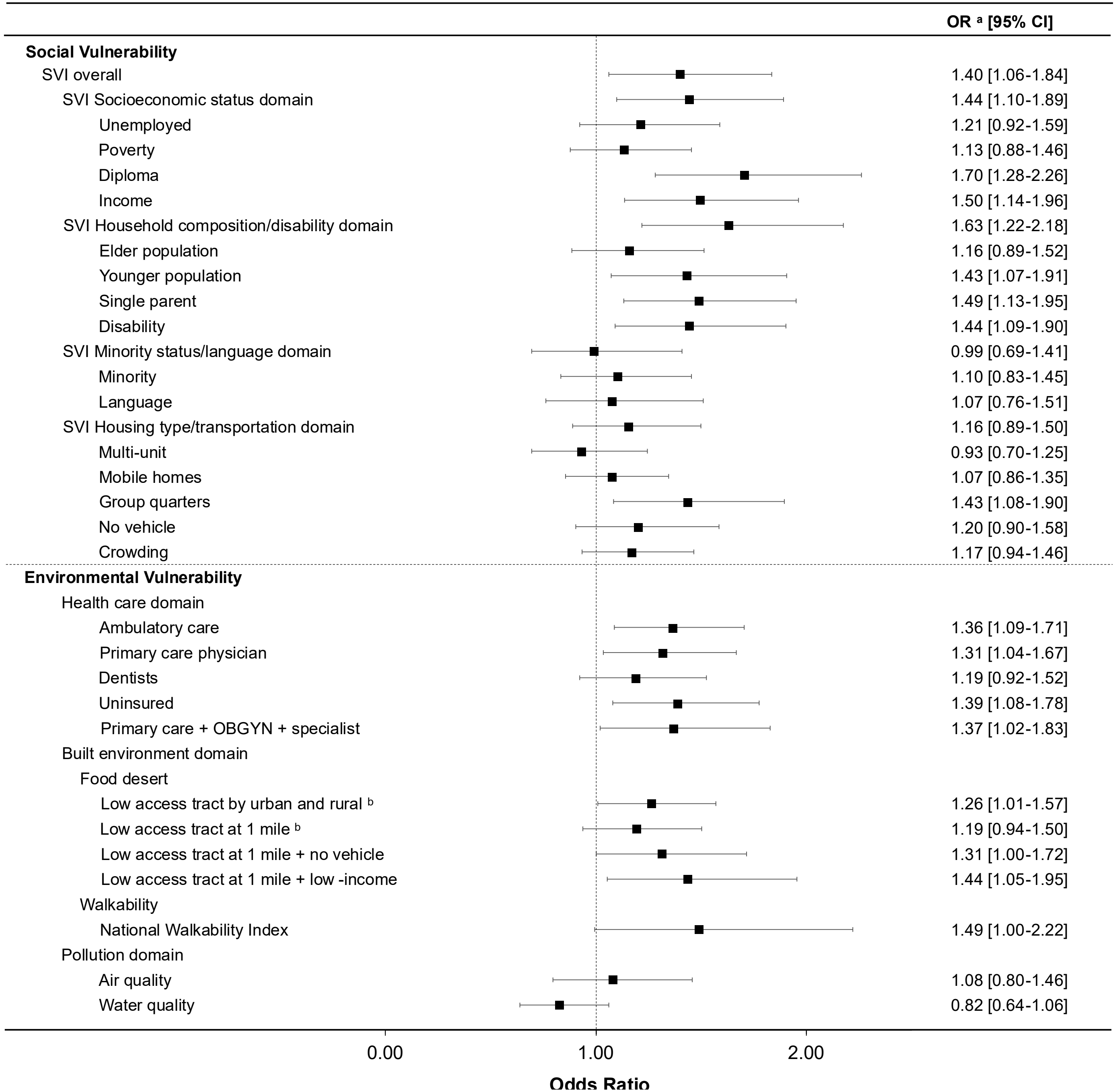

Associations between Q4 of SDOH (vs. Q1 [ref]) and global CHC burden are shown

Abbreviations:
OBGYN, obstetricians and gynecologists

[a] Treatment model adjusted with age at baseline, sex, treatment (any-chemo and any-radiotherapy), time from diagnosis to baseline, educational attainment, and health insurance, and smoking status
[b] Used as binary variable (yes *vs.* no)

3a. SDOH and Persistent Status by CHC Groups

	OR [a] [95% CI]
**Social Vulnerability: Overall and Domains**	
→ Endocrine group	
SVI Overall	1.40 [1.01-1.93]
SVI Household composition/disability domain	1.50 [1.08-2.08]
SVI Socioeconomic status domain	1.62 [1.18-2.22]
**Social and Environmental Vulnerability: Indicators**	
→ Cardiovascular group	
SVI HT: Group quarters	1.38 [1.02-1.88]
→ Endocrine group	
SVI SE: Poverty	1.39 [1.01-1.92]
Env HC: Uninsured	1.45 [1.03-2.05]
Env BE: Walkability	1.54 [1.00-2.39]
SVI SE: Diploma	1.58 [1.15-2.19]
SVI SE: Income	1.64 [1.18-2.30]
SVI HD: Single parent	1.73 [1.25-2.40]
→ Ocular group	
Env HC: Uninsured	1.48 [1.02-2.14]

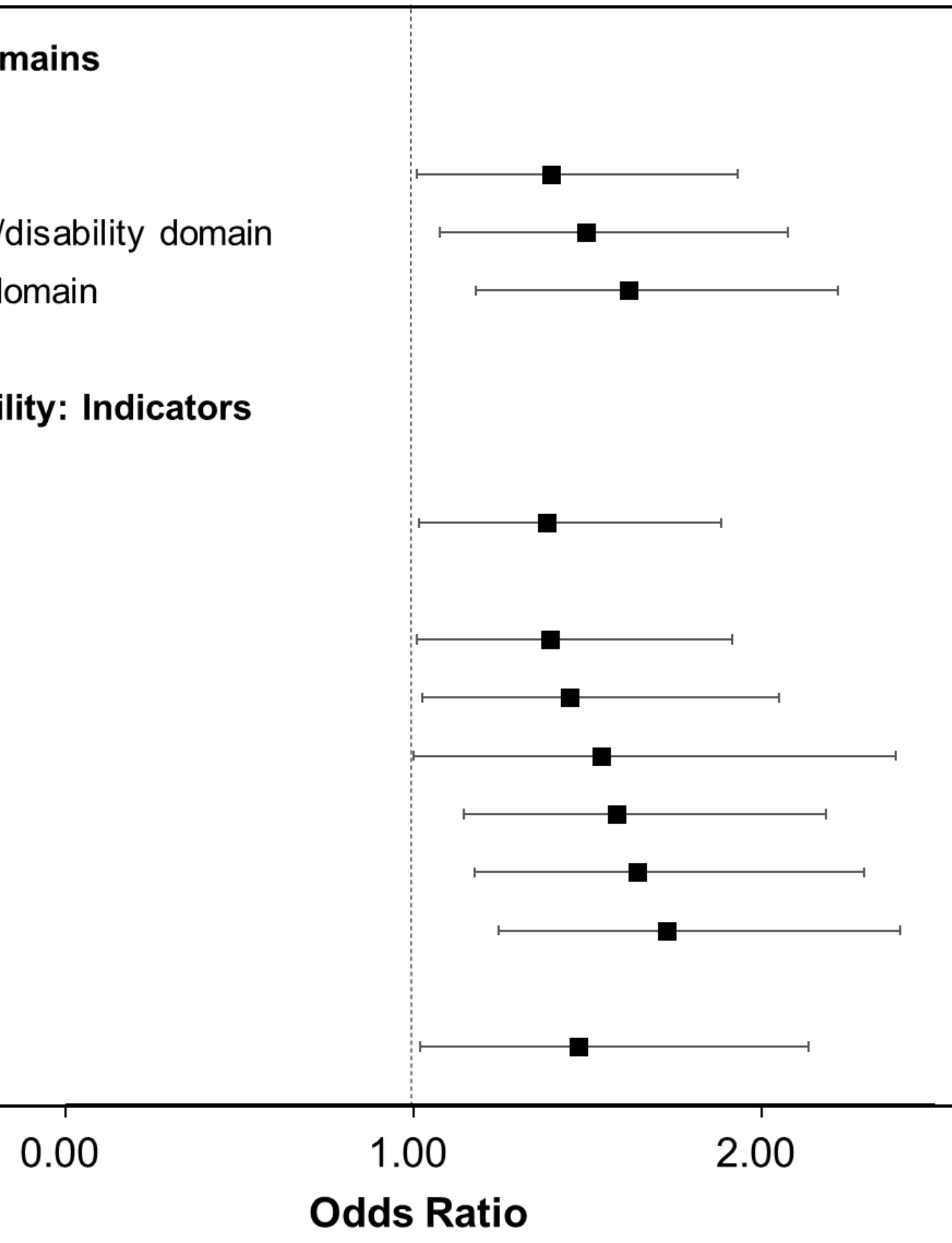

Significant associations between Q4 of SDOH (vs. Q1 [ref]) and persistent of CHC group are shown

Abbreviations:
BE, Built environment;
Env, Environmental Vulnerability;
HC, Health care;
HD, household composition/disability domain;
HT, housing type/ transportation domain;
SE, socioeconomic status domain

[a] Results for Model 2 adjusted with age at baseline, sex, cancer diagnosis, time from diagnosis to baseline, educational attainment, health insurance, and smoking status

3b. SDOH and Worsening/Onset Status by CHC Groups

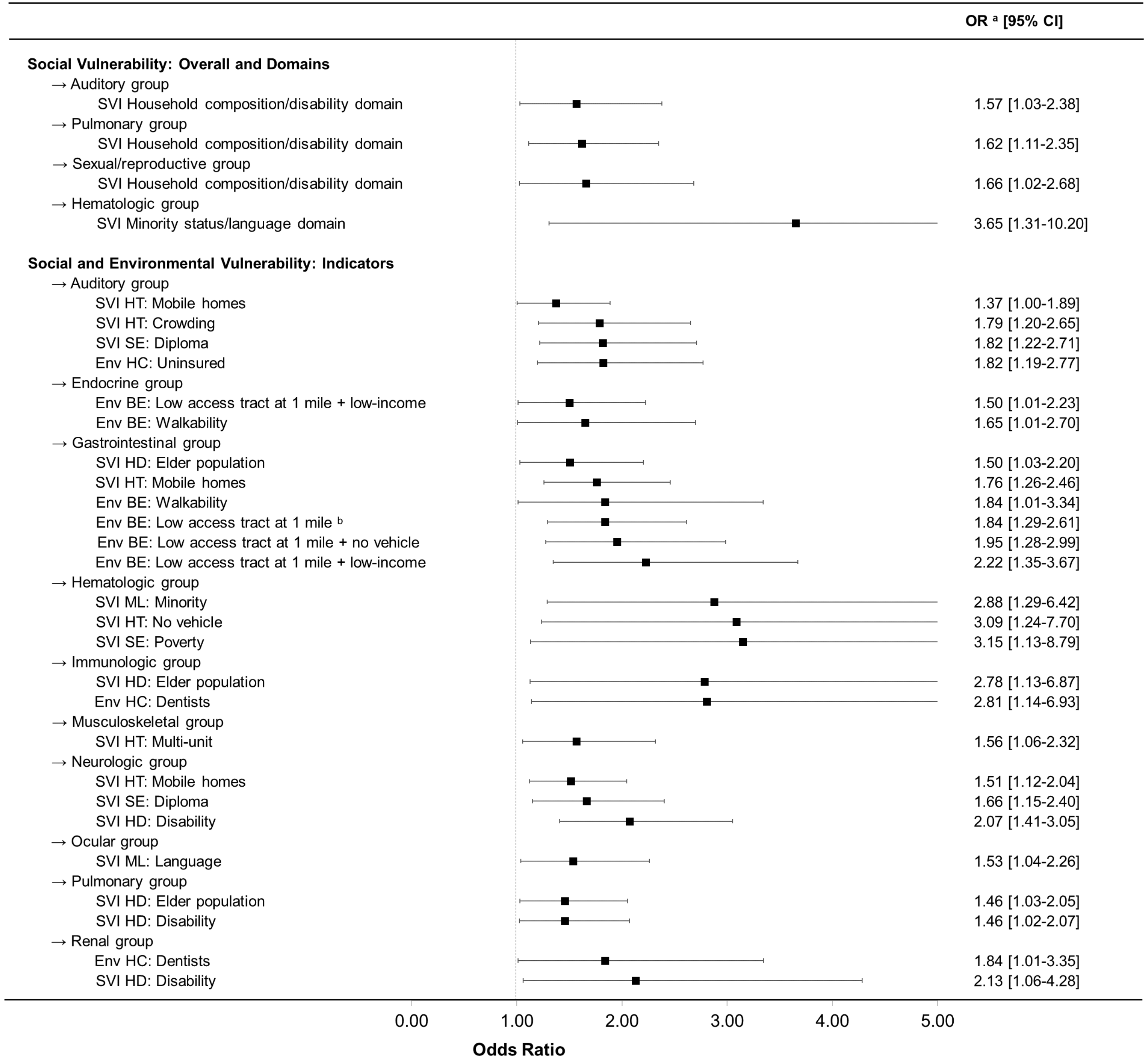

Significant associations between Q4 of SDOH (vs. Q1 [ref]) and worsening/onset of individual CHC are shown

Abbreviations:

BE, Built environment
Env, Environmental Vulnerability;
HC, health care domain;
HD, household composition/disability domain;
HT, housing type/transportation domain;
ML, minority status/language domain;
SE, socioeconomic status

[a] Results for Model 2 adjusted with age at baseline, sex, cancer diagnosis, time from diagnosis to baseline, educational attainment, health insurance, and smoking status

4a. SDOH and Persistent Status by Individual CHCs

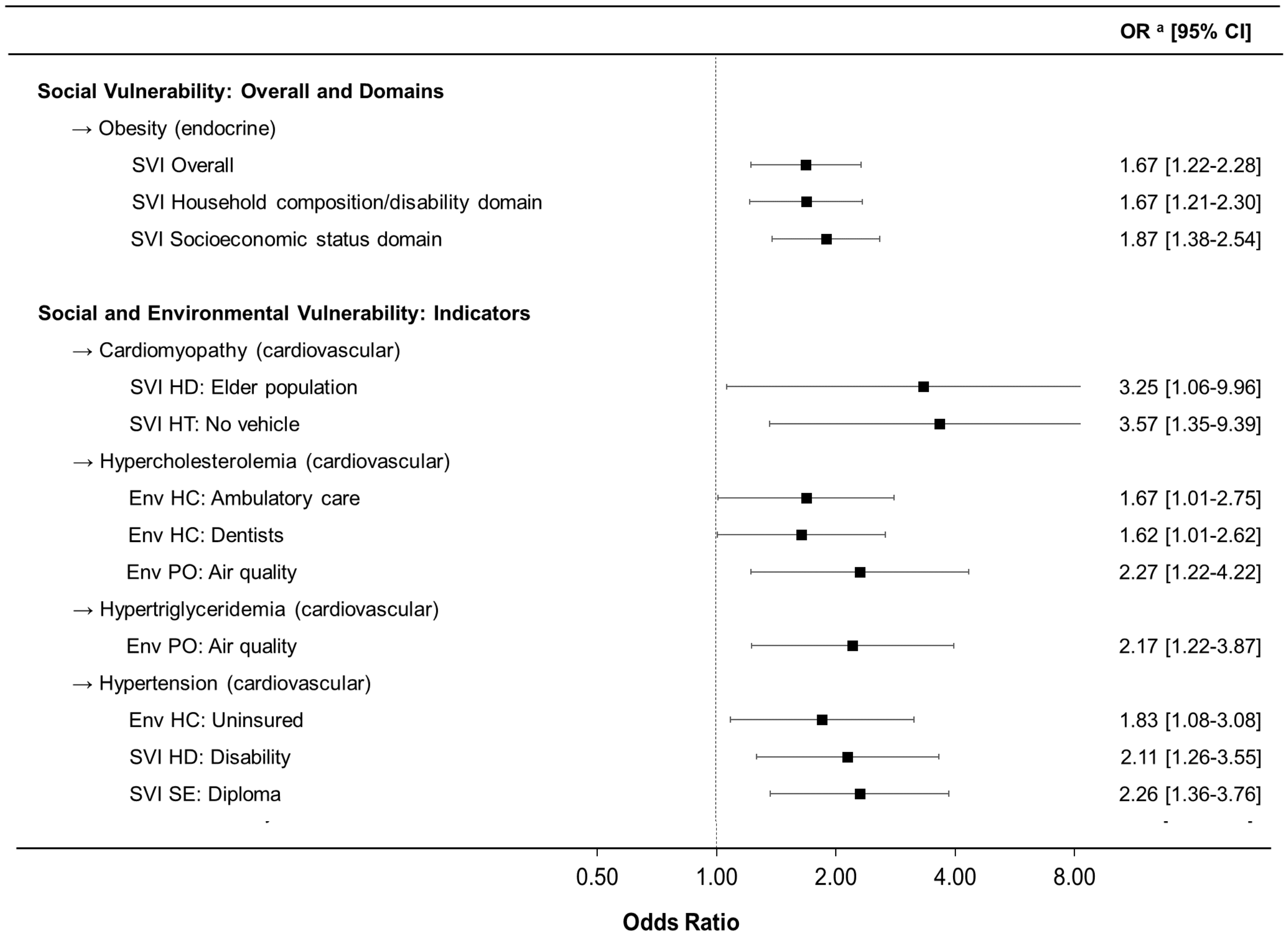

Significant associations between Q4 of SDOH (vs. Q1 [ref]) and persistent of individual CHC are shown

Abbreviations:

Env, Environmental Vulnerability;
HC, health care domain;
HD, household composition/disability domain;
HT, housing type/transportation domain;
PO, pollution domain
SE, socioeconomic status

[a] Results for Model 2 adjusted with age at baseline, sex, cancer diagnosis, time from diagnosis to baseline, educational attainment, health insurance, and smoking status

<continued.>

4a. SDOH and Persistent Status by Individual CHCs

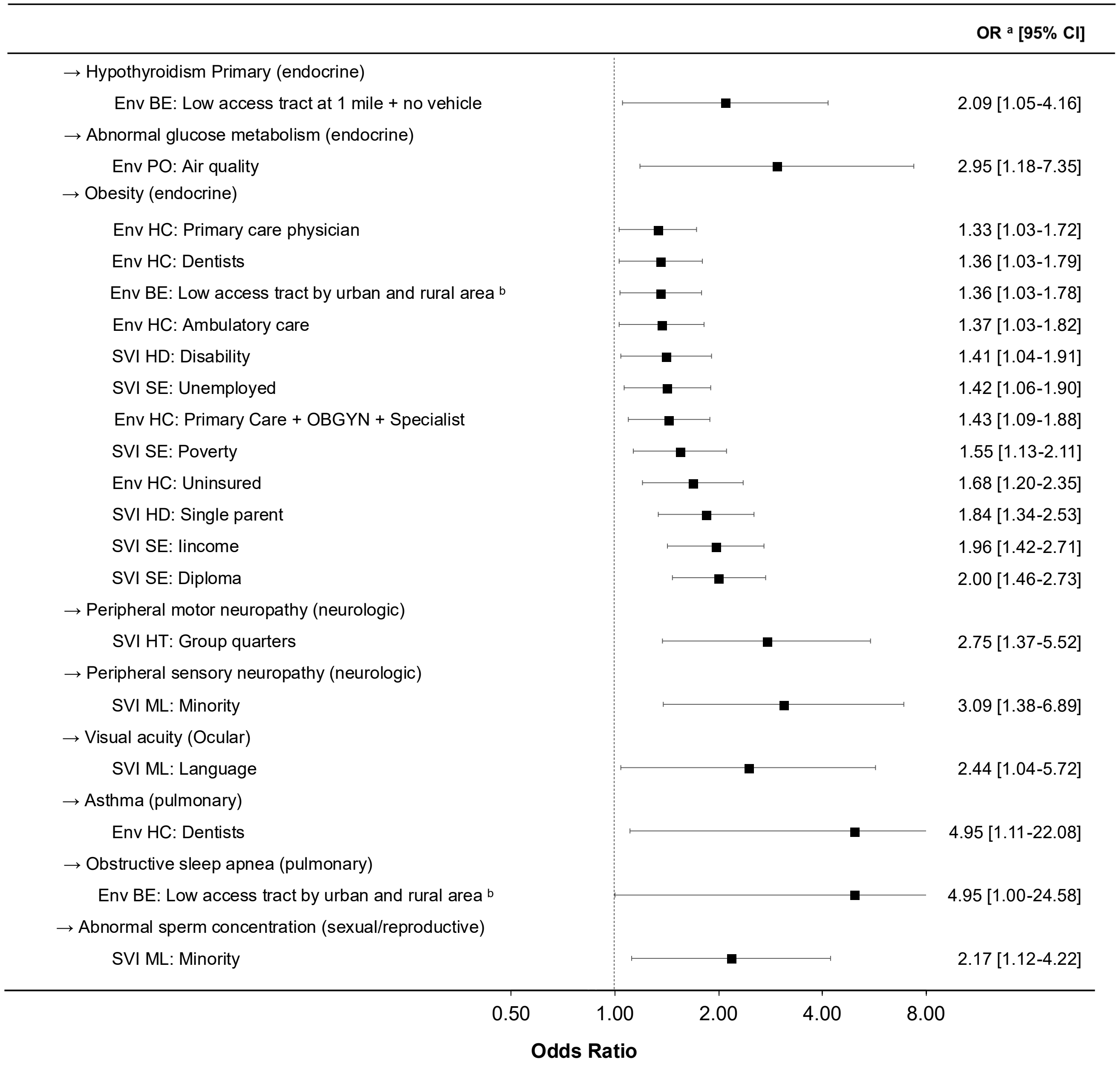

Significant associations between Q4 of SDOH (vs. Q1 [ref]) and persistent of individual CHC are shown

Abbreviations:

BE, built environment domain;
Env, Environmental Vulnerability;
HC, health care domain;
HD, household composition/disability domain;
HT, housing type/transportation domain

[a] Results for Model 2 adjusted with age at baseline, sex, cancer diagnosis, time from diagnosis to baseline, educational attainment, health insurance, and smoking status

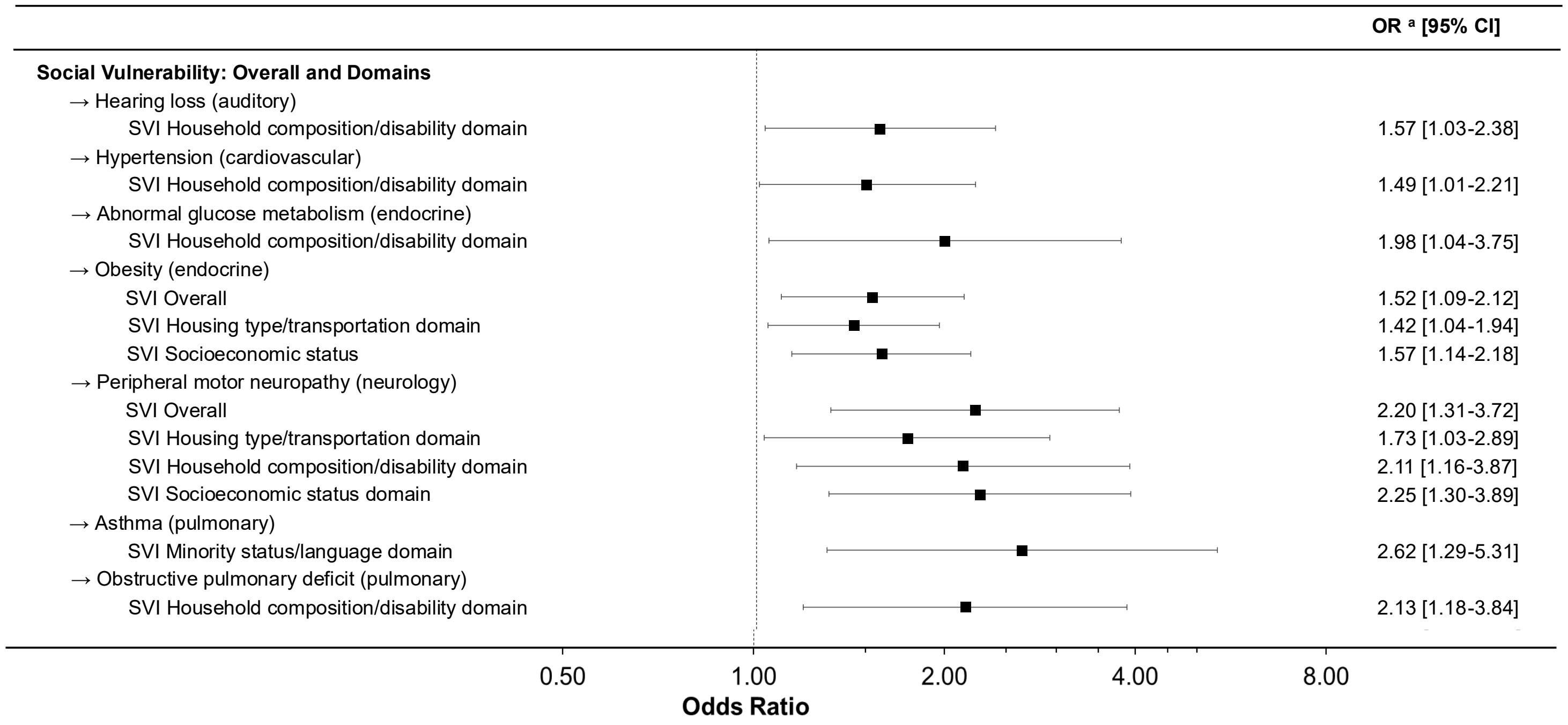

4b. SDOH and Worsening/Onset Status by Individual CHCs

Significant associations between Q4 of SDOH (vs. Q1 [ref]) and worsening/onset of individual CHC are shown

[a] Results for Model 2 adjusted with age at baseline, sex, cancer diagnosis, time from diagnosis to baseline, educational attainment, health insurance, and smoking status

<continued.>

4b. SDOH and Worsening/Onset Status by Individual CHCs

Individual CHC (of endocrine and gastrointestinal)

Social and Environmental Vulnerability: Indicators	OR [a] [95% CI]
→ Hearing Loss (auditory)	
SVI HT: Mobile homes	1.37 [1.00-1.89]
SVI HT: Crowding	1.79 [1.20-2.65]
SVI SE: Diploma	1.82 [1.22-2.71]
Env HC: Uninsured	1.82 [1.19-2.77]
→ Cardiomyopathy (cardiovascular)	
Env HC: Primary care physician	1.52 [1.00-2.31]
→ Hypertension (cardiovascular)	
SVI HD: Disability	1.48 [1.02-2.14]
SVI ML: Language	1.60 [1.02-2.49]
SVI SE: Diploma	1.62 [1.11-2.37]
Env HC: Uninsured	1.65 [1.09-2.51]
→ Hypothyroidism primary (endocrine)	
Env PO: Water quality	1.64 [1.09-2.47]
→ Abnormal glucose metabolism (endocrine)	
SVI HD: Single parent	1.86 [1.05-3.30]
SVI SE: Diploma	1.90 [1.11-3.27]
Env HC: Ambulatory care	1.91 [1.14-3.19]
Env BE: Low access tract at 1 mile + low-income	2.11 [1.03-4.32]
Env BE: Walkability	4.61 [1.42-15.01]
→ Obesity (endocrine)	
SVI HT: Mobile homes	1.34 [1.02-1.78]
SVI HD: Single parent	1.39 [1.00-1.94]
SVI HT: Crowding	1.41 [1.00-1.98]
SVI SE: Diploma	1.42 [1.02-1.97]
Env BE: Low access tract at 1 mile + low-income	1.71 [1.17-2.50]
Env BE: Walkability	1.72 [1.05-2.84]
→ Gastroesophageal reflux disease (gastrointestinal)	
Env BE: Low access tract at 1 mile + no vehicle	1.67 [1.07-2.60]
SVI HT: Mobile homes	1.67 [1.17-2.39]
Env BE: Low access tract at 1 mile [b]	1.97 [1.34-2.91]
Env BE: Low access tract at 1 mile + low-income	2.11 [1.23-3.63]
Env BE: Walkability	2.32 [1.13-4.77]

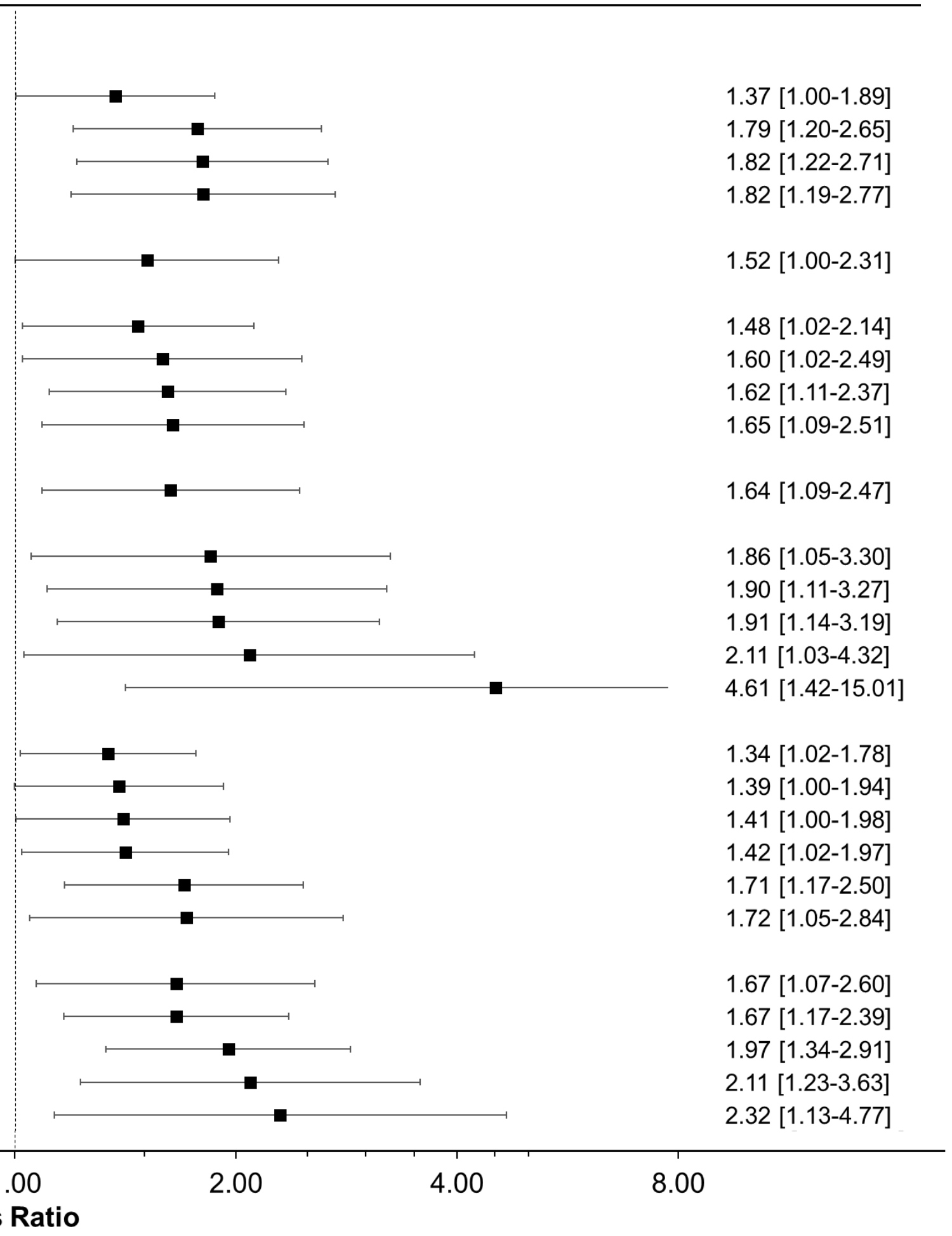

Significant associations between Q4 of SDOH (vs. Q1 [ref]) and worsening/onset of individual CHC are shown

Abbreviations:

BE, built environment domain;
Env, Environmental Vulnerability;
HC, health care domain;
HD, household composition/disability domain;
HT, housing type/transportation domain;
SE, socioeconomic status domain

[a] Results for Model 2 adjusted with age at baseline, sex, cancer diagnosis, time from diagnosis to baseline, educational attainment, health insurance, and smoking status

<continued.>

4b. SDOH and Worsening/Onset Status by Individual CHCs

Individual CHC (neurology, ocular, pulmonary, and renal)

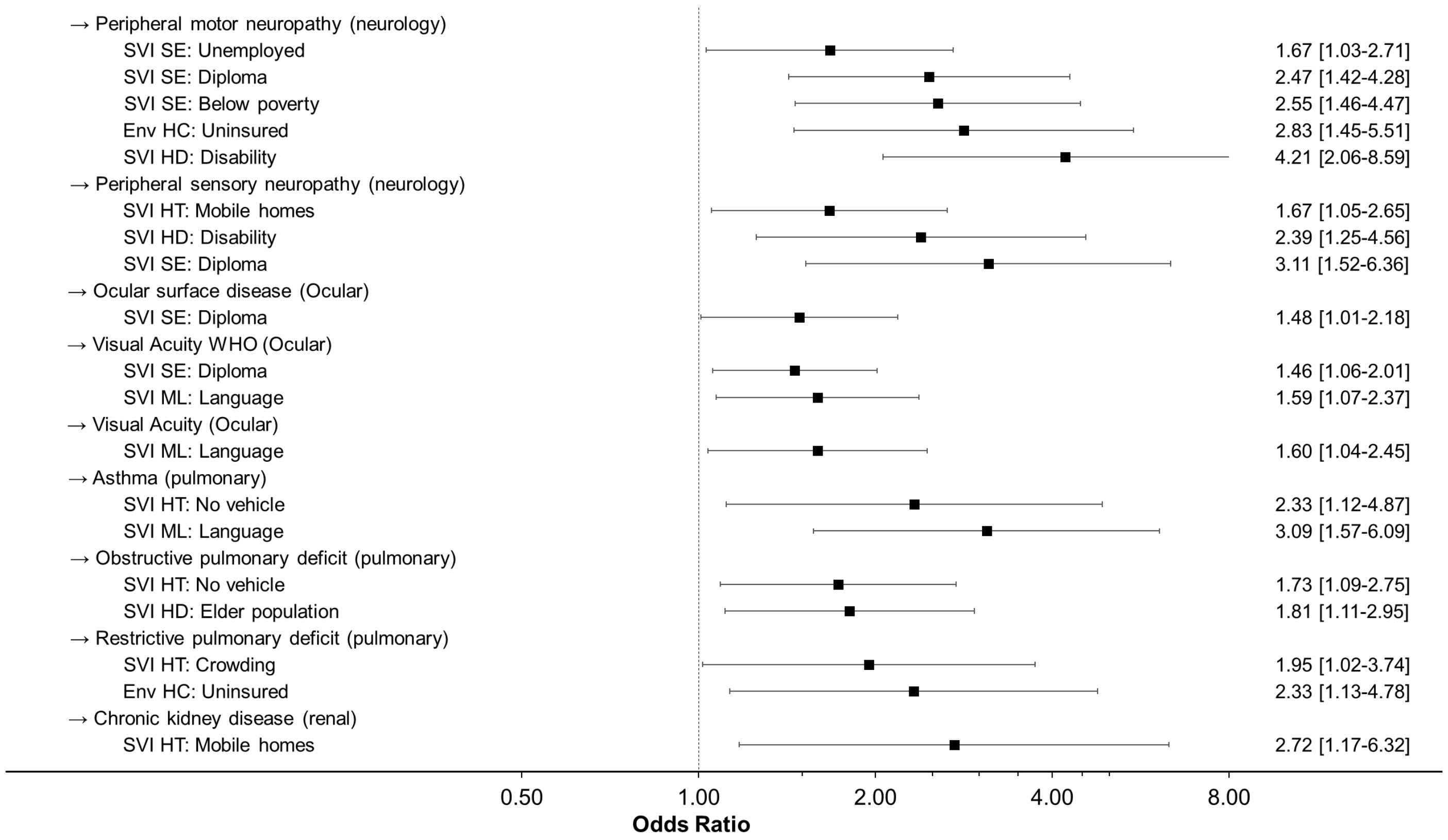

Significant associations between Q4 of SDOH (vs. Q1 [ref]) and worsening/onset of individual CHC are shown

Abbreviations:
Env, Environmental Vulnerability;
HC, health care domain;
HD, household composition/disability domain;
HT, housing type/transportation domain;
ML, minority status/language domain;
SE, socioeconomic status domain

[a] Results for Model 2 adjusted with age at baseline, sex, cancer diagnosis, time from diagnosis to baseline, educational attainment, health insurance, and smoking status

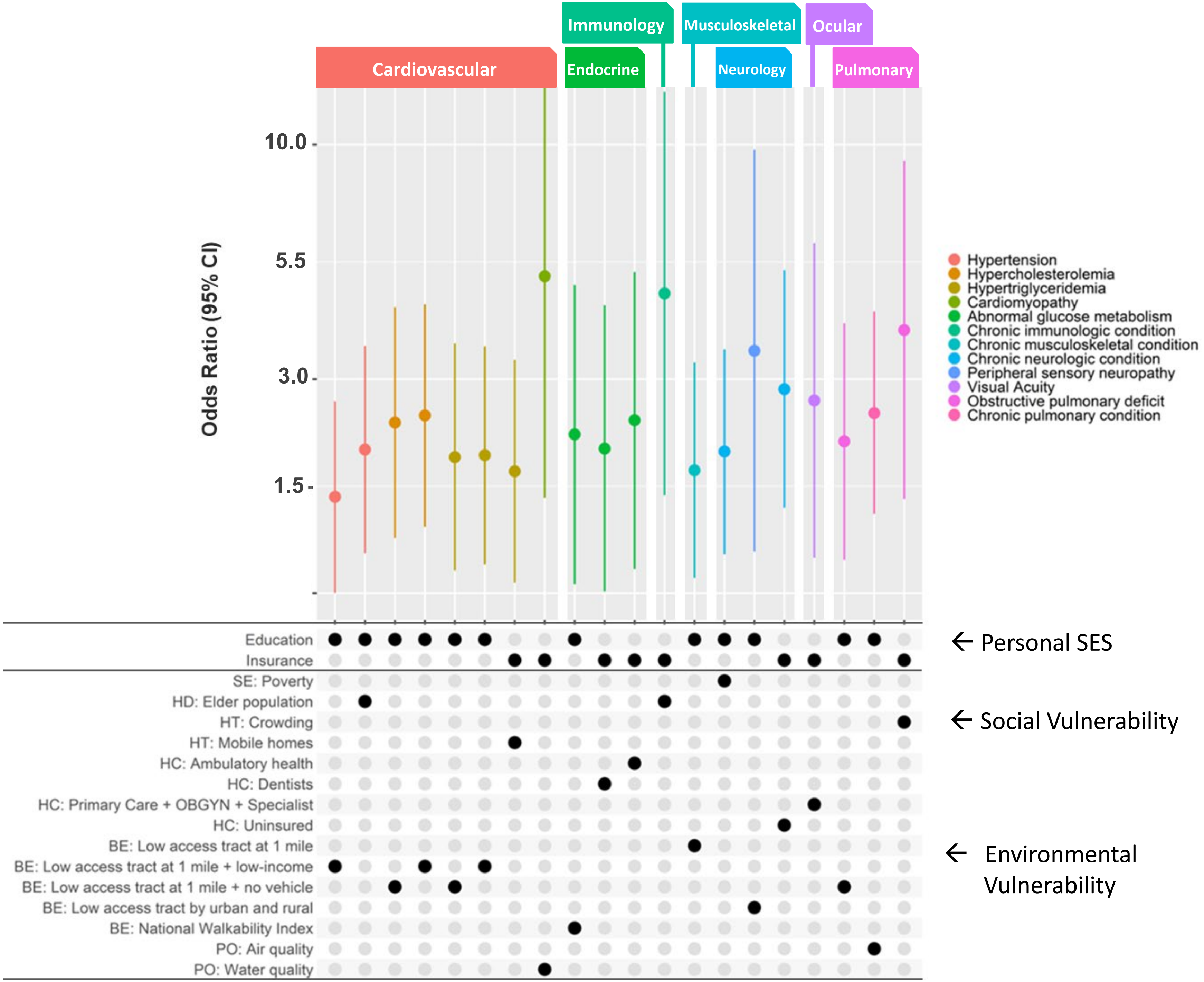

5a. Interactive Effect Between SDOH and Personal SES on CHC Persistent Status

Significant interaction ($p<0.05$) between SDOH (Q4 vs. Q1) and personal SES (lowest group [≤ high school; public insurance] vs. other groups) were shown.

Abbreviations:
BE, built environment domain;
HC, health care domain;
HC, health care domain;

HD, household composition/disability domain;
PO, pollution;
SE, socioeconomic domain

[a] OR adjusted with age at baseline, sex, cancer diagnosis, time from diagnosis to baseline, and smoking status

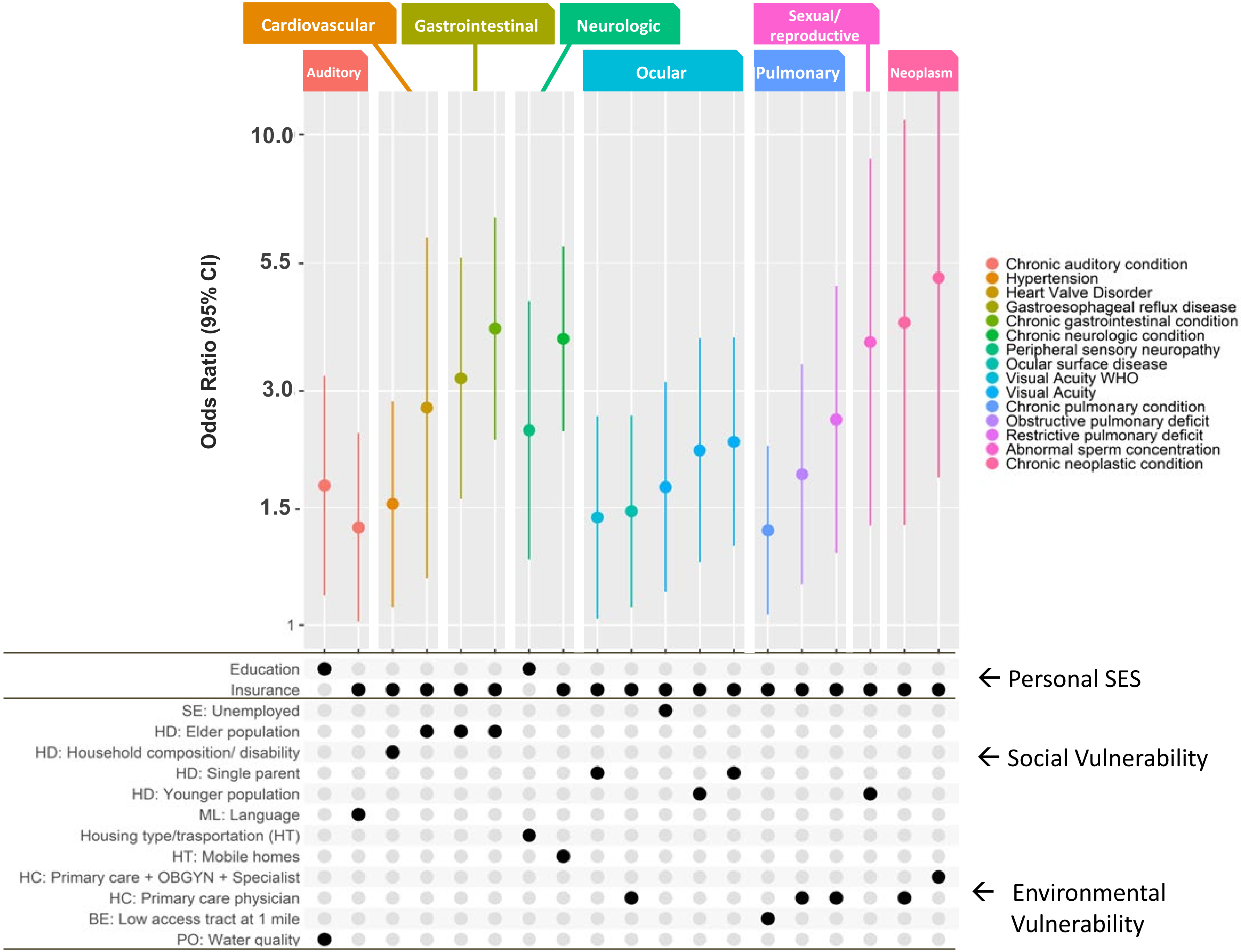

5b. Interactive Effect Between SDOH and Personal SES on CHC Worsening/Onset Status

Significant interaction (p<0.05) between SDOH (Q4 vs. Q1) and personal SES (lowest group [≤ high school; public insurance] vs. other groups) were shown.

Abbreviations:
BE, built environment;
HC, Health care;
HD, household composition/disability;
ML, minority status/language;
PO, pollution;
SE, socioeconomic

[a] OR adjusted with age at baseline, sex, cancer diagnosis, time from diagnosis to baseline, and smoking status

	Persistent	
	CHC group	Individual CHC
Social Vulnerability		
SVI overall	Endocrine	Obesity
SVI Socioeconomic status	Endocrine	Obesity
Poverty	Endocrine	Obesity
Income	Endocrine	Obesity
Diploma	Endocrine	Obesity
SVI Household composition/ disability	Endocrine	Obesity
Elder population	Cardiovascular	Cardiomyopathy
Disability	Cardiovascular	Hypertension
	Endocrine	Obesity
Single parent	Endocrine	Obesity
SVI Minority status/language		
Minority	Neurologic	Peripheral sensory neuropathy
	Sexual/reproductive	Abnormal sperm concentration
Language	Ocular	Visual acuity
SVI Housing type/transportation		
No vehicle	Cardiovascular	Cardiomyopathy
Group quarters	Cardiovascular	
	Neurologic	Peripheral motor neuropathy
Environmental Vulnerability		
Health care		
Primary care physician	Endocrine	Obesity
Primary care + OBGYN + specialist	Endocrine	Obesity
Ambulatory care	Cardiovascular	Hypercholesterolemia
	Endocrine	Obesity
Dentists	Cardiovascular	Hypercholesterolemia
	Endocrine	Obesity
	Pulmonary	Asthma
Uninsured	Endocrine	Obesity
	Ocular	
	Cardiovascular	Hypertension
Built environment		
Low access tract by urban and rural area	Endocrine	Obesity
	Pulmonary	Obstructive sleep apnea
Low access tract at 1 mile + no vehicle	Endocrine	Hypothyroidism Primary
Walkability	Endocrine	
Pollution		
Air quality	Cardiovascular	Hypercholesterolemia
		Hypertriglyceridemia
	Endocrine	Abnormal glucose metabolism

Summary of the significant associations of SDOH with persistent status of CHCs

- **Endocrine group: obesity, hypothyroidism, abnormal glucose metabolism**
- **Cardiovascular group: hypertension, cardiomyopathy, hypercholesterolemia, and hypertriglyceridemia**
- **Neurologic group: peripheral sensory neuropathy, peripheral motor neuropathy**
- **Sexual/reproductive group: Abnormal sperm concentration**
- **Ocular group: visual acuity**
- **Pulmonary: obstructive sleep apnea**

SDOH	Worsening/Onset CHC group	Individual CHC
Social Vulnerability		
SVI overall	Endocrine	Obesity
	Neurologic	Peripheral motor neuropathy
SVI Socioeconomic status	Endocrine	Obesity
	Neurologic	Peripheral motor neuropathy
Poverty	Neurologic	Peripheral motor neuropathy
Unemployed	Neurologic	Peripheral motor neuropathy
Diploma	Auditory	Hearing Loss
	Neurologic	Peripheral motor neuropathy
		Peripheral sensory neuropathy
	Endocrine	Abnormal glucose metabolism
	Ocular	Ocular surface disease
		Visual Acuity WHO
SVI Household composition/ disability	Auditory	Hearing loss
	Pulmonary	Obstructive pulmonary deficit
	Sexual/reproductive	
	Cardiovascular	Hypertension
	Endocrine	Abnormal glucose metabolism
	Neurologic	Peripheral motor neuropathy
Elder population	Gastrointestinal	
	Immunology	
	Pulmonary	Obstructive pulmonary deficit
Disability	Neurologic	Peripheral motor neuropathy
		Peripheral sensory neuropathy
	Pulmonary	
	Renal	
	Cardiovascular	Hypertension
Single parent	Endocrine	Abnormal glucose metabolism
		Obesity
SVI Minority status/language	Hematologic	
	Pulmonary	Asthma
Minority	Hematologic	
Language	Ocular	Visual Acuity WHO
		Visual Acuity
	Cardiovascular	Hypertension
	Pulmonary	Asthma

Overall/ Domain of the SVI	Worsening/Onset CHC group	Individual CHC
SVI Housing type/transportation	Endocrine	Obesity
	Neurologic	Peripheral motor neuropathy
Multi-unit	Muskuloskeletal	
Mobile homes	Auditory	Hearing Loss
	Gastrointestinal	Gastroesophageal reflux disease
	Neurologic	Peripheral sensory neuropathy
	Endocrine	Obesity
	Renal	Chronic kidney disease
Crowding	Auditory	Hearing Loss
	Endocrine	Obesity
	Pulmonary	Restrictive pulmonary deficit
No vehicle	Hematologic	
	Pulmonary	Asthma
		Obstructive pulmonary deficit
Environmental Veulnerability		
Health care		
Primary care physician	Cardiovascular	Cardiomyopathy
Ambulatory care	Endocrine	Abnormal glucose metabolism
Dentists	Immunology	
	Renal	
Uninsured	Auditory	Hearing Loss
	Cardiovascular	Hypertension
	Pulmonary	Restrictive pulmonary deficit
Built environment		
Low access tract at 1 mile	Gastrointestinal	Gastroesophageal reflux disease
Low access tract at 1 mile + no vehicle	Gastrointestinal	Gastroesophageal reflux disease
Low access tract at 1 mile + low-income	Endocrine	Abnormal glucose metabolism
		Obesity
	Gastrointestinal	Gastroesophageal reflux disease
Walkability	Endocrine	Abnormal glucose metabolism
		Obesity
	Gastrointestinal	Gastroesophageal reflux disease
Pollution		
Water quality	Endocrine	Hypothyroidism primary

Summary of the significant associations of SDOH with worsening/onset status of CHCs

- **Endocrine group: obesity, abnormal glucose metabolism**
- **Neurologic group: peripheral motor neuropathy, peripheral sensory neuropathy**
- **Pulmonary group: asthma, obstructive pulmonary deficit, restrictive pulmonary deficit; Cardiovascular: hypertension, cardiomyopathy**
- **Gastrointestinal group: gastroesophageal reflux disease**
- **Auditory group: visual acuity; Ocular: visual acuity; Renal: chronic kidney disease**
- **Hematologic; Immunology; Musculoskeletal; Sexual/reproductive group**

Next Steps

St. Jude LIFE
St. Jude for Life

- Further Analysis
  - Adjusting the specific treatment (e.g., anthracycline in cardiovascular group analysis) for specific CH Cs per COG guidelines and Hudson, et al, 2021 Pediatrics
- Future Research Direction
  - Identifying actionable clinical/social interventions targeting specific SDOH
    - Providing evidence-based implications for healthcare providers to prevent or decrease the impact of social/environmental vulnerability on CHC burden
  - Creating a predicting model by including comprehensive SDOH to enhance the performance of existing models
